# Context-Dependent modulation of astrocytic Ca^2+^ signals by mitochondria: a computational study

**DOI:** 10.1007/s10827-026-00956-3

**Published:** 2026-09-25

**Authors:** Thiago Ohno Bezerra, Antonio C. Roque

**Affiliations:** Department of Physics, School of Philosophy, Sciences and Letters of Ribeirão Preto, University of São Paulo, Bandeirantes avenue, Ribeirão Preto, 14040-900 São Paulo Brazil

**Keywords:** Astrocytes, Glia, Calcium signaling, Mitochondria

## Abstract

Mitochondria regulate intracellular Ca^2+^ by uptake through the mitochondrial Ca^2+^ uniporter (MCU) and release via the mitochondrial permeability transition pore (mPTP). In astrocytes, neurotransmitter stimulation evokes Ca^2+^ signaling, yet the role of mitochondria in shaping these responses remains unclear. We extended a compartmental astrocyte model developed by our group to include MCU- and mPTP-mediated dynamics, and simulated glutamatergic and dopaminergic inputs modeled as Poisson processes driving IP_3_ synthesis through the phospholipase-C (PLC) pathway. Both unipolar and bifurcated-terminal morphologies were considered, with mitochondria positioned in alternating compartments starting from the soma; distal compartments contained mitochondria only when sufficiently large. Simulations show that mitochondria modulate Ca^2+^ signaling in a context-dependent manner: under weak glutamatergic input, they reduce oscillation frequency and limit signal propagation, whereas under strong glutamatergic input or dopaminergic modulation, they enhance Ca^2+^ responses by reducing Ca^2+^-dependent IP_3_ degradation. These results suggest that mitochondria can play an important role in shaping the spatial organization of Ca^2+^ signaling in astrocytes.

**Supplementary Information:**

The online version contains supplementary material available at https://doi.org/10.1007/s10827-026-00956-3.

## Introduction

Mitochondria are central to cellular metabolism (Filadi & Greotti, 2021). Intramitochondrial Ca^2+^ is a major regulator of mitochondrial metabolism, being associated with the modulation of both oxidative phosphorylation and ATP synthesis (Filadi & Greotti, 2021). Beyond their metabolic role, mitochondria also contribute significantly to Ca^2+^ activity in astrocytes (Agarwal et al., 2017; Serrat et al., 2021).

Astrocytes, traditionally regarded as supportive cells providing metabolic substrates, regulating neurotransmitter homeostasis, and maintaining extracellular ion balance (Verkhratsky & Nedergaard, 2018), are now recognized as active modulators of neural function. They participate in excitatory–inhibitory balance (Noh et al., 2023), synaptic plasticity (Shelkar et al., 2022), neural oscillations (Fellin et al., 2009; Lee et al., 2014), and sensory processing (Reitman et al., 2023; Schummers et al., 2008), thereby influencing memory, social behavior, and motor responses (Lee et al., 2014; Mu et al., 2019; Noh et al., 2023). Astrocytes express a wide range of neurotransmitter and neuromodulator receptors, including metabotropic glutamate, *α *_1_-adrenergic, and D_1_/D_2_ dopaminergic receptors (Bezerra & Roque, 2024; Corkrum et al., 2020; Reitman et al., 2023; Semyanov, 2019; Verisokin et al., 2023). These activate the phospholipase-C (PLC) pathway, promoting IP_3_ synthesis via a Ca^2+^-sensitive mechanism. IP_3_ then opens IP_3_ receptors on the ER membrane, releasing Ca^2+^ into the cytosol. Signal termination occurs either by receptor closure or Ca^2+^-dependent IP_3_ degradation. Additional regulators of cytosolic Ca^2+^ include ion channels such as NMDA and transient receptor potential channels (Goenaga et al., 2023; Rungta et al., 2016; Semyanov, 2019; Shelkar et al., 2022), as well as transporters such as the Na^+^/Ca^2+^ exchanger and plasma-membrane Ca^2+^ ATPase (Poejo et al., 2023; Reyes et al., 2012; Rojas et al., 2007; Ziemens et al., 2019).

Astrocytic Ca^2+^ activity exhibits diverse temporal and spatial patterns, including spontaneous or elicited signals (Goenaga et al., 2023) and local or global responses (Bezerra & Roque, 2024; Bindocci et al., 2017; Goenaga et al., 2023). Global elicited responses are typically driven by neuromodulators such as noradrenaline and dopamine (Bezerra & Roque, 2024; Verisokin et al., 2023), whereas local responses often result from synaptic glutamate release, mitochondrial activity, or plasma-membrane ion fluxes (Bezerra & Roque, 2024; Goenaga et al., 2023; Oschmann et al., 2017; Rungta et al., 2016; Semyanov, 2019).

Dopamine-evoked Ca^2+^ signaling in astrocytes has been implicated in several brain functions, including the psychomotor effects of amphetamine, locomotion, and social behavior (Corkrum et al., 2020; Li et al., 2023; Requie et al., 2022). Dopamine therefore provides a biologically relevant example of a diffuse neuromodulatory signal that can interact with local glutamatergic inputs at the level of astrocytic Ca^2+^ signaling.

Astrocytic Ca^2+^ signals can trigger gliotransmitter release (e.g., glutamate, ATP, D-serine) (Covelo & Araque, 2018; De Ceglia et al., 2023; Goenaga et al., 2023), positioning astrocytic Ca^2+^ signaling as a key mechanism for modulating neural activity and integrating information from different sources, such as local glutamatergic and global dopaminergic inputs. In addition to the modulation of synaptic transmission, Ca^2+^ signals are also associated with the regulation of neurovascular coupling (Lind et al., 2018; Tesler et al., 2023). Thus, astrocytic Ca^2+^ signaling contributes to diverse brain functions, highlighting the importance of understanding the mechanisms that regulate these cellular responses.

Mitochondria further shape these responses (Agarwal et al., 2017; Cabral-Costa & Kowaltowski, 2023; Serrat et al., 2021). Ca^2+^ enters mitochondria primarily through the mitochondrial Ca^2+^ uniporter (MCU) (Cabral-Costa & Kowaltowski, 2023; Csordás et al., 2010; Serrat et al., 2021), reaching concentrations of tens of µM (Csordás et al., 2010). Ca^2+^ efflux occurs via the Na^+^/Ca^2+^ exchanger (NCLX) and the mitochondrial permeability transition pore (mPTP) (Agarwal et al., 2017; Cabral-Costa & Kowaltowski, 2023; Parnis et al., 2013). While prolonged mPTP opening, often linked to Ca^2+^ overload and redox imbalance, can trigger cell death (Cabral-Costa & Kowaltowski, 2023), transient openings under physiological conditions appear to regulate homeostasis (Bernardi et al., 2023). mPTP (Agarwal et al., 2017) and MCU (Serrat et al., 2021) activity have both been implicated in astrocytic Ca^2+^ signaling. Mitochondria are frequently positioned near ER domains, forming mitochondria–ER contact sites (MERCS) that contribute to local Ca^2+^ regulation (Cabral-Costa & Kowaltowski, 2023; Csordás et al., 2010; Serrat et al., 2021). ER abundance and organization vary substantially across astrocytic subcellular compartments, including fine perisynaptic processes (Aboufares El Alaoui et al., 2021; Benoit et al., 2025; Patrushev et al., 2013). Moreover, mitochondria are mobile and can migrate toward regions of high Ca^2+^ activity or glutamate uptake, thereby dynamically altering local Ca^2+^ buffering (Cabral-Costa & Kowaltowski, 2023; Jackson & Robinson, 2015). Dysregulation of astrocytic and mitochondrial function has been associated with aging (Popov et al., 2023), neurodegenerative diseases including Alzheimer’s and Parkinson’s disease (Bantle et al., 2021; Preman et al., 2021), and psychiatric disorders such as schizophrenia (Kolomeets & Uranova, 2010; Roberts, 2021; Uranova et al., 1996). These observations further motivate investigation of how mitochondrial Ca^2+^ handling shapes astrocytic signaling.

Given the complexity of astrocytic Ca^2+^ signaling (Semyanov, 2019), computational models have been developed to analyze its underlying mechanisms. These models fall into three categories: (i) De Young–Keizer and Li–Rinzel–type models, which describe Ca^2+^ release from ER stores via IP_3_ receptors; (ii) Höfer-type reaction–diffusion models, which incorporate spatial dynamics of Ca^2+^ propagation; and (iii) hybrid approaches (Manninen et al., 2018). These models simulate the basic mechanisms involved in Ca^2+^ release from the ER through IP_3_R channels and the uptake by the SERCA pump (De Young & Keizer, 1992; Höfer et al., 2002; Li & Rinzel, 1994). They are well-established for the simulation of Ca^2+^ signaling in astrocytes and form the basis for most computational models studying astrocytic response. However, only a few models have examined mitochondrial contributions. Komin et al. (2015) simulated the Ca^2+^ dynamics including a stochastic model of the IP_3_R (Christian et al., 2014; Skupin et al., 2010) and the mitochondrial Ca^2+^ release but did not specify the efflux mechanism or test its impact on astrocytic responses. Diekman et al. (2013), building on Magnus and Keizer (1997) and Oster et al. (2011), investigated the neuroprotective role of mitochondrial metabolism during stroke, incorporating MCU-mediated influx and NCX-mediated efflux. Yet mitochondrial Ca^2+^ handling was not the primary focus. Overall, previous models have not addressed how mitochondria shape astrocytic Ca^2+^ signals or how these interactions depend on neurotransmitter input or astrocyte morphology (Bezerra & Roque, 2024).

Here, we present a compartmental astrocyte model extending our previous work (Bezerra & Roque, 2024) by explicitly including MCU and mPTP mechanisms. Using this model, we investigate how glutamatergic and dopaminergic inputs interact with mitochondrial dynamics. We show that mitochondria regulate astrocytic Ca^2+^ signaling in a context-dependent manner, suppressing responses to weak glutamatergic stimulation while enhancing responses to strong glutamatergic input or dopamine. This enhancement arises from reduced Ca^2+^-dependent IP_3_ degradation. Finally, we demonstrate how mitochondria gate the propagation of Ca^2+^ signals from distal astrocytic processes.

## Methods

The present model extends a previously developed compartmental astrocyte model from our group (Bezerra & Roque, 2024), incorporating features from earlier computational studies of astrocytic Ca^2+^ signaling and mitochondrial Ca^2+^ dynamics (De Pittà et al., 2009; Friel & Tsien, 1994; Gordleeva et al., 2019; Oschmann et al., 2017; Pivovarova et al., 1999; Ullah et al., 2006). In the first subsection, we introduce a single compartment and detail the variables and currents associated with mitochondrial mechanisms. We then describe the interconnection of compartments, the astrocytic morphologies considered, and the distribution of mitochondria. Parameters, additional variables and currents, and stimulation protocols are provided, respectively, in Online Resources 1, 2 and 3.

### Single compartment model

**Fig. 1:**
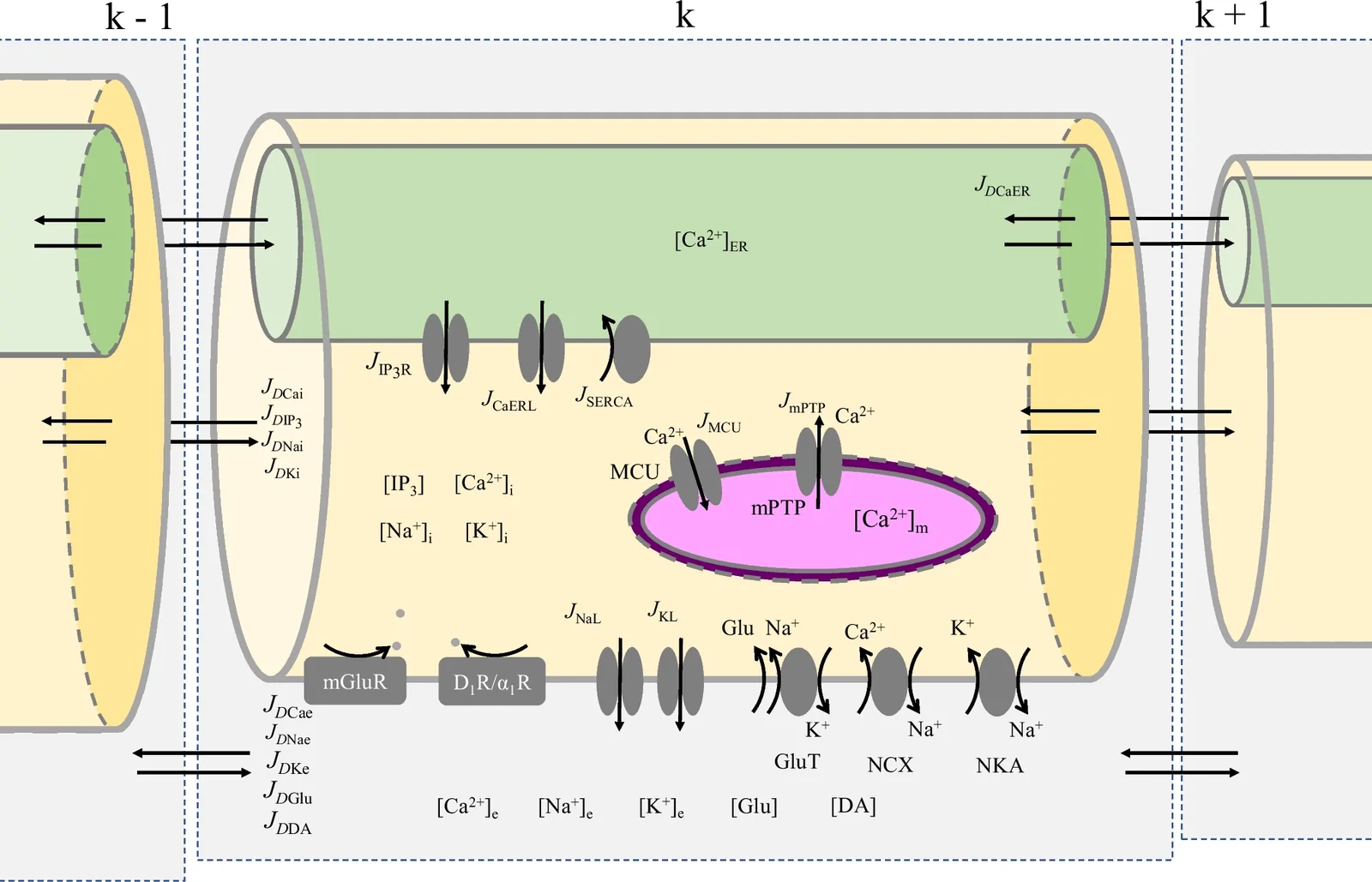
Schematic representation of compartment variables and currents. Each compartment is subdivided into extracellular (gray), intramitochondrial (purple), cytosolic (yellow), and intra-ER (green) spaces. Cytosolic Ca^2+^ ($\mathrm {[Ca^{2+}]_i}$) is regulated by ER release through IP_3_R channels ($J_\mathrm {IP_3R}$), ER leak ($J_\textrm{CaERL}$), SERCA uptake ($J_\textrm{SERCA}$), mitochondrial uptake through MCU ($J_\textrm{MCU}$), mitochondrial release via mPTP ($J_\textrm{mPTP}$), plasma-membrane NCX ($J_\textrm{NCX}$), and diffusion between compartments ($J_{D\textrm{Cai}}$). Extracellular Ca^2+^ ($\mathrm {[Ca^{2+}]_e}$) is modulated by NCX and diffusion ($J_{D\textrm{Cae}}$). ER subcompartments are linked by Ca^2+^ diffusion currents ($J_{D\textrm{CaER}}$). Mitochondrial Ca^2+^ ($\mathrm {[Ca^{2+}]_m}$) is regulated by MCU and mPTP. Cytosolic IP_3_ ($\mathrm {[IP_3]}$) is generated by PLC*β * activation via mGluR, D_1_R, and *α *_1_R receptors, as well as PLC*δ *, and degraded by IP_3_-3K and IP-5P, with additional regulation through diffusion ($J_{D\mathrm {IP_3}}$). Cytosolic and extracellular Na^+^/K^+^ levels are governed by leak currents ($J_\textrm{NaL}$, $J_\textrm{KL}$), glutamate transporter ($J_\textrm{GluT}$), NCX, NKA ($J_\textrm{NKA}$), and diffusion ($J_{D\textrm{Nai}}$, $J_{D\textrm{Nae}}$, $J_{D\textrm{Ki}}$, $J_{D\textrm{Ke}}$). Extracellular glutamate and dopamine concentrations are determined by presynaptic release events (modeled as Poisson processes) and by diffusion across extracellular subcompartments ($J_{D\textrm{Glu}}$, $J_{D\textrm{DA}}$)

Each compartment was subdivided into intra-ER, intramitochondrial, cytosolic, and extracellular spaces (Fig. 1). These subspaces were modeled as well-mixed solutions and coupled by current densities. The following variables were considered: cytosolic and extracellular Ca^2+^ concentrations ($\mathrm {[Ca^{2+}]_i}$ and $\mathrm {[Ca^{2+}]_e}$); ER Ca^2+^ concentration ($\mathrm {[Ca^{2+}]_{ER}}$); mitochondrial Ca^2+^ concentration ($\mathrm {[Ca^{2+}]_m}$); cytosolic IP_3_ concentration ($\mathrm {[IP_3]}$); the fraction of open IP_3_ channels (*h*); cytosolic and extracellular Na^+^ and K^+^ concentrations ($\mathrm {[Na^+]_i}$, $\mathrm {[Na^+]_e}$, $\mathrm {[K^+]_i}$, $\mathrm {[K^+]_e}$); the compartment potential (*v*); and extracellular glutamate ($\mathrm {[Glu]}$) and dopamine ($\mathrm {[DA]}$) concentrations. With the exception of the rate equations for cytosolic and mitochondrial Ca^2+^ concentrations, and the associated inter-subspace currents described below, all other equations, current densities, and parameter definitions follow our previous model (Bezerra & Roque, 2024).

The cytosolic and mitochondrial Ca^2+^ dynamics are described by:

1 $$\begin{aligned} \frac{d\mathrm {[Ca^{2+}]_i}}{dt}&= \frac{A}{VF} J_{\textrm{NCX}} + \frac{A \sqrt{r_\textrm{ER}}}{VF} (J_\mathrm {IP_3R} \nonumber \\&\quad - J_\textrm{SERCA} + J_\textrm{CaERL})~+ \end{aligned}$$

2 $$\begin{aligned}&\quad \frac{A \sqrt{r_\textrm{m}}}{VF} ( J_\textrm{mPTP} - J_\textrm{MCU}), \nonumber \\ \frac{d\mathrm {[Ca^{2+}]_m}}{dt}&= \frac{A \sqrt{r_\textrm{m}}}{VFr_\textrm{m}} \left( J_\textrm{MCU} - J_\textrm{mPTP} \right) , \end{aligned}$$

where *A* is the lateral surface area of the compartment, *V* its volume, *F* Faraday’s constant, $r_\textrm{ER}$ and $r_\textrm{m}$ the ER-to-cytosol and mitochondria-to-cytosol volume ratios, respectively, and $J_\textrm{NCX}$, $J_\mathrm {IP_3R}$, $J_\textrm{SERCA}$, $J_\textrm{CaERL}$, $J_\textrm{mPTP}$, and $J_\textrm{MCU}$ the corresponding current densities. The lateral surface areas of the ER and mitochondrial subcompartments are $A \sqrt{r_\textrm{ER}}$ and $A \sqrt{r_\textrm{m}}$, respectively, as derived in Eq. S30 of Online Resource 1. Ca^2+^ release from the ER through the IP_3_R is dependent on the cytosolic $\mathrm {[IP_3]}$ and the fraction of open IP_3_R channels *h*, which is inhibited by the cytosolic Ca^2+^ and models the channel inactivation. This mechanism creates a loop that generates the Ca^2+^ response in the model.

The MCU and mPTP current densities are given by:

3 $$\begin{aligned} J_\textrm{MCU}&= \frac{F V}{A} V_\textrm{MCUmax} \frac{\mathrm {[Ca^{2+}]_i}^2}{\mathrm {[Ca^{2+}]_i}^2 + K_\textrm{MCU}^2} \mathrm {[Ca^{2+}]_i}, \end{aligned}$$

4 $$\begin{aligned} {\begin{matrix} J_\textrm{mPTP} & = \frac{F V}{A} \bigl [ V_\textrm{mPTPmax} \frac{\mathrm {[Ca^{2+}]_m}}{\mathrm {[Ca^{2+}]_m} + K_\textrm{mPTP}} +\\ & \quad l_\textrm{mPTP} (\mathrm {[Ca^{2+}]_i} - \mathrm {[Ca^{2+}]_m}) \bigr ] \end{matrix}}, \end{aligned}$$

where $V_{\textrm{MCUmax}}$ is the maximum MCU uptake rate constant, $K_{\textrm{MCU}}$ is the MCU Ca^2+^ affinity constant, $V_{\textrm{mPTPmax}}$ and $K_{\textrm{mPTP}}$ are the maximum mPTP-mediated efflux rate and Ca^2+^ affinity constant, respectively, and $l_{\textrm{mPTP}}$ represents passive Ca^2+^ efflux through mPTP. This mitochondrial model follows Pivovarova et al. (1999) and Friel and Tsien (1994), with parameter adjustments for astrocytes as detailed in Online Resource 1. This reduced mitochondrial formulation does not explicitly model mitochondrial membrane potential, redox state, or other ionic species that modulate MCU and mPTP activity; their effects are therefore represented only implicitly through the effective parameters of the model.

To account for regional variability in ER and mitochondrial size (Aboufares El Alaoui et al., 2021; Patrushev et al., 2013; Salazar et al., 2024; Salmon et al., 2023), we introduced $r_\textrm{ER}$ and $r_\textrm{m}$ as volume fractions of ER and mitochondria relative to cytosol. $r_\textrm{ER}$ is given by:

5 $$\begin{aligned} r_{ER} = a e^{-(b A_i/V_i)^{c}}, \end{aligned}$$

where *a = 0.15*, *b = 0.073* µm, *c = 2.34* (fitted from experimental data Bezerra & Roque, 2024; Patrushev et al., 2013), and $A_i/V_i$ is the surface-to-volume ratio of the compartment.

For mitochondria, the total volume within a compartment was approximated as a cylinder of fixed length *l = 1* µm (matching compartment length) and radius $\phi _m$, defined as a function of the compartment radius *Φ *:

6 $$\begin{aligned} \phi _m(\Phi ) = \frac{a}{1 + \exp \left( b \Phi - c \right) } + d, \end{aligned}$$

where *a*, *b*, *c*, and *d* are free parameters fitted to mitochondrial thickness data from adult mouse somatosensory cortical astrocytes (Salmon et al., 2023). The mitochondria-to-cytosol volume ratio is then $r_\textrm{m} = V_m/V_i$. Details of parameter fitting are provided in Online Resource 1.

### Compartment coupling and cell morphologies

Compartments were coupled by diffusive fluxes of Ca^2+^, Na^+^, K^+^, IP_3_, glutamate, and dopamine between the cytosolic, extracellular, and ER subspaces of adjacent compartments. Mitochondria were modeled as isolated subcompartments, and we did not include mitochondrial networks or complexes reported in some studies (Salazar et al., 2024). The rate of change of the concentration of ion/molecule *X* in compartment *j* due to diffusion from a neighboring compartment *k* was given by Goldberg et al. (2010):

7 $$\begin{aligned} \left( \frac{d[\textrm{X}]_j}{dt} \right) _{\text {diff}} = F_{\textrm{X}} \left( [\textrm{X}]_k - [\textrm{X}]_j \right) , \end{aligned}$$

where $F_{\textrm{X}}$ (s^-1^) is the coupling strength, determined by compartment geometry and the diffusion coefficient $D_{\textrm{X}}$ (Bezerra & Roque, 2024). Full details of the diffusive fluxes and parameters are provided in Online Resources 1 and 3.

We first simulated a unipolar morphology, consisting of a spherical somatic compartment followed by 20 cylindrical compartments (Table S3 in Online Resource 2), representing a single astrocytic process (Bezerra & Roque, 2024). For comparison, we also implemented a bifurcated-terminal morphology (24 compartments; Fig. 9**a**, **b**; Tables S4 and S5 in Online Resource 2), in which a linear process emerging from the soma bifurcates near its distal end.

Because mitochondria are not uniformly distributed in astrocytes (Salazar et al., 2024; Salmon et al., 2023), in our model they were placed in alternating compartments, starting from the soma (i.e., present in every other compartment). Distal compartments with radii below 0.2 µm were assumed to lack mitochondria, since these processes are thinner than mitochondrial thickness. For the bifurcated morphology, we tested two variants: one with mitochondria positioned at the bifurcation point and one without. Further details of the morphological models are provided in  Online Resources 1 and 2.

### Model input and output

We simulated both glutamatergic and dopaminergic transmission to astrocytes. These stimuli activate the PLC pathway, leading to IP_3_ synthesis and Ca^2+^ release from the ER (Bezerra & Roque, 2024; Huntington & Srinivasan, 2021; Liu et al., 2009; Pittolo et al., 2022). Glutamate also drives Ca^2+^ uptake via the glutamate transporter (GluT), which alters the Na^+^ gradient and activates the NCX (Fig. 1) (Bezerra & Roque, 2024; Lalo et al., 2021; Oschmann et al., 2017; Semyanov, 2019). Both inputs were modeled as Poisson processes with constant frequencies $\nu _g$ (glutamate; increment $\rho _\textrm{glu}$ = 0.5 µM) and $\nu _d$ (dopamine; increment $\rho _\textrm{DA}$ = 3 µM), expressed in Hz. The input frequencies explored here were chosen to span ranges compatible with reported glutamatergic and dopaminergic neuronal activity (Constantinidis & Goldman-Rakic, 2002; Floresco et al., 2003; Grace & Bunney, 1984). These Poisson inputs are intended as phenomenological representations of transmitter release rather than direct reproductions of a specific experimental stimulation protocol. Stimulated compartments are denoted CX–Y, where X and Y indicate the first and last stimulated compartments. Unless otherwise specified, glutamate was applied to the distal compartments (C18–20), mimicking localized tripartite synapse inputs (Lalo et al., 2021), while dopamine was applied globally (C1–20), representing the diffuse nature of neuromodulatory transmission (Liu et al., 2021). Exceptions were made in tests where the positions of glutamatergic input and mitochondria were varied (Figs. S10 and S12).

Ca^2+^ signals were analyzed in terms of oscillation frequency ($f_\mathrm {Ca^{2+}}$) and spatial extent. Ca^2+^ signals were counted whenever cytosolic Ca^2+^ exceeded a threshold of $\mathrm {[Ca^{2+}]_{th}} = 0.15$ µM (Bezerra & Roque, 2024; Gordleeva et al., 2019). Oscillation frequency was defined as the number of threshold crossings during stimulation, normalized by the trial duration (*t = 100* s). Signal propagation was quantified as the number of consecutive compartments activated, counted from the terminal end. Throughout the manuscript, an individual threshold-crossing transient is referred to as a Ca^2+^ signal or event. Repeated Ca^2+^ signals during a stimulation period are referred to as Ca^2+^ oscillations and are quantified by $f_\mathrm {Ca^{2+}}$. We use the broader term Ca^2+^ response to describe the overall activity elicited by a stimulation condition, including signal frequency, amplitude, and propagation extent.

For parameter-sensitivity analyses, changes in an output *X* were also quantified relative to a fixed reference response of the model without mitochondria. For a parameter *p*, this measure was defined as $\Delta _{\textrm{ref}} X(p) = X_{\textrm{mito}}(p) - X_{\mathrm {no \; mito}}(p_{\textrm{ref}})$, where $p_{\textrm{ref}}$ is the default parameter value. Thus, $\Delta _{\textrm{ref}} X(p) = 0$ indicates recovery of the reference response, whereas positive and negative values indicate responses above and below this reference, respectively. This fixed-reference measure is distinct from a pointwise difference between models evaluated at the same parameter value. Further details are provided in Online Resource 1 (see Eq. S31).

**Fig. 2:**
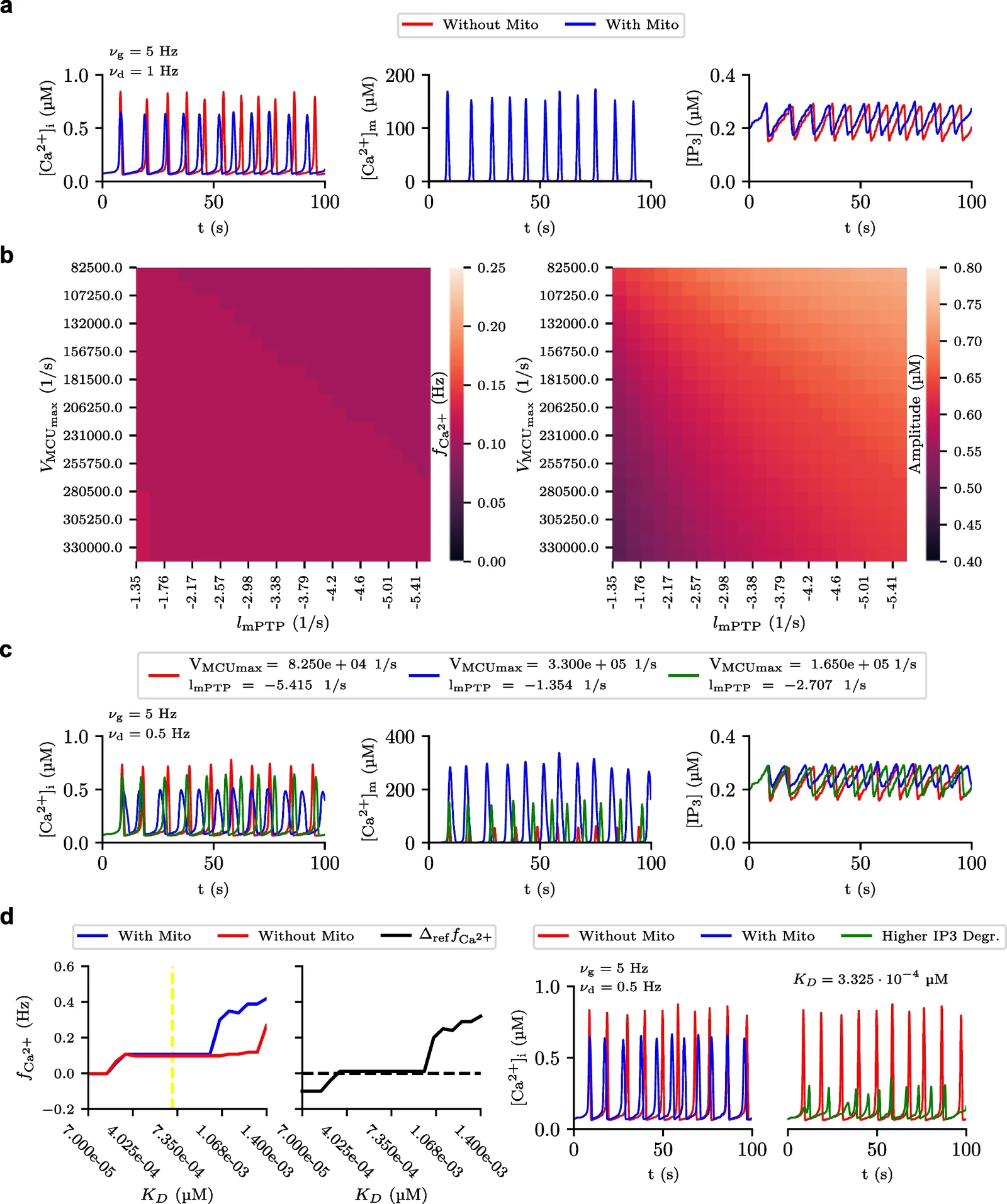
Mitochondria enhance astrocytic response by modulating Ca^2+^-dependent IP_3_ degradation. Single-compartment model with glutamatergic ($\nu _g$ = 5 Hz, C1) and dopaminergic ($\nu _d$ = 1 Hz, C1) inputs. (**a**) cytosolic and intramitochondrial Ca^2+^, and cytosolic IP_3_ concentrations. (**b**) Frequency of Ca^2+^ signals ($f_{\mathrm {Ca^{2+}}}$) and average amplitude of Ca^2+^ signals. (**c**) Cytosolic and intramitochondrial Ca^2+^, and cytosolic IP_3_ concentrations for three combinations of MCU maximum Ca^2+^ uptake rate ($\mathrm {V_{maxMCU}}$) and mPTP release rate ($\mathrm {l_{mPTP}}$). (**d**) Left panel: frequency of Ca^2+^ signals ($f_\mathrm {Ca^{2+}}$) for the models with and without mitochondria as a function of $K_D$, together with the deviation from the fixed non-mitochondrial reference, $\Delta _{\textrm{ref}} f_\mathrm {Ca^{2+}}(K_D)$. The vertical yellow dashed line indicates the reference value $K_D^{\textrm{ref}}=0.7$ µM. The corresponding value of $f_\mathrm {Ca^{2+}}$ in the model without mitochondria defines the fixed reference used to calculate $\Delta _{\textrm{ref}} f_\mathrm {Ca^{2+}}$. Right panel: Example of cytosolic Ca^2+^ concentration traces for the model without mitochondria, with mitochondria and the model with higher IP_3_ degradation

## Results

### Single compartment model

To compare the model responses with and without the mitochondrial mechanisms, we first simulated a single compartment model (radius *ϕ * = 2 µm; Table S1 in Online Resource 2) under glutamatergic ($\nu _g$ = 5 Hz) and dopaminergic inputs ($\nu _d$ = 1 Hz). This stimulation triggered Ca^2+^ signals in the astrocyte (Fig. 2**a**). Interestingly, mitochondria reduced the Ca^2+^ signal amplitude but increased the oscillation frequency (Fig. 2**a**). Cytosolic Ca^2+^ signals in the model with mitochondria are followed by intramitochondrial Ca^2+^ signals and by a higher frequency of IP_3_ oscillations compared to the model without mitochondria (Fig. 2**a**). Mitochondria also increased the minimum IP_3_ concentration reached after each triggered Ca^2+^ signal, keeping the average IP_3_ concentration higher compared to the model without mitochondria.

To explore the association between the frequency and amplitude of Ca^2+^ signals, we simulated the single compartment model with different combinations of maximum Ca^2+^ uptake rate by the MCU ($\mathrm {V_{MCUmax}}$) and release rate by the mPTP ($\mathrm {l_{mPTP}}$). In this test, lower frequencies of Ca^2+^ signals are associated with higher amplitudes of these signals (Fig. 2**b**,**c**). Average IP_3_ concentration is higher with high Ca^2+^ uptake but slower release, while the opposite is seen with slower uptake but faster release (Fig. 2**c**). Together, these results suggest that mitochondria enhance the Ca^2+^ response in astrocytes by reducing the amplitude of Ca^2+^ signals and increasing the average cytosolic IP_3_ concentration.

In this model, Ca^2+^ exerts inhibitory feedback on Ca^2+^ release from the ER by (i) inactivating the IP_3_R channels, a process controlled by the variable *h*; (ii) inhibiting the synthesis of IP_3_ by PLC; and (iii) promoting the degradation of IP_3_ by the IP_3_-3K. Changing the parameters associated with each of these processes, the Ca^2+^-dependent degradation of IP_3_, controlled by the Ca^2+^ affinity of IP_3_-3K ($K_D$), had a major effect on the astrocytic response (Fig. 2**d** and Fig. S3). The model with mitochondria exhibited one additional Ca^2+^ signal during the simulation period, corresponding to an 11% increase in $f_{\mathrm {Ca^{2+}}}$ relative to the model without mitochondria (Fig. 2**d**). Decreasing the Ca^2+^-dependent degradation of IP_3_ increasing $K_D$ potentiates the effect of mitochondria. In contrast, increasing the degradation of IP_3_ by decreasing $K_D$ reduced the $f_{\mathrm {Ca^{2+}}}$ in the model with mitochondria (Fig. 2**d**). Although decreasing $K_D$ in the model with mitochondria brings $f_{\mathrm {Ca^{2+}}}$ closer to that of the model without mitochondria, this change also reduces the amplitude of the Ca^2+^ signals. Thus, regulation of Ca^2+^-dependent degradation of IP_3_ may be one of the mechanisms modulated by mitochondria.

### Unipolar morphology

**Fig. 3:**
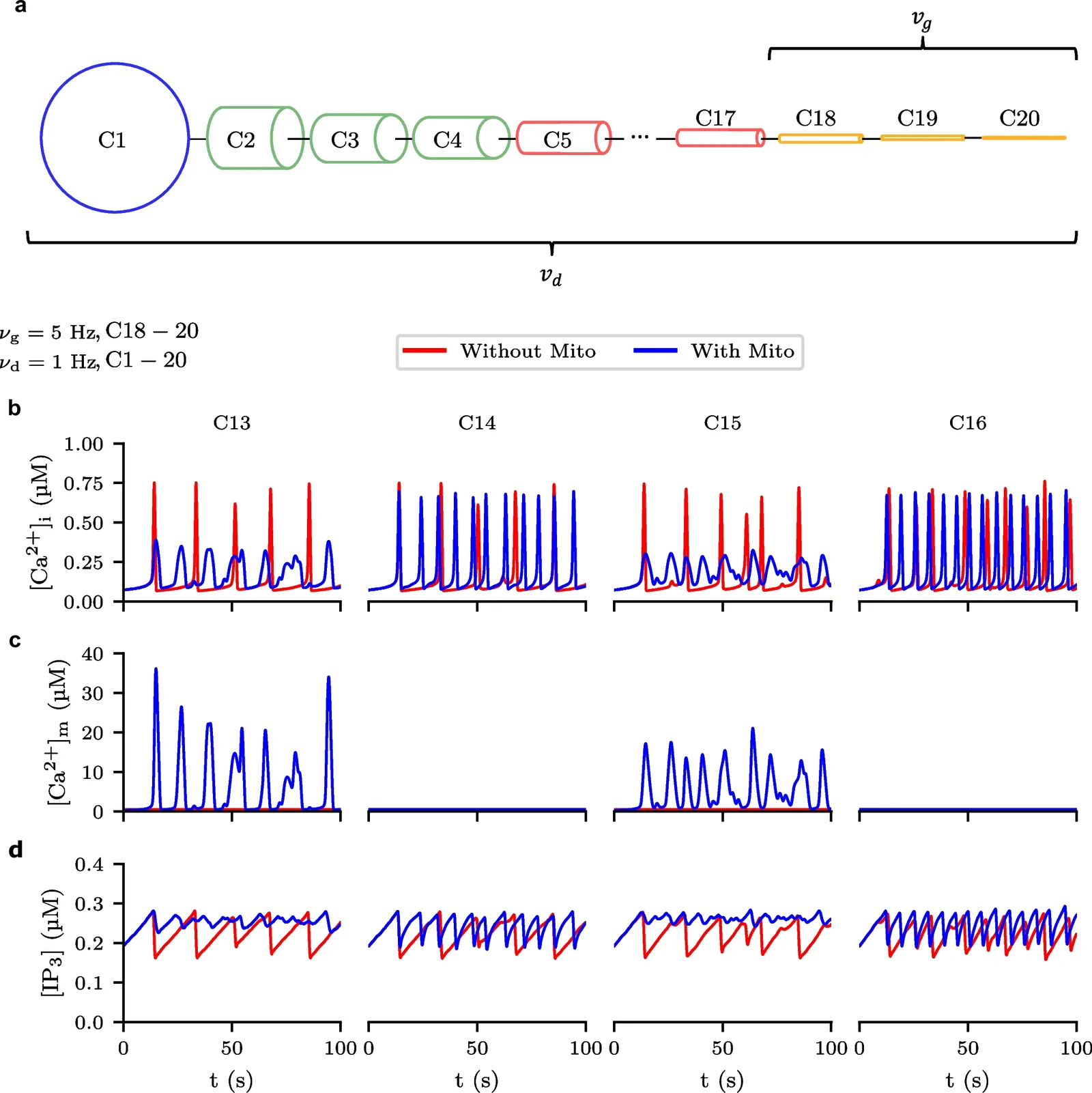
Glutamatergic and dopaminergic stimulation trigger Ca^2+^ signals in astrocyte models. C1 denotes the soma, and C2–C20 denote successive process compartments from proximal to distal. Glutamatergic stimulation is applied to C18–C20, whereas dopaminergic stimulation is applied to C1–C20 unless otherwise stated. Compartments C13 and C15 have mitochondria. (**a**) Model morphology and input locations. (**b**) Cytosolic Ca^2+^ concentration. (**c**) Intramitochondrial Ca^2+^ concentration. (**d**) Cytosolic IP_3_ concentration

To compare model responses with and without mitochondrial mechanisms, we simulated the unipolar astrocyte model under glutamatergic ($\nu _g$ = 5 Hz, C18–20) and dopaminergic ($\nu _d$ = 1 Hz, C1–20) stimulation (Fig. 3**a**). The analysis focused on compartments C13–16; results for the remaining compartments (Fig. S2) and for single-neurotransmitter stimulation (Fig. S3) are provided in Online Resource 1.

Both glutamatergic and dopaminergic inputs elicited Ca^2+^ signals across all compartments in both models (Fig. 3, Fig. S2**a**). Compartments containing mitochondria displayed smaller Ca^2+^ amplitudes but higher oscillation frequencies ($f_\mathrm {Ca^{2+}}$). Cytosolic Ca^2+^ signals were followed by corresponding increases in mitochondrial Ca^2+^ (Fig. 3**c**). Notably, IP_3_ concentrations were consistently higher in the model with mitochondria, indicating that mitochondrial activity delayed IP_3_ degradation during Ca^2+^ signaling (Fig. 3**d**). In the Ca^2+^–IP_3_ phase space, this was reflected by reduced Ca^2+^ oscillation amplitudes but elevated IP_3_ levels (Fig. S2**b**). Mitochondria also enhanced responses to glutamate alone ($\nu _g$ = 5 Hz; Fig. S3**a**) or dopamine alone ($\nu _d$ = 1 Hz; Fig. S3**b**). These findings suggest that mitochondria amplify astrocytic responses by transiently lowering cytosolic Ca^2+^ and/or sustaining higher IP_3_ concentrations.

**Fig. 4:**
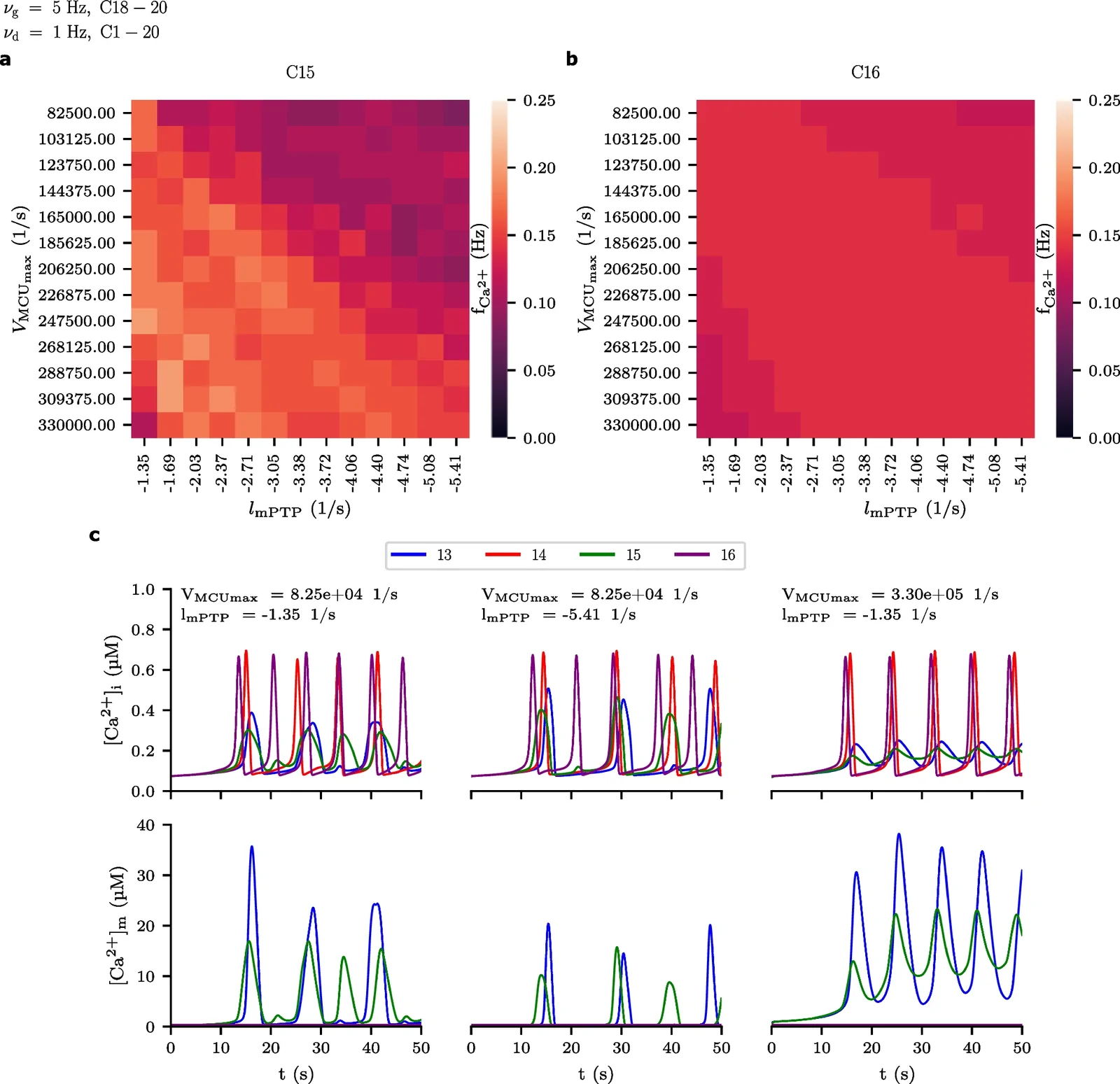
Mitochondrial Ca^2+^ uptake and release rates modulate the frequency and shape Ca^2+^ signals. $f_\mathrm {Ca^{2+}}$ in (**a**) compartment 15 and (**b**) compartment 16 for different combinations of maximum MCU uptake rate ($\mathrm {V_{MCU}}$) and mPTP release rate ($\mathrm {l_{mPTP}}$). **c** Representative cytosolic ($\mathrm {[Ca^{2+}]}_i$; upper panels) and mitochondrial ($\mathrm {[Ca^{2+}]}_m$; lower panels) traces for three combinations of MCU uptake rate and mPTP release rate. Compartments 15 and 13 have mitochondria

To assess the roles of mitochondrial uptake via the MCU and release via the mPTP, we varied the maximum MCU uptake rate ($V_\textrm{MCUmax}$, Eq. (3)) and mPTP release rate ($l_\textrm{mPTP}$, Eq. (4)). The effects on $f_\mathrm {Ca^{2+}}$ depended on their balance (Fig. 4**a**, Fig. S4**a**). With low mPTP release, increasing MCU uptake reduced oscillation frequency, whereas at higher release rates, greater MCU uptake increased $f_\mathrm {Ca^{2+}}$. Compartments without mitochondria were minimally affected (Fig. 4**b**, Fig. S4**a**). Overall, the highest $f_\mathrm {Ca^{2+}}$ occurred when MCU uptake and mPTP release were balanced (Fig. S4**b**).

Distinct patterns of Ca^2+^ signals emerged under different $V_\textrm{MCUmax}$–$l_\textrm{mPTP}$ combinations (Fig. 4**c**). Low MCU uptake and mPTP release produced small-amplitude cytosolic signals but high $f_\mathrm {Ca^{2+}}$, accompanied by larger mitochondrial signals. Increasing mPTP release while keeping MCU uptake constant raised cytosolic signal amplitude but lowered $f_\mathrm {Ca^{2+}}$, with mitochondrial signals showing isolated peaks. Conversely, high MCU uptake with low mPTP release stabilized cytosolic signals and induced sustained mitochondrial Ca^2+^ accumulation.

Together, these results show that both MCU-mediated uptake and mPTP-mediated release govern $f_\mathrm {Ca^{2+}}$. The highest oscillation frequencies occur when uptake and release are balanced, a condition associated with smaller cytosolic oscillation amplitudes. Moreover, different $V_\textrm{MCUmax}$–$l_\textrm{mPTP}$ regimes generate distinct cytosolic and mitochondrial Ca^2+^ oscillatory patterns.

#### IP_3_-Ca^2+^ interaction

As shown above, higher $f_{Ca^{2+}}$ in the presence of mitochondria is associated with smaller cytosolic Ca^2+^ oscillation amplitudes and elevated cytosolic IP_3_. To examine how mitochondria enhance the Ca^2+^ response, we varied the parameters controlling Ca^2+^–IP_3_ interactions: the Ca^2+^-dependent IP_3_ degradation constant ($K_D$, Eq. S23), the Ca^2+^-dependent inhibition of PLC ($K_p$, Eq. S21), the Ca^2+^ affinity of PLC ($K_\pi$, Eq. S21), and the Ca^2+^-independent IP_3_ degradation rate ($r_{5P}$, Eq. S24). We then compared the difference in $f_\mathrm {Ca^{2+}}$ between models with and without mitochondria (zero indicating similar responses). The unipolar morphology was stimulated with glutamate ($\nu _g$ = 5 Hz, C18–20) and dopamine ($\nu _d$ = 1 Hz, C1–20). In addition to $f_\mathrm {Ca^{2+}}$, we analyzed the time-averaged Ca^2+^ efflux through IP_3_ receptors ($J_{IP_3R}$, Eq. S12, Fig. S6) and the average IP_3_ degradation by IP_3_-3K ($D_{\mathrm {IP_3-3K}}$, Eq. S23, Fig. S7). In the main text, we report results for $K_D$, while the others are presented in Online Resource 1.

**Fig. 5:**
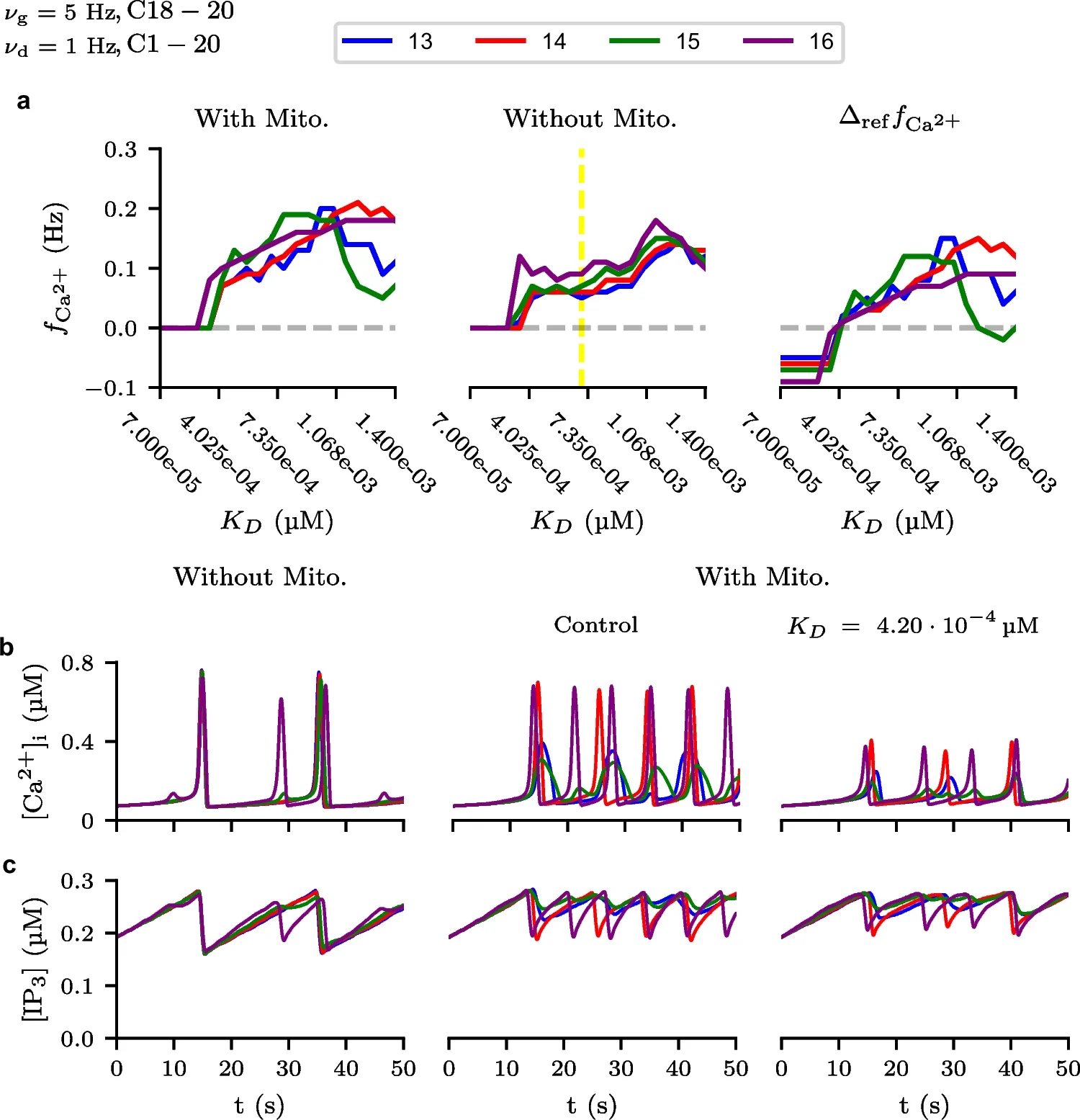
Mitochondria reduce Ca^2+^-dependent IP_3_ degradation. $K_D$ controls the Ca^2+^ affinity of IP_3_-3K. (**a**) Frequency of Ca^2+^ signals ($f_\mathrm {Ca^{2+}}$) as a function of $K_D$. The vertical yellow dashed line indicates the reference value $K_D^{\textrm{ref}}=0.7$ µM used in the model without mitochondria. The corresponding $f_\mathrm {Ca^{2+}}$ defines the fixed reference used to calculate $\Delta _{\textrm{ref}} f_\mathrm {Ca^{2+}} (K_D)$; positive and negative values indicate responses above and below this reference, respectively. (**b**) Cytosolic Ca^2+^ concentrations, and (**c**) cytosolic IP_3_ concentrations for the model without mitochondria, the model with mitochondria at the default $K_D$, and the model with mitochondria at reduced $K_D$

Increasing Ca^2+^-dependent IP_3_ degradation shifted $f_{Ca^{2+}}$ in the mitochondrial model toward the fixed non-mitochondrial reference, thereby reducing $\Delta _{\textrm{ref}}f_\mathrm {Ca^{2+}}$ (Fig. 5**a**, Fig. S5). Lowering $K_D$ decreased ER Ca^2+^ release and enhanced IP_3_ degradation (Fig. S6, S7), thereby reducing $f_{Ca^{2+}}$ in both models (Fig. 5**a**). In contrast, varying $K_p$ or $K_\pi$ had no measurable effect on $f_{Ca^{2+}}$, $J_{IP_3}$, or $D_{\mathrm {IP_3-3K}}$ (Figs. S5–S7). Increasing $r_{5P}$, like decreasing $K_D$, lowered $f_{Ca^{2+}}$ (Fig. S5) and ER Ca^2+^ efflux (Fig. S6), though without major changes in Ca^2+^-dependent degradation (Fig. S7). Thus, enhanced IP_3_ degradation shifts the mitochondrial response toward the non-mitochondrial reference.

Reducing $K_D$ decreased $f_{Ca^{2+}}$ in the mitochondrial model to values comparable to the model without mitochondria, but also lowered Ca^2+^ signal amplitudes in all compartments (Fig. 5**b**). Similarly, IP_3_ concentrations were reduced in the mitochondrial model with lower $K_D$ (Fig. 5**c**). However, the IP_3_ dynamics were not fully matched: oscillation amplitudes remained different between models. Thus, while stronger Ca^2+^-dependent IP_3_ degradation makes the mitochondrial and non-mitochondrial responses more similar, Ca^2+^ and IP_3_ amplitudes remain distinct.

To further test whether mitochondria enhance astrocytic responses by limiting Ca^2+^-dependent IP_3_ degradation, we simulated reduced MCU uptake combined with lower IP_3_ degradation. Glutamatergic ($\nu _g$ = 5 Hz, C18–20) and dopaminergic ($\nu _d$ = 1 Hz, C1–20) inputs were applied. Here we show results for $V_{\textrm{MCUmax}} = 41.25 \cdot 10^3$ s$^{-1}$ and $K_D = 0.84$ µM; full results are provided in Online Resource 1 (Fig. S8).

**Fig. 6:**
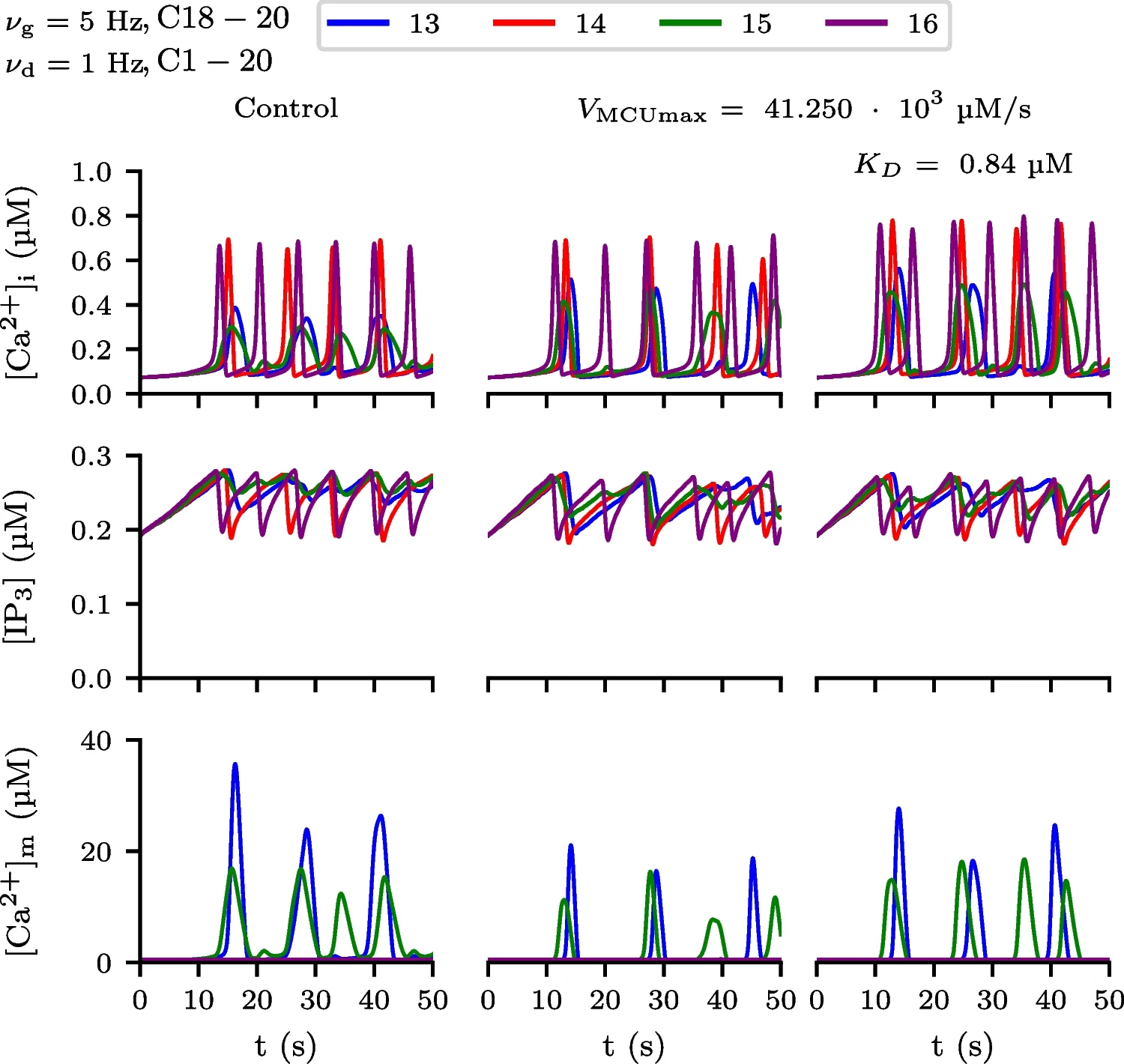
Reducing IP_3_ degradation counteracts the effects of reduced MCU Ca^2+^ uptake on cytosolic signals. Column 1 shows the traces for the control condition ($V_\textrm{MCUmax} = 165 \times 10^3$ s^-1^; $K_D = 0.7$ µM). Columns 2 and 3 show traces with $V_\textrm{MCUmax} = 41.25 \times 10^3$ s^-1^; column 3 also has $K_D = 0.84$ µM. Distal compartments were stimulated with glutamate ($\nu _g$ = 5 Hz), all compartments with dopamine ($\nu _d$ = 1 Hz)

MCU inhibition reduced both cytosolic and mitochondrial $f_\mathrm {Ca^{2+}}$, increased cytosolic oscillation amplitude, and induced isolated mitochondrial Ca^2+^ peaks (Fig. 6). Reducing Ca^2+^-dependent IP_3_ degradation (by increasing $K_D$) restored cytosolic $f_\mathrm {Ca^{2+}}$ to control levels despite MCU inhibition, while further amplifying cytosolic Ca^2+^ oscillation amplitudes. Mitochondrial oscillation amplitudes also increased under reduced IP_3_ degradation, though they remained lower than control and still exhibited isolated peaks. Thus, the impact of MCU inhibition on both cytosolic and mitochondrial responses was partially offset by weaker Ca^2+^-dependent IP_3_ degradation.

In summary, these results suggest that mitochondria enhance astrocytic Ca^2+^ signals by transiently reducing cytosolic Ca^2+^, thereby lowering Ca^2+^-dependent IP_3_ degradation. This allows IP_3_ to accumulate and sustain IP_3_R activation, increasing Ca^2+^ signals. Although mitochondria are localized to specific compartments, their influence propagates throughout the cell.

#### Mitochondria enhance the propagation of Ca^2+^ signals

As shown above, mitochondria enhance the Ca^2+^ response to glutamate and dopamine by limiting IP_3_ degradation. In previous work, we demonstrated that dopamine also boosts glutamate-triggered responses and increases the extent of propagation of Ca^2+^ signals from distal compartments, thereby improving communication between branchlets in astrocyte models (Bezerra & Roque, 2024). To test whether mitochondria similarly influence Ca^2+^ propagation, we simulated the unipolar astrocyte model under varying dopaminergic ($\nu _d$ = 0, 0.005, 0.01, 0.05 Hz; C1–20) and glutamatergic ($\nu _g$ = 0–10 Hz; C18–20) inputs. The extent of propagation (also, “propagation extent”) of a Ca^2+^ signal was measured as the number of compartments activated, counting from the most distal one (see Online Resource 1 for stimulation details).

**Fig. 7:**
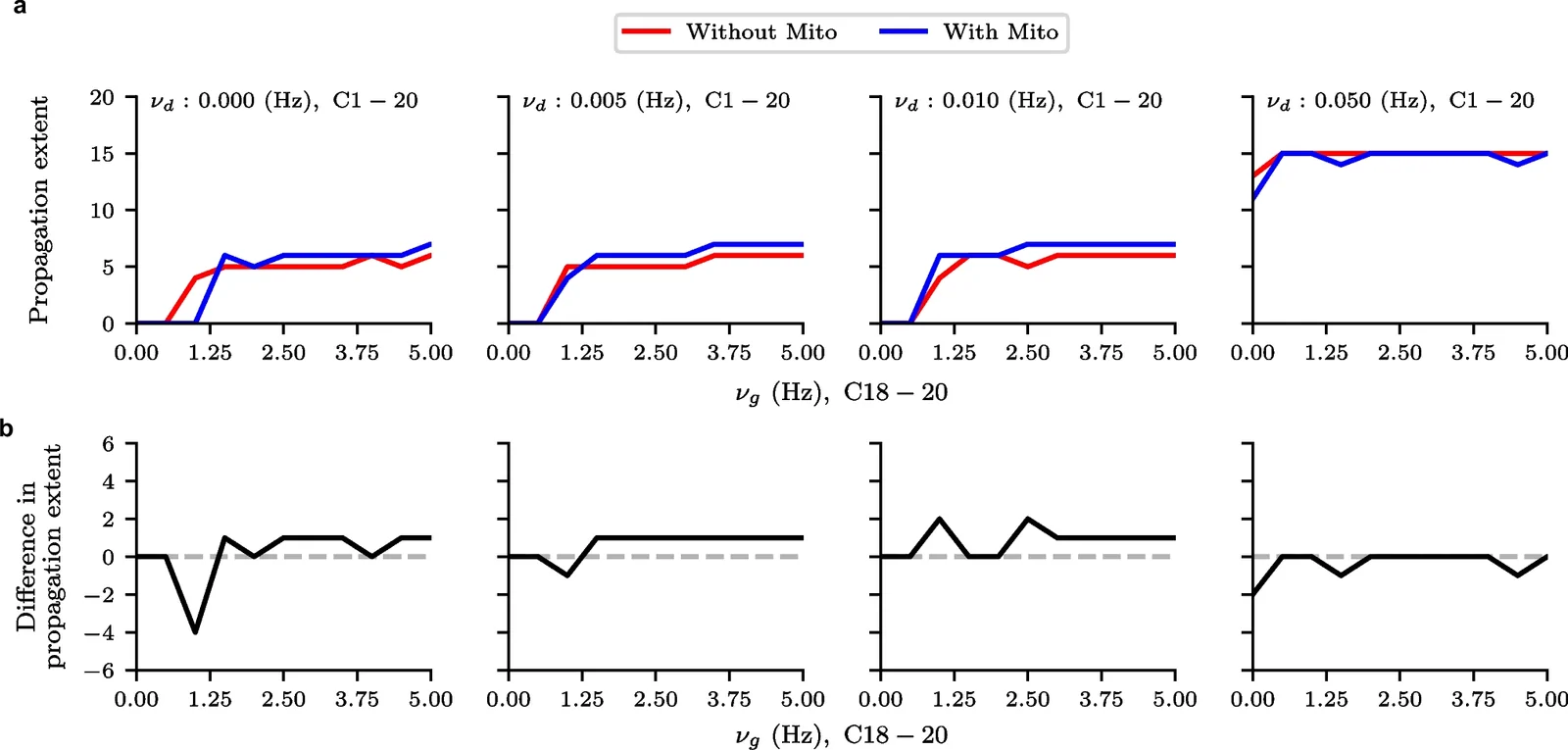
Mitochondria gate Ca^2+^ signal transmission in a linear astrocytic branch. Distal compartments received glutamatergic input ($\nu _g$ = 0–5 Hz) while the whole cell received dopaminergic input ($\nu _d$ = 0, 0.005, 0.01, 0.05 Hz). (**a**) Propagation extent. (**b**) Pointwise difference in propagation extent between models with and without mitochondria, calculated for each combination of $\nu _g$ and $\nu _d$ as the value in the model with mitochondria minus that in the model without mitochondria

Without dopamine, mitochondria exerted a bimodal effect on glutamate-driven Ca^2+^ propagation. At low glutamatergic frequencies ($\nu _g$ *¡ * 1.5 Hz), the propagation extent was reduced, whereas at higher frequencies it increased (Fig. 7**a**). For example, at $\nu _g$ = 1 Hz, the propagation extent was shortened by four compartments in the mitochondrial model (Fig. 7**b**), while for $\nu _g \ge 1.5$ Hz the propagation extent increased by up to one compartment. Low-frequency dopamine ($\nu _d$ = 0.005 Hz) attenuated the mitochondrial reduction in the extent of propagation (Fig. 7**b**), and at $\nu _d$ = 0.01 Hz mitochondria enhanced the propagation extent for all glutamatergic frequencies. At the strongest dopamine level ($\nu _d$ = 0.05 Hz), mitochondrial effects were minimal, since dopaminergic input alone already triggered near-global Ca^2+^ activation. Thus, mitochondria gate signal transmission from distal compartments: under weak glutamatergic input and no dopamine, they restrict propagation, preventing weak stimuli from activating large astrocytic regions. Dopamine lowers this threshold, enabling weaker inputs to propagate more effectively. This can be seen as a leftward shift in the propagation extent curve (Fig. 7**a**).

Because cytosolic Ca^2+^ also regulates mitochondrial metabolism (Filadi & Greotti, 2021), and SERCA pumps are ATP-dependent, mitochondria might affect propagation by altering SERCA-mediated uptake. As a control, we varied the maximum SERCA rate ($v_\textrm{ER}$, Eq. S14), thereby indirectly simulating energy metabolism. However, higher SERCA activity reduced the extent of propagation (Fig. S8), opposite to the mitochondrial effect (Fig. 7**a**).

To further probe mitochondrial gating, we tested a unipolar morphology with a single mitochondrion placed in different compartments and applied localized glutamatergic stimulation (Fig. S9). The extent of propagation was again defined as the number of compartments activated. Mitochondria near distal compartments suppressed propagation of locally triggered Ca^2+^ signals, whereas mitochondria placed at the distal–intermediate transition (C15–17) increased the propagation extent. Along the main branch, mitochondria only blocked propagation when located near the stimulation site. Stimuli applied to somatic or proximal compartments never propagated, regardless of mitochondrial position (Fig. S9).

**Fig. 8:**
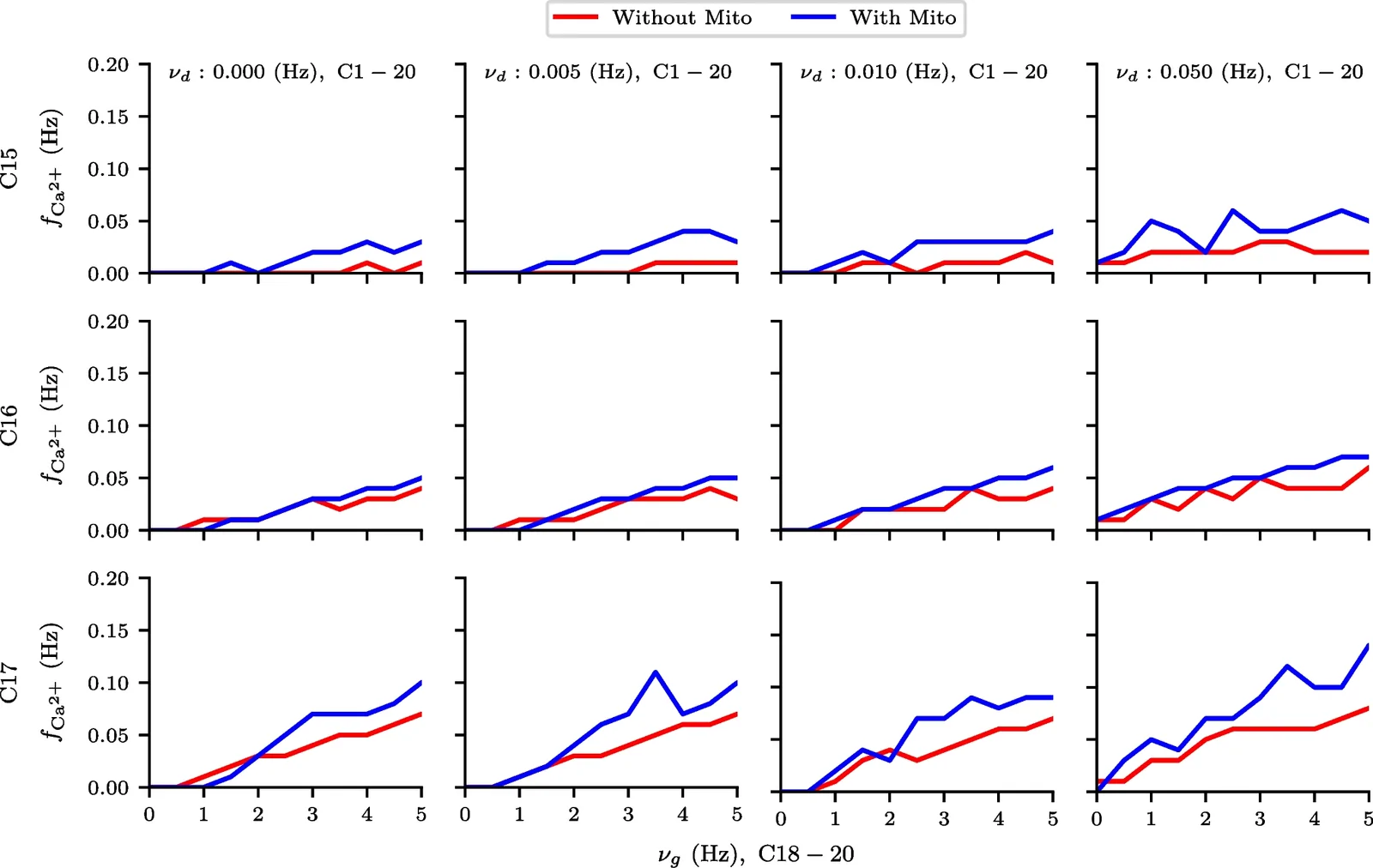
Mitochondria regulate $f_\mathrm {Ca^{2+}}$ in the linear astrocytic branch. Distal compartments received glutamate ($\nu _g$ = 0–5 Hz), while the whole cell received dopamine ($\nu _d$ = 0, 0.005, 0.01, 0.05 Hz). From top to bottom: results for C15, C16 and C17

A similar bimodal effect was observed for $f_\mathrm {Ca^{2+}}$ (Fig. 8). We focused on compartments C15–17 (full results in Fig. S11). Compartments C15 and C17 contained mitochondria. In C16 and C17, mitochondria reduced $f_{Ca^{2+}}$ at weak glutamatergic input ($\nu _g \le 2$ Hz) without dopamine, but increased it at higher $\nu _g$ (Fig. 8). By contrast, in C15 mitochondria enhanced responses for all $\nu _g$. Dopamine shifted the $f_{Ca^{2+}}$ curves leftward, lowering the glutamatergic threshold for mitochondrial enhancement. At the highest dopamine level ($\nu _d$ = 0.05 Hz), the mitochondrial model showed consistently higher $f_{Ca^{2+}}$ for all $\nu _g$. In both models, increasing $\nu _g$ raised $f_{Ca^{2+}}$ across all compartments. In the single-mitochondrion simulations, $f_\mathrm {Ca^{2+}}$ was also position-dependent: co-localization of mitochondria and input in C17–18 enhanced responses, whereas mitochondria near distal ends reduced responses in C19–20 (Fig. S12).

These results suggest that mitochondria exert a bimodal influence depending on glutamatergic strength and dopaminergic input. Under weak glutamatergic stimulation, they gate transmission from distal compartments, reducing the extent of propagation and $f_\mathrm {Ca^{2+}}$. With stronger glutamatergic input and/or dopaminergic modulation, mitochondria enhance both the spread of Ca^2+^ signals and their oscillation frequency.

#### Mitochondria gate the transmission of Ca^2+^ from terminal regions

**Fig. 9:**
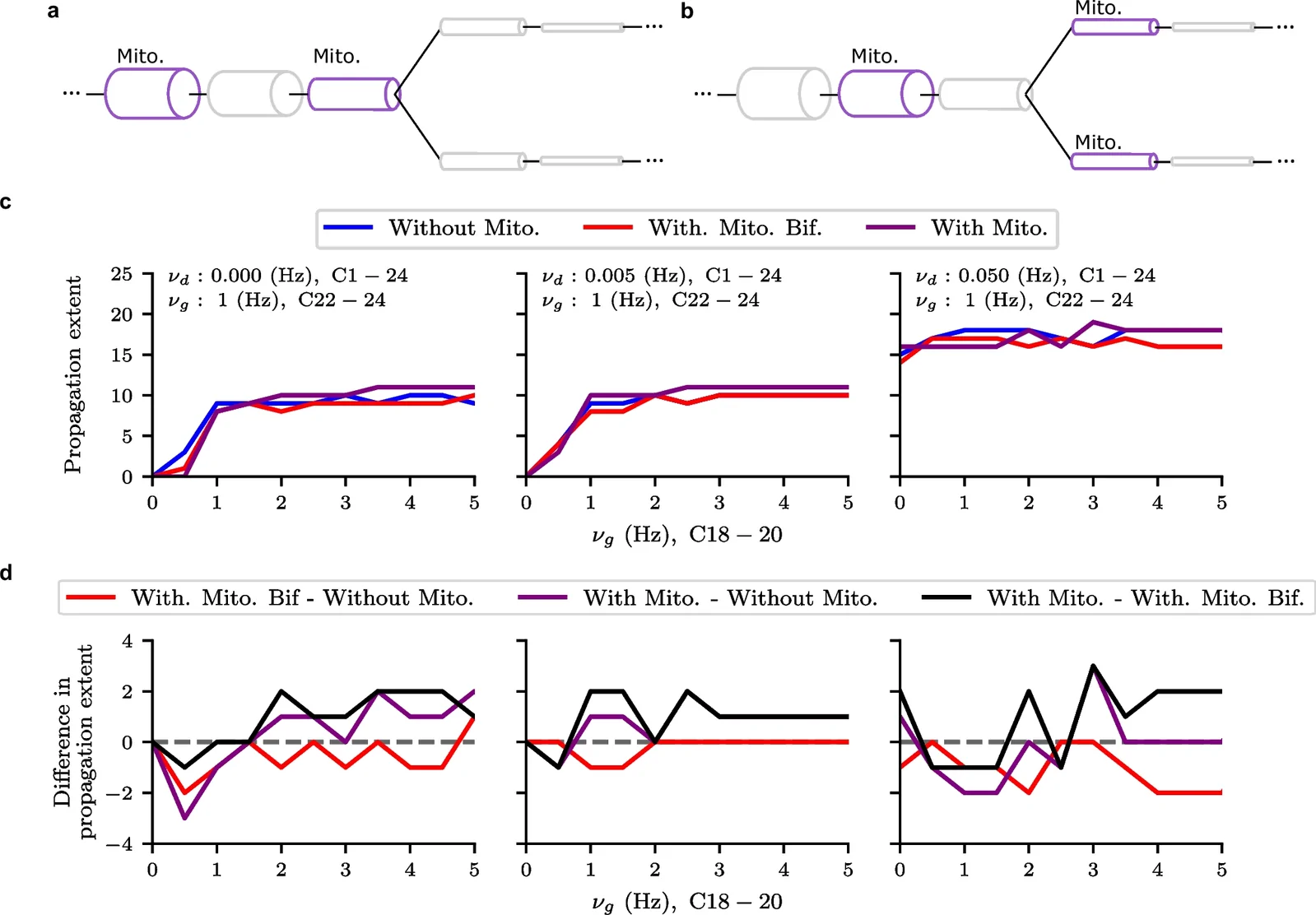
Mitochondria gate Ca^2+^ transmission from secondary branchlets. One branchlet received glutamate at fixed frequency ($\nu _g =$ 1 Hz), while the other was stimulated with varying glutamatergic input ($\nu _g =$ 0–5 Hz). Dopamine was applied to the whole astrocyte ($\nu _d =$ 0, 0.005, or 0.05 Hz). (**a**) Morphology with mitochondria at the bifurcation. (**b**) Morphology without mitochondria at the bifurcation. (**c**) Propagation extent. (**d**) Pointwise difference in propagation extent between models with and without mitochondria at the bifurcation, evaluated for the same stimulation condition

Since the hierarchical branching of astrocytic processes influences how they interact (Bezerra & Roque, 2024), and because we found that mitochondria gate the propagation of signals from distal regions, we next tested whether mitochondria could affect the interaction between secondary branchlets. For this, we used a bifurcated terminal morphology consisting of a single process emerging from the soma and bifurcating near the terminal region (Fig. 9**a**, **b**; Tables S3 and S4 in Online Resource 2). We compared two conditions: one with mitochondria at the bifurcation point (“With Mito. Bif.”; Fig. 9**a**) and another without mitochondria at this location (“With Mito.”; Fig. 9**b**). In each trial, one branchlet received glutamatergic stimulation at varying frequencies ($\nu _g =$ 0–5 Hz), while the other branchlet received weak glutamatergic input ($\nu _g =$ 1 Hz). The entire cell was also stimulated with dopamine at different frequencies ($\nu _d =$ 0, 0.005, 0.05, or 0.5 Hz). Details of the stimulation protocol are provided in Online Resource 1.

In the absence of dopamine ($\nu _d =$ 0) and under low glutamatergic input ($\nu _g<$ 1.5 Hz), mitochondria reduced the propagation of Ca^2+^ signals compared to the model without mitochondria (Fig. 9**c**). For any $\nu _g$, mitochondria located at the bifurcation point further restricted propagation relative to models without mitochondria at that site (Fig. 9**c**). Overall, comparing all model variants, mitochondria at the bifurcation consistently reduced propagation distance (Fig. 9**d**). By contrast, when mitochondria were absent from the bifurcation, the effect resembled that of the unipolar morphology, where mitochondria enhanced responses (Fig. 7). With low-frequency dopamine ($\nu _d =$ 0.005 Hz), the model with mitochondria at the bifurcation still showed reduced propagation compared to both alternatives (Fig. 9**c**, **d**). Even at higher dopamine levels, the propagation extent remained lower when mitochondria were present at the bifurcation, compared to models without them (Fig. 9**c**, **d**).

**Fig. 10:**
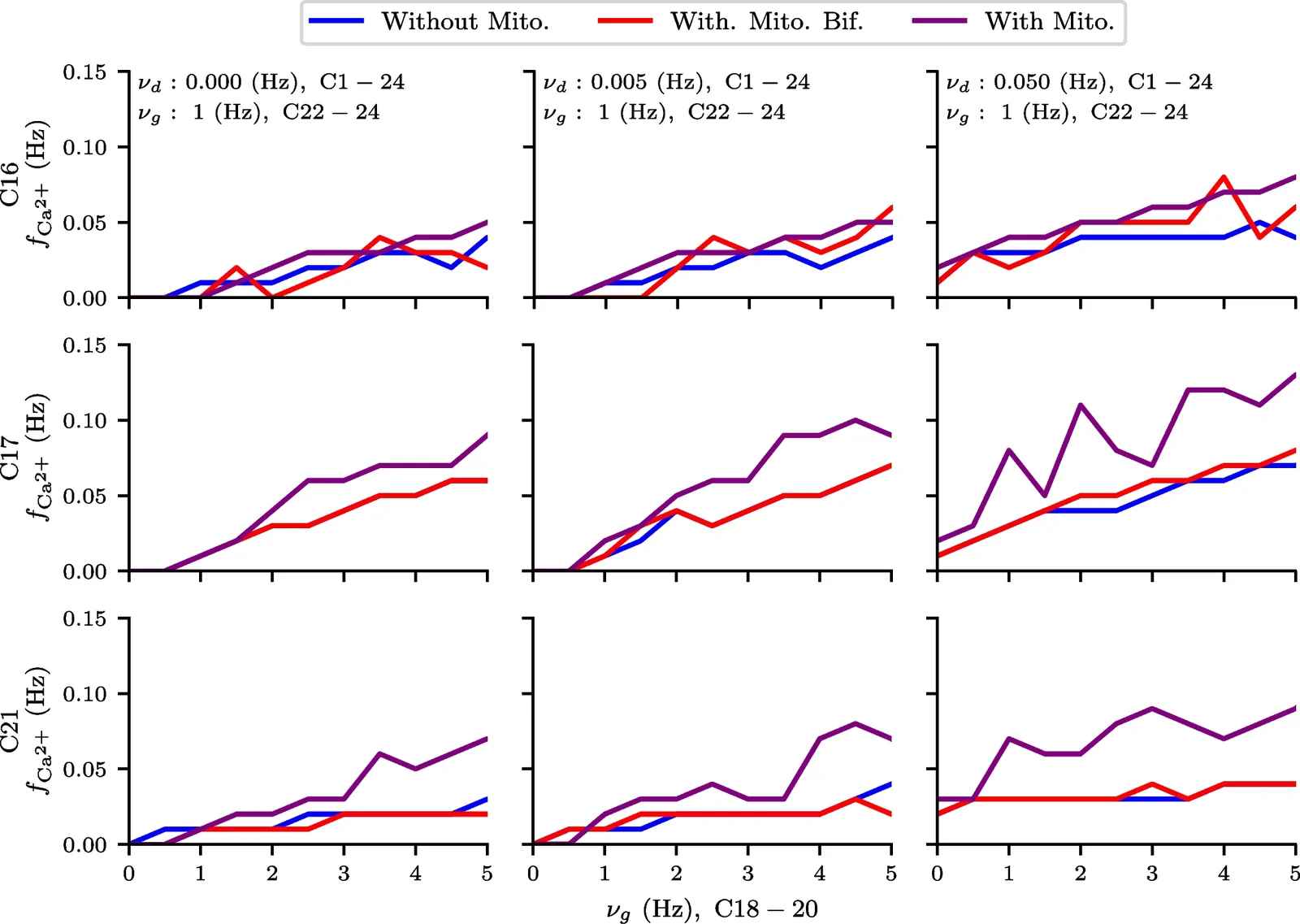
Mitochondria at the bifurcation gate Ca^2+^ signals in secondary branchlets. All compartments received dopamine ($\nu _d =$ 0, 0.005, or 0.05 Hz). The second branchlet was stimulated with glutamate ($\nu _g =$ 1 Hz), while the first branchlet received varying glutamatergic input ($\nu _g =$ 0–5 Hz). From top to bottom: results for C16, C17 and C21

A similar pattern was observed for $f_\mathrm {Ca^{2+}}$. We focused on compartments C16 (the bifurcation point), C17, and C21 (the adjacent compartments). Without dopamine, the responses of C17 and C21 in the bifurcation-mitochondria model resembled those of the no-mitochondria model (Fig. 10). In C16, $f_\mathrm {Ca^{2+}}$ was higher in the bifurcation-mitochondria model than in the no-mitochondria model for strong glutamatergic input ($\nu _g>$ 3 Hz). With dopamine, $f_\mathrm {Ca^{2+}}$ in C16, C17, and C21 was slightly higher than in the no-mitochondria model (Fig. 10). Dopamine shifted the $f_\mathrm {Ca^{2+}}$ curves leftward, reducing the glutamatergic threshold for enhanced responses in both models with mitochondria. However, in all cases, the model without mitochondria at the bifurcation exhibited higher $f_\mathrm {Ca^{2+}}$ than the model with mitochondria at the bifurcation, again resembling the unipolar morphology (Figs. 8 and 10).

Together, these results indicate that mitochondria positioned at the bifurcation point gate Ca^2+^ transmission between secondary branchlets and prevent their communication, even under strong synaptic input in one branchlet. This contrasts with the unipolar morphology and with our previous results using a similar bifurcated terminal morphology (Bezerra & Roque, 2024).

## Discussion

### Main findings

Here we present a computational astrocyte model incorporating MCU-mediated mitochondrial Ca^2+^ uptake and mPTP-mediated release. The simulations show that mitochondria modulate astrocytic Ca^2+^ signaling in a context-dependent manner. Under weak glutamatergic input ($\nu _g<$ 2 Hz) in the absence of dopamine, mitochondria generally suppress Ca^2+^ responses and restrict their propagation. In contrast, stronger glutamatergic stimulation ($\nu _g \ge$ 2 Hz) and/or dopaminergic input shift the mitochondrial effect toward enhancement, increasing Ca^2+^ signal frequency and propagation. This context dependence arises because mitochondrial Ca^2+^ uptake reduces cytosolic Ca^2+^ and thereby limits Ca^2+^-dependent IP_3_ degradation, allowing IP_3_ to remain elevated and facilitating subsequent ER Ca^2+^ release.

Mitochondrial Ca^2+^ uptake can be viewed initially as a local buffering mechanism: MCU-mediated uptake reduces free cytosolic Ca^2+^ and can therefore attenuate weak Ca^2+^ transients. In the present model, however, this buffering has an additional indirect effect because IP_3_ degradation by IP_3_-3K is Ca^2+^ dependent. By transiently lowering cytosolic Ca^2+^, mitochondria reduce IP_3_ degradation, prolonging IP_3_ availability and facilitating subsequent IP_3_R-mediated Ca^2+^ release. Mitochondrial buffering can therefore suppress weak responses while promoting sustained or propagating Ca^2+^ signaling under stronger stimulation. More generally, the location and capacity of intracellular Ca^2+^ buffers can influence the amplitude, duration, and spatial spread of astrocytic Ca^2+^ signals, making mitochondrial positioning potentially important for local signal integration.

These effects extend the role of mitochondria beyond passive Ca^2+^ buffering. By altering Ca^2+^-dependent IP_3_ degradation locally, mitochondria influence whether a Ca^2+^ signal remains confined to its site of origin or propagates to neighboring compartments. Their position is therefore important: mitochondria located at branch points can restrict communication between branchlets even when mitochondria elsewhere facilitate propagation. In this sense, mitochondria behave as context-dependent spatial gates of intracellular Ca^2+^ information flow.

Remarkably, as mitochondria are motile organelles and can accumulate near regions of elevated Ca^2+^ activity or glutamate uptake, activity-dependent mitochondrial repositioning could dynamically alter these spatial gating properties by changing local Ca^2+^ buffering capacity (Cabral-Costa & Kowaltowski, 2023; Jackson & Robinson, 2015).

Overall, these findings suggest that mitochondrial Ca^2+^ handling may contribute to the selective spatial integration of neuronal inputs by astrocytes: weak local inputs are more likely to remain spatially restricted, whereas stronger synaptic activity or neuromodulatory input can favor broader Ca^2+^ responses. Astrocytes have been implicated in coding brain states, such as arousal, via neuromodulatory inputs (Rasmussen et al., 2023; Reitman et al., 2023). Because neuromodulators act through volume transmission (Bezerra & Roque, 2024; Liu et al., 2021; Rasmussen et al., 2023), they are well suited to evoke global astrocytic signals (Rasmussen et al., 2023). In this framework, global astrocytic signals may underlie state coding, with mitochondria tuning astrocytic responsiveness to task and network demands. Supporting this idea, dopaminergic neuron activity in the *substantia nigra* correlates with cognitive task demand (Boehler et al., 2011). By confining responses under weak inputs, mitochondria ensure local modulation, while under strong activity or neuromodulator release, they facilitate global astrocytic activation, enabling broad network modulation and synchronization (Lee et al., 2014).

Ca^2+^ is also a key regulator of mitochondrial metabolism, enhancing ATP synthesis (Filadi & Greotti, 2021). Several Ca^2+^ regulators, including SERCA, PMCA, and NCX, are energy-dependent, linking Ca^2+^ handling to metabolic demand. Thus, Ca^2+^ signals may encode network energy requirements. High neural activity, by increasing the frequency of Ca^2+^ signals, would drive higher mitochondrial metabolism. By gating propagation from terminal regions, mitochondria may prevent unnecessary metabolic activation under weak stimuli, while during high-demand states, Ca^2+^ responses signal energy needs that are amplified by mitochondria. On the other hand, since Ca^2+^ signaling in astrocytes is associated with gliotransmitter release, modulation of synaptic transmission, and regulation of neurovascular coupling (Covelo & Araque, 2018; De Ceglia et al., 2023; Goenaga et al., 2023; Lind et al., 2018; Tesler et al., 2023), Ca^2+^ signaling links cellular energy metabolism to distinct brain functions.

Taken together, our simulation results suggest that interactions among mitochondrial Ca^2+^ handling, local glutamatergic input, and global dopaminergic modulation may tune astrocytic responses according to task and network demands.

### Comparison with previous models and experiments

Previous computational studies incorporating mitochondrial Ca^2+^ dynamics in astrocytes have generally included mitochondria as components of broader Ca^2+^ or metabolic models rather than examining how mitochondrial Ca^2+^ handling modifies neurotransmitter-evoked astrocytic signaling. Komin et al. (2015) incorporated mitochondrial Ca^2+^ release in a stochastic astrocytic Ca^2+^ model, whereas Diekman et al. (2013) modeled MCU-mediated uptake and NCX-mediated efflux in the context of mitochondrial metabolism and neuroprotection. The present model differs in focusing specifically on how mitochondrial Ca^2+^ uptake and release interact with IP_3_ dynamics, neurotransmitter input, and astrocytic morphology to regulate signal frequency and spatial propagation.

The present computational model extends previous studies by including the MCU and mPTP, mitochondrial mechanisms that regulate intracellular Ca^2+^ (Agarwal et al., 2017; Huntington & Srinivasan, 2021; Serrat et al., 2021), and directly studying how these mechanisms modulate Ca^2+^ signaling in astrocytes (De Young & Keizer, 1992; Höfer et al., 2002; Li & Rinzel, 1994; Manninen et al., 2018; Oschmann et al., 2017). In our model, mitochondria lowered the amplitude of Ca^2+^ signals in compartments containing this organelle but, under strong glutamatergic or dopaminergic input, increased the oscillation frequency throughout the cell. Mechanistically, mitochondria transiently reduce cytosolic Ca^2+^, thereby limiting Ca^2+^-dependent IP_3_ degradation by IP_3_-3K. As proposed by previous computational studies, stimulation sites can be viewed as “sources” of IP_3_ and non-stimulated compartments as “sinks” (Lallouette et al., 2014, 2019; Matrosov et al., 2019). By suppressing IP_3_ degradation, mitochondria reduce sink effects, facilitating IP_3_ accumulation and signal propagation. With dopamine, all compartments effectively become IP_3_ sources, reducing mitochondrial impact. Conversely, under weak glutamatergic input, mitochondria already sequester the limited Ca^2+^ released, thereby preventing responses. Thus, the model predicts that Ca^2+^ signals near mitochondria should have lower amplitudes and that larger, propagating events require strong synaptic input or neuromodulator release (e.g., dopamine, noradrenaline). This process should be accompanied by higher IP_3_ concentration close to the mitochondria. Blocking mitochondrial Ca^2+^ handling should reduce propagation and event size under strong stimulation, but enhance responses under weak glutamatergic input.

Comparison with available experimental data indicates that several temporal features of the simulated cytosolic Ca^2+^ responses are compatible with reported observations. Signal frequencies and durations generally fall within experimentally reported ranges, and some qualitative effects of perturbing mitochondrial Ca^2+^ handling are reproduced. However, not all effects agree with available data; in particular, the increase in cytosolic Ca^2+^ amplitude predicted after reducing MCU uptake differs from studies reporting little or no amplitude change after MCU inhibition. Detailed comparisons are provided in Online Resource 1.

Direct quantitative comparison is more limited for mitochondrial Ca^2+^ dynamics and spatial propagation. Experimental data obtained under conditions directly corresponding to the simulated stimulation protocols are not available for quantitatively constraining the amplitude and time course of mitochondrial Ca^2+^ transients, and the compartment-based measure of propagation extent has no direct experimental counterpart. The predicted context-dependent gating of Ca^2+^ propagation should therefore be regarded as a testable model prediction rather than an established physiological mechanism. Simultaneous imaging of cytosolic and mitochondrial Ca^2+^ during spatially localized glutamatergic stimulation, with and without neuromodulatory input, could directly test this prediction.

The interaction between dopaminergic input and mitochondrial regulation of astrocytic Ca^2+^ signaling may also be relevant to conditions in which dopaminergic transmission and mitochondrial function are altered, including Parkinson’s disease and schizophrenia. The present model does not represent a disease state, but it provides a framework for testing how changes in dopaminergic drive or mitochondrial Ca^2+^ handling could alter astrocytic integration of local synaptic and global neuromodulatory inputs.

### Model limitations

It is important to consider the limitations of the present model when interpreting the results and conclusions. The mitochondrial Na^+^/Ca^2+^ exchanger (NCLX) represents an additional pathway for mitochondrial Ca^2+^ efflux that was not included in the present model (Cabral-Costa & Kowaltowski, 2023; Huntington & Srinivasan, 2021; Parnis et al., 2013). A mechanistic implementation of NCLX would require the inclusion of additional ionic species and mitochondrial membrane potential, thereby substantially increasing model complexity. Moreover, Agarwal et al. (2017) did not observe a detectable effect of NCLX inhibition on the astrocytic Ca^2+^ dynamics examined in their *in vivo* experiments. We therefore restricted this first model to MCU- and mPTP-mediated mitochondrial Ca^2+^ handling. Other factors that modulate MCU and mPTP function, including ionic composition, redox state, and mitochondrial membrane potential, were also not explicitly represented (Dong et al., 2017; Filadi & Greotti, 2021; Pradhan et al., 2010). Thus, MCU and mPTP should be interpreted here as effective Ca^2+^-handling mechanisms rather than as a complete biophysical representation of mitochondrial ion transport.

In addition, mitochondrial contributions to Ca^2+^ regulation may vary across brain regions and among subcellular compartments, reflecting differences in mitochondrial function, morphology, and intracellular distribution (Derouiche et al., 2015; Faitg et al., 2021; Huntington & Srinivasan, 2021; Jackson & Robinson, 2015; Oliveira & Gonçalves, 2009). For example,  Huntington and Srinivasan (2021) found no detectable contribution of MCU to the astrocytic activity examined in mouse striatum, illustrating that the relative importance of individual mitochondrial mechanisms may be region dependent.

The present simulations also assume a particular mitochondrial size and distribution. Experimental studies have described mitochondrial complexes extending along larger astrocytic processes (Salmon et al., 2023), whereas our standard model places mitochondria in alternating compartments. Nevertheless, varying mitochondrial number and distribution did not alter the main qualitative conclusions (Figs. S14 and S16), although these properties affected quantitative aspects of the Ca^2+^ response.

Similarly, glutamatergic inputs were placed predominantly in distal compartments to examine propagation from fine processes toward the soma. Synaptic contacts occur throughout the astrocytic arbor and have been reported at distances of approximately 2-10 µm from mitochondria (Salmon et al., 2023; Stephen et al., 2015). In the present model, the nearest mitochondria are approximately 1-4 µm from the stimulated compartments, within this experimentally reported range. Future studies using more realistic morphologies and distributions of synaptic contacts will be needed to determine how these spatial relationships influence mitochondrial gating.

A further limitation concerns the temporal waveform of individual Ca^2+^ signals. Experimental astrocytic Ca^2+^ transients often show a rapid rise followed by a slower decay, whereas some signals generated by the present model exhibit a different temporal asymmetry. These kinetics arise from the IP_3_R, SERCA, and IP_3_ dynamics inherited from the underlying astrocytic Ca^2+^ formulation and were not independently fitted in this study. The present analyses therefore focus on comparative effects of mitochondrial mechanisms on signal frequency, amplitude, and propagation rather than on quantitative reproduction of the detailed waveform of individual Ca^2+^ transients.

Finally, mitochondria–ER contact sites (MERCS) (Cabral-Costa & Kowaltowski, 2023; Salmon et al., 2023; Serrat et al., 2021) were not explicitly represented in our standard model. A supplementary simulation in which part of the ER Ca^2+^ flux was directed to mitochondria preserved the frequency and spatial location of the astrocytic response but altered signal timing (Fig. S15), suggesting that explicit representation of MERCS may be important for reproducing temporal aspects of mitochondrial–ER Ca^2+^ coupling. These extensions provide natural directions for future development of the model.

## Conclusion

Our results provide computational evidence that mitochondrial Ca^2+^ handling can shape astrocytic Ca^2+^ signaling through its interaction with Ca^2+^-dependent IP_3_ degradation. This regulation is context dependent: mitochondria suppress signaling under weak synaptic stimulation but can enhance signal frequency and propagation during stronger synaptic activity or neuromodulatory input. Their spatial distribution may therefore allow mitochondria to act as local gates of Ca^2+^ information flow within astrocytic processes. The model provides a framework for investigating how neuronal activity, astrocytic Ca^2+^ signaling, and mitochondrial regulation interact, and can be extended to examine regional and subcellular heterogeneity in mitochondrial properties.

## Supplementary information

**Online Resource 1.** Supplementary text with model description, stimulation protocols, limitations, morphology, parameters, and computational and numerical methods.

**Online Resource 2.** Supplementary text with morphological models.

**Online Resource 3.** Supplementary text with all model parameters.

## Supplementary Information

Below is the link to the electronic supplementary material.

Supplementary file 1 (pdf 8724 KB)

Supplementary file 2 (pdf 122 KB)

Supplementary file 3 (pdf 171 KB)

## Data Availability

No datasets were generated or analysed during the current study.

## Code Availability

The model code is available on GitHub at: https://github.com/ThiagoTakechi/AstrocyteMitochondriaModel.git.
