# Supplementary material for: Context-Dependent modulation of astrocytic Ca2+ signals by mitochondria: a computational study
Source: J Comput Neurosci. 2026 Sep 25;54(4):743–62. doi: 10.1007/s10827-026-00956-3 (PMC13710611; doi:10.1007/s10827-026-00956-3)
Supplement: Supplementary file 1 [file 10827_2026_956_MOESM1_ESM.pdf]

Context-Dependent Modulation of Astrocytic Ca^{2+} Signals by Mitochondria - A Computational Study
Online Resource 1: Supporting Information

Thiago Ohno Bezerra[1], Antonio C. Roque[1,*]

[1] Department of Physics, School of Philosophy, Sciences and Letters of Ribeirão Preto, University of São Paulo, Bandeirantes avenue, Ribeirão Preto, 14040-900, São Paulo, Brazil
* Corresponding author. E-mail: antonior@usp.br;

1 Detailed Model Description

Here we provide a detailed description of the variables and mechanisms included in the model. We first present the additional state variables and current densities, followed by the mechanisms of IP_3 synthesis and degradation and the rules governing coupling between compartments. We then derive the geometrical relation between cytosolic and organellar volumes and surface areas and describe the mitochondrial subcompartment and its parameterization. Morphological models are detailed in Online Resource 2, and all model parameters are listed in Online Resource 3.

1.1 Model variables

The rates of change of extracellular ($[Ca^{2+}]_e$) and intra-ER ($[Ca^{2+}]_{ER}$) Ca^{2+} concentrations are:

$$\frac{d[\mathrm{Ca}^{2+}]_e}{dt} = -\frac{A}{VF} J_{\mathrm{NCX}}, \tag{S1}$$

$$\frac{d[\mathrm{Ca}^{2+}]_{\mathrm{ER}}}{dt} = \frac{A\sqrt{r_{\mathrm{ER}}}}{VFr_{\mathrm{ER}}}(-J_{\mathrm{IP_3R}} + J_{\mathrm{SERCA}} - J_{\mathrm{CaERL}}), \tag{S2}$$

where A is the lateral surface area of the compartment, V the volume, F the Faraday constant, and r_{ER} the ER-to-cytosol volume ratio (Eq. 5). J_{NCX} is the NCX current density (Eq. S17), $J_{\mathrm{IP_3R}}$ the Ca^{2+} current density through IP_3 receptor channels (Eq. S12), J_{SERCA} the SERCA current density (Eq. S14), and J_{CaERL} the ER leak current density (Eq. S13).

The intracellular IP_3 concentration evolves as:

$$\frac{d[\mathrm{IP_3}]}{dt} = S_{\mathrm{PLC}\beta_{\mathrm{Glu}}} + S_{\mathrm{PLC}\beta_{\mathrm{DA}}} + S_{\mathrm{PLC}\delta} - D_{\mathrm{IP_3-3K}} - D_{\mathrm{IP-5P}}, \tag{S3}$$

where $S_{\mathrm{PLC}\beta_{\mathrm{Glu}}}$ (Eq. S20) and $S_{\mathrm{PLC}\beta_{\mathrm{DA}}}$ (Eq. S21) represent IP_3 production via PLCβ activation by mGluR and by D_1/α_1 receptors, respectively. $S_{\mathrm{PLC}\delta}$ is IP_3 synthesis by PLCδ (Eq. S22). $D_{\mathrm{IP_3-3K}}$ (Eq. S23) and $D_{\mathrm{IP-5P}}$ (Eq. S24) denote, respectively, Ca^{2+}-dependent degradation by IP_3-3K and Ca^{2+}-independent degradation by IP-5P.

The fraction of open IP_3 receptors h is governed by:

$$\frac{dh}{dt} = a_2 \left(d_2 \frac{[\mathrm{IP_3}] + d_1}{[\mathrm{IP_3}] + d_3}(1-h) - [\mathrm{Ca^{2+}}]_i h \right), \tag{S4}$$

with $a_2 = 0.2$ µM^{-1}s^{-1} (rate constant for Ca^{2+} inhibition of IP_3R), $d_2 = 1.049$ µM (IP_3 Ca^{2+} dissociation constant), and $d_1 = 0.13$ µM, $d_3 = 0.9434$ µM (IP_3 dissociation constants).

The dynamics of intracellular and extracellular Na^+ and K^+ are:

$$\frac{d[\mathrm{Na^+}]_i}{dt} = \frac{A}{VF}(3J_{\mathrm{GluT}} - 3J_{\mathrm{NKA}} - 3J_{\mathrm{NCX}} - J_{\mathrm{NaL}}) \tag{S5}$$

$$\frac{d[\mathrm{Na^+}]_e}{dt} = -\frac{A}{VF}(-3J_{\mathrm{GluT}} + 3J_{\mathrm{NKA}} + 3J_{\mathrm{NCX}} + J_{\mathrm{NaL}}), \tag{S6}$$

$$\frac{d[\mathrm{K^+}]_i}{dt} = \frac{A}{VF}(-J_{\mathrm{GluT}} + 2J_{\mathrm{NKA}} - J_{\mathrm{KL}}) \tag{S7}$$

$$\frac{d[\mathrm{K^+}]_e}{dt} = \frac{A}{VF}(J_{\mathrm{GluT}} - 2J_{\mathrm{NKA}} + J_{\mathrm{KL}}), \tag{S8}$$

where J_{GluT} is the glutamate transporter current density (Eq. S15), J_{NKA} the Na^+/K^+-ATPase pump current density (Eq. S16), and J_{NaL}, J_{KL} the Na^+ and K^+ leak current densities (Eq. S18, Eq. S19). Integers denote the effective charge exchanged per transport cycle.

The membrane potential v is modeled as:

$$\begin{aligned}\frac{dv}{dt} = -\frac{1}{C_m}(&-2J_{\mathrm{IP_3R}} + 2J_{\mathrm{SERCA}} - 2J_{\mathrm{MCU}} + 2J_{\mathrm{mPTP}} - 2J_{\mathrm{CaERL}} + \\ &J_{\mathrm{NCX}} - 2J_{\mathrm{GluT}} + J_{\mathrm{NKA}} + J_{\mathrm{NaL}} + J_{\mathrm{KL}}),\end{aligned} \tag{S9}$$

where $C_m = 0.01$ F/m^2 is the astrocyte membrane capacitance. Integers represent effective charge.

Extracellular glutamate ([Glu]) and dopamine ([DA]) concentrations are:

$$\frac{d[\mathrm{Glu}]}{dt} = -G_{\mathrm{Glu}}[\mathrm{Glu}] + \rho_{\mathrm{Glu}}\delta(t - t_{\mathrm{spike}}), \tag{S10}$$

$$\frac{d[\mathrm{DA}]}{dt} = -G_{\mathrm{DA}}[\mathrm{DA}] + \rho_{\mathrm{DA}}\delta(t - t_{\mathrm{spike}}), \tag{S11}$$

where t_{spike} denotes glutamate or dopamine release events. $G_{\mathrm{Glu}} = 100$ s^{-1} and $G_{\mathrm{DA}} = 4.201$ s^{-1} are the glutamate and dopamine decay rates, respectively, and $\rho_{\mathrm{Glu}} = 0.5$ µM and $\rho_{\mathrm{DA}} = 3$ µM are the glutamate and dopamine increments per release, respectively. Both were modeled as Poisson processes with constant frequencies ν_g (glutamate) and ν_d (dopamine) during each simulation trial.

1.2 Current densities

The current densities J_{NCX}, J_{NKA}, J_{GluT}, $J_{\mathrm{IP_3R}}$, J_{SERCA}, J_{CaERL}, J_{NaL}, J_{KL}, J_{mPTP}, and J_{MCU} are expressed in pA/µm^2 so that they can be combined consistently in the equation governing the compartment potential v (Eq. S9). For mechanisms initially formulated as concentration rates (in µM/s), multiplication by FV/A converts the rate to current density; conversely, multiplication by A/FV converts a current density back to a concentration rate when it enters a concentration-balance equation. In the following equations, we adopt the Hill function notation

$$\mathcal{H}_n(x, k) \coloneqq x^n/(x^n + k^n),$$

in which n is the Hill coefficient and k represents the half-saturation constant.

The Ca^{2+} current density through IP_3R channels, the ER leak current density, and the SERCA pump current density are:

$$J_{\mathrm{IP_3R}} = \frac{FV}{A} r_C \left(\mathcal{H}_1\left([\mathrm{IP_3}], d_1\right)\right)^3 \left(\mathcal{H}_1\left([\mathrm{Ca^{2+}}]_i, d_5\right)\right)^3 h^3([\mathrm{Ca^{2+}}]_{\mathrm{ER}} - [\mathrm{Ca^{2+}}]_i), \tag{S12}$$

$$J_{\mathrm{CaERL}} = \frac{FV}{A} r_L([\mathrm{Ca^{2+}}]_{\mathrm{ER}} - [\mathrm{Ca^{2+}}]_i), \tag{S13}$$

$$J_{\mathrm{SERCA}} = \frac{FV}{A} v_{\mathrm{ER}} \mathcal{H}_2\left([\mathrm{Ca^{2+}}]_i, K_{\mathrm{ER}}\right), \tag{S14}$$

where $r_C = 6$ s^{-1} is the maximum Ca^{2+} release rate, $d_1 = 0.13$ µM and $d_5 = 0.08234$ µM are, respectively, the IP_3 and Ca^{2+} dissociation constants, and h represents the proportion of open IP_3R channels (Eq. S4). $r_L = 0.11$ s^{-1} is the Ca^{2+} leakage rate, $v_{ER} = 11.93$ µM/s is the maximum SERCA uptake rate, and $K_{ER} = 0.1$ µM is the SERCA Ca^{2+} affinity.

The GluT current density is:

$$J_{\mathrm{GluT}} = J_{\mathrm{GluTmax}} \mathcal{H}_1\left([\mathrm{K^+}]_i, K_{\mathrm{GluTmK}}\right) \mathcal{H}_3\left([\mathrm{Na^+}]_i, K_{\mathrm{GluTmN}}\right) \mathcal{H}_1\left([\mathrm{Glu}], K_{\mathrm{GluTmg}}\right), \tag{S15}$$

where $J_{\mathrm{GluTmax}} = 0.68$ pA/µm^2 is the maximum transport rate, and $K_{\mathrm{GluTmK}} = 5{,}000$ µM, $K_{\mathrm{GluTmN}} = 15{,}000$ µM and $K_{\mathrm{GluTmg}} = 34$ µM are the half-saturation constants of K^+, Na^+ and glutamate.

The current density through the Na^+/K^+-ATPase (NKA) pump is:

$$J_{\mathrm{NKA}} = J_{\mathrm{NKAmax}} \mathcal{H}_{1.5}\left([\mathrm{Na^+}]_i, K_{\mathrm{NKAmN}}\right) \mathcal{H}_1\left([\mathrm{K^+}]_e, K_{\mathrm{NKAmK}}\right), \tag{S16}$$

where $J_{\mathrm{NKAmax}} = 1.52$ pA/µm^2 is the pump rate, and $K_{\mathrm{NKAmN}} = 10{,}000$ µM and $K_{\mathrm{NKAmK}} = 1{,}500$ µM are the Na^+ and K^+ half-saturation constants, respectively.

The current density through the Na^+/Ca^{2+}-exchanger (NCX) is:

$$J_{\mathrm{NCX}} = J_{\mathrm{NCXmax}} \mathcal{H}_3\left([\mathrm{Na^+}]_e, K_{\mathrm{NCXmN}}\right) \mathcal{H}_1\left([\mathrm{Ca^{2+}}]_e, K_{\mathrm{NCXmC}}\right) \cdot \frac{\frac{[\mathrm{Na^+}]_i^3}{[\mathrm{Na^+}]_e^3} \exp\left(\eta \frac{vF}{RT}\right) - \frac{[\mathrm{Ca^{2+}}]_i}{[\mathrm{Ca^{2+}}]_e} \exp\left((\eta - 1)\frac{vF}{RT}\right)}{1 + k_{\mathrm{sat}} \exp\left((\eta-1)\frac{vF}{RT}\right)}, \tag{S17}$$

where $J_{\mathrm{NCXmax}} = 0.0001$ pA/µm^2 is the exchange rate, $K_{\mathrm{NCXmN}} = 87{,}500$ µM and $K_{\mathrm{NCXmC}} = 1{,}380$ µM are, respectively, the half-saturation constants of Na^+ and Ca^{2+}, $\eta = 0.35$ is the energy barrier of the NCX voltage dependence, $R = 8.314$ J/molK is the gas constant, $T = 303.16$ K is the temperature and $k_{\mathrm{sat}} = 0.1$ is the saturation constant.

The Na^+ and K^+ leak current densities through the astrocyte membrane are:

$$J_{\mathrm{NaL}} = g_{\mathrm{NaL}}(v - E_{\mathrm{Na}}) \tag{S18}$$

$$J_{\mathrm{KL}} = g_{\mathrm{KL}}(v - E_{\mathrm{K}}), \tag{S19}$$

where $g_{\mathrm{NaL}} = 13.483$ S/m^2 and $g_{\mathrm{KL}} = 145.814$ S/m^2 are the Na^+ and K^+ conductances, respectively, and $E_{\mathrm{Na}} = 61$ mV and $E_{\mathrm{K}} = -94$ mV are their corresponding reversal potentials.

1.3 IP_3 synthesis and degradation

The activation of mGluR by glutamate triggers IP_3 synthesis, which activates the PLCβ pathway. This enzyme cleaves phosphatidylinositol 4,5-bisphosphate (PIP_2) into diacyl glycerol (DAG) and inositol 1,4,5-trisphosphate (IP_3) from the astrocyte membrane, a process that is inhibited by intracellular Ca^{2+} [1, 2]. This mechanism is synthesized by the following equation:

$$S_{\mathrm{PLC}\beta_{\mathrm{Glu}}} = v_\beta \mathcal{H}_\alpha \left([\mathrm{Glu}], K_R + K_p \frac{[\mathrm{Ca}^{2+}]_i}{[\mathrm{Ca}^{2+}]_i + K_\pi} \right), \tag{S20}$$

where $v_\beta = 0.674$ µM/s is the rate of IP_3 synthesis by the PLCβ, $\alpha = 0.7$ is the Hill coefficient for this process, $K_R = 1.3$ µM is the mGluR glutamate affinity, $K_p = 10$ µM is the Ca^{2+}-dependent inhibition of PLCβ and $K_\pi = 0.6$ µM is the PLCβ Ca^{2+} affinity constant.

Similar to glutamate, dopamine also triggers Ca^{2+} release from ER through the PLCβ pathway by activating D_1/α_1 receptors [3, 4]. This process is modeled as:

$$S_{\mathrm{PLC}\beta_{\mathrm{DA}}} = v_{\mathrm{DA}} \mathcal{H}_\beta \left([\mathrm{DA}], K_{\mathrm{DA}} + K_p \frac{[\mathrm{Ca}^{2+}]_i}{[\mathrm{Ca}^{2+}]_i + K_\pi} \right), \tag{S21}$$

where $v_{\mathrm{DA}} = 0.025$ µM/s is the IP_3 synthesis rate by PLCβ, $\beta = 0.5$ is the Hill coefficient, and $K_{\mathrm{DA}} = 5$ µM is the dopaminergic receptors dopamine affinity. More details on the parameter fitting are given in [5].

In addition to the PLCβ pathway, which depends on activation of G-protein coupled receptors, IP_3 is also synthesized by endogenous PLCδ, which is Ca^{2+}-dependent and inhibited by intracellular IP_3. This process is given by [1, 2]:

$$S_{\mathrm{PLC}\delta} = \frac{v_\delta}{1 + \frac{[\mathrm{IP}_3]}{\kappa_\delta}} \mathcal{H}_2 \left([\mathrm{Ca}^{2+}]_i, K_{\mathrm{PLC}\delta} \right), \tag{S22}$$

where $v_\delta = 0.025$ µM/s is the maximum rate of IP_3 production by PLCδ, $\kappa_\delta = 1.5$ µM is the PLCδ inhibition constant and $K_{\mathrm{PLC}\delta} = 0.1$ µM is the Ca^{2+}-dependent inhibition of PLCδ.

IP_3 can be degraded by two enzymes, the Ca^{2+}-dependent IP_3-3K and the Ca^{2+}-independent IP-5P, These two processes are modeled as [1, 2]:

$$D_{\mathrm{IP}_3-3\mathrm{K}} = v_{3K} \mathcal{H}_4 \left([\mathrm{Ca}^{2+}]_i, K_D \right) \mathcal{H}_1 \left([\mathrm{IP}_3], K_3 \right) \tag{S23}$$

$$D_{\mathrm{IP}-5\mathrm{P}} = r_{5P} [\mathrm{IP}_3], \tag{S24}$$

where $v_{3K} = 2$ µM/s is the IP_3-3K degradation rate, $K_D = 0.7$ µM and $K_3 = 1$ µM are the IP_3-3K Ca^{2+} and IP_3 affinities, respectively, and $r_{5P} = 0.04$ s^{-1} is the IP-5P degradation rate.

1.4 Compartmental coupling

The effect of the diffusive flux of ion/molecule X from compartment k to compartment j on the concentration of X in compartment j is given by [5, 6]:

$$\left(\frac{d[\mathrm{X}]_j}{dt} \right)_{\mathrm{diff}} = \frac{D_{\mathrm{X}} A_j}{V_j d_{jk}} \left([\mathrm{X}]_k - [\mathrm{X}]_j \right) = F_{\mathrm{X},k\to j} \left([\mathrm{X}]_k - [\mathrm{X}]_j \right), \tag{S25}$$

where D_{X} is the diffusion coefficient of the ion/molecule X, A_j is the area of the cross-section of compartment j, V_j is the volume of compartment j, and d_{jk} is the distance between the centers of

compartments j and k. $F_{\mathrm{X},k\to j}$ is the coupling strength for X from compartment k to compartment j with dimension of s^{-1}. Considering the geometry of the compartments in our model, the coupling strength can take one of the following expressions:

a) When k is the soma and j is a cylindrical compartment,

$$F_{\mathrm{X},k\to j} \equiv F_{\mathrm{X,s\to c}} = \frac{D_{\mathrm{X}}}{\ell\,(r_s + \ell/2)}, \tag{S26}$$

where r_s is the soma radius. Since $r_s = 20$ µm in our model,

$$F_{\mathrm{X},s\to c} = 0.049 \cdot n\,(D_{\mathrm{X}})\ \mathrm{s}^{-1},$$

where $n(D_X)$ is the numerical value of the diffusion coefficient of ion/molecule X.

b) When compartment k is a cylindrical compartment and j is the soma,

$$F_{\mathrm{X},k\to j} \equiv F_{\mathrm{X,c\to s}} = \frac{D_{\mathrm{X}} r_c^2}{\frac{4}{3} r_s^3 \left(r_s + \frac{\ell}{2}\right)}, \tag{S27}$$

where r_c is the radius of the cylindrical compartment. Since in our model the cylindrical compartments connected to soma have all the same radius $r_c = 2$ µm,

$$F_{\mathrm{X},c\to s} = 0.183 \times 10^{-4} \cdot n\,(D_{\mathrm{X}})\ \mathrm{s}^{-1}.$$

c) When both compartments j and k are cylindrical,

$$F_{\mathrm{X},k\to j} \equiv F_{\mathrm{X,cyl}} = \frac{D_{\mathrm{X}}}{\ell^2}. \tag{S28}$$

Since $\ell = 1$ µm in our model,

$$F_{\mathrm{X,cyl}} = n\,(D_{\mathrm{X}})\ \mathrm{s}^{-1}.$$

1.5 Volume ratio

Both the cytosolic compartment and its organellar subcompartments (ER and mitochondria) are represented as cylinders of the same length l. Let ϕ and ϕ_{sub} denote the radii of the cytosolic compartment and an organellar subcompartment, respectively. Defining the subcompartment-to-cytosol volume ratio as

$$r_{\mathrm{sub}} \equiv \frac{V_{\mathrm{sub}}}{V},$$

and using $V = \pi\phi^2 l$ and $V_{\mathrm{sub}} = \pi\phi_{\mathrm{sub}}^2 i$, we obtain

$$r_{\mathrm{sub}} = \frac{\phi_{\mathrm{sub}}^2}{\phi^2},$$

and therefore

$$\phi_{\mathrm{sub}} = \sqrt{r_{\mathrm{sub}}}\,\phi. \tag{S29}$$

Because A and A_{sub} denote the lateral surface areas of the corresponding cylinders,

$$A = 2\pi\phi l, \quad \text{and} \quad A_{\mathrm{sub}} = 2\pi\phi_{\mathrm{sub}} l,$$

which gives

$$A_{\mathrm{sub}} = \sqrt{r_{\mathrm{sub}}}A. \tag{S30}$$

Thus, for the ER and mitochondrial subcompartments, the lateral surface areas are $A\sqrt{r_{\mathrm{ER}}}$ and $A\sqrt{r_m}$, respectively. The value of r_m was determined by the compartment geometry and the fitted mitochondrial-radius relationship shown in Fig. S1.

1.6 Mitochondria model

We represented the total mitochondrial volume in each compartment as a single cylindrical mitochondrial subcompartment with fixed length $l = 1$ µm. Thicker mitochondria localize to endfeet of astrocytic processes, whereas thinner mitochondria occur in non-endfoot regions [7].

To relate the mitochondrial radius ϕ_m to the compartment radius Φ, we first fitted a linear relation

$$\phi_m(\Phi) = a\Phi + b,$$

for $\Phi \in$ [0.25 µm, 2 µm] with ϕ_m(0.25 µm) = 0.19 µm and ϕ_m(2 µm) = 0.149 µm (Fig. **S1a**) [7]. Within this interval we assumed that mitochondrial radius scales linearly with compartment radius.

To extend ϕ_m to arbitrary Φ, we used the linear fit above and imposed ϕ_m(20 µm) = 0.149 µm. With these constraints, we fitted the sigmoidal function

$$\phi_m(\Phi) = \frac{a}{1 + \exp(b\Phi - c)} + d,$$

with $a = 0.024$ µm, $b = 2.50$ µm^{-1}, $c = 2.58$, and $d = 0.073$ µm (Fig. **S1A**). Finally, to scale fluxes between the mitochondrial and cytosolic subspaces, we used the ratio $r_m = V_m/V_i$, where V_m is the mitochondrial volume and V_i the compartment volume (Fig. **S1b**).

The mitochondrial model was developed from prior formulations of Ca^{2+} uptake via MCU and release via mPTP [8, 9]. The kinetic parameters V_{MCUmax}, K_{MCU}, V_{mPTPmax} and K_{mPTP} were adopted from the mitochondrial Ca^{2+} formulations of Friel and Tsien [8] and Pivovarova et al. [9] and were not fitted to the present simulations. The parameter l_{mPTP} was adjusted so that the unstimulated model exhibits a stable equilibrium with resting intramitochondrial Ca^{2+} concentration $[Ca^{2+}]_{\mathrm{m}} = 0.3$ µM. Confidence intervals applicable to these kinetic parameters were not reported in the source models; therefore, no uncertainty intervals were assigned here.

2 Stimulation Protocols and Model Outputs

At the beginning of each simulation, all variables in each compartment were set to their respective resting states, ensuring equilibrium in the absence of inputs. Each trial lasted 100 s. Glutamatergic and dopaminergic inputs were modeled as Poisson processes with constant frequencies ν_g and ν_d, respectively. Stimulated compartments are denoted as CX–Y, where X and Y indicate the first and last stimulated compartments. Unless otherwise specified, glutamate stimulated the distal

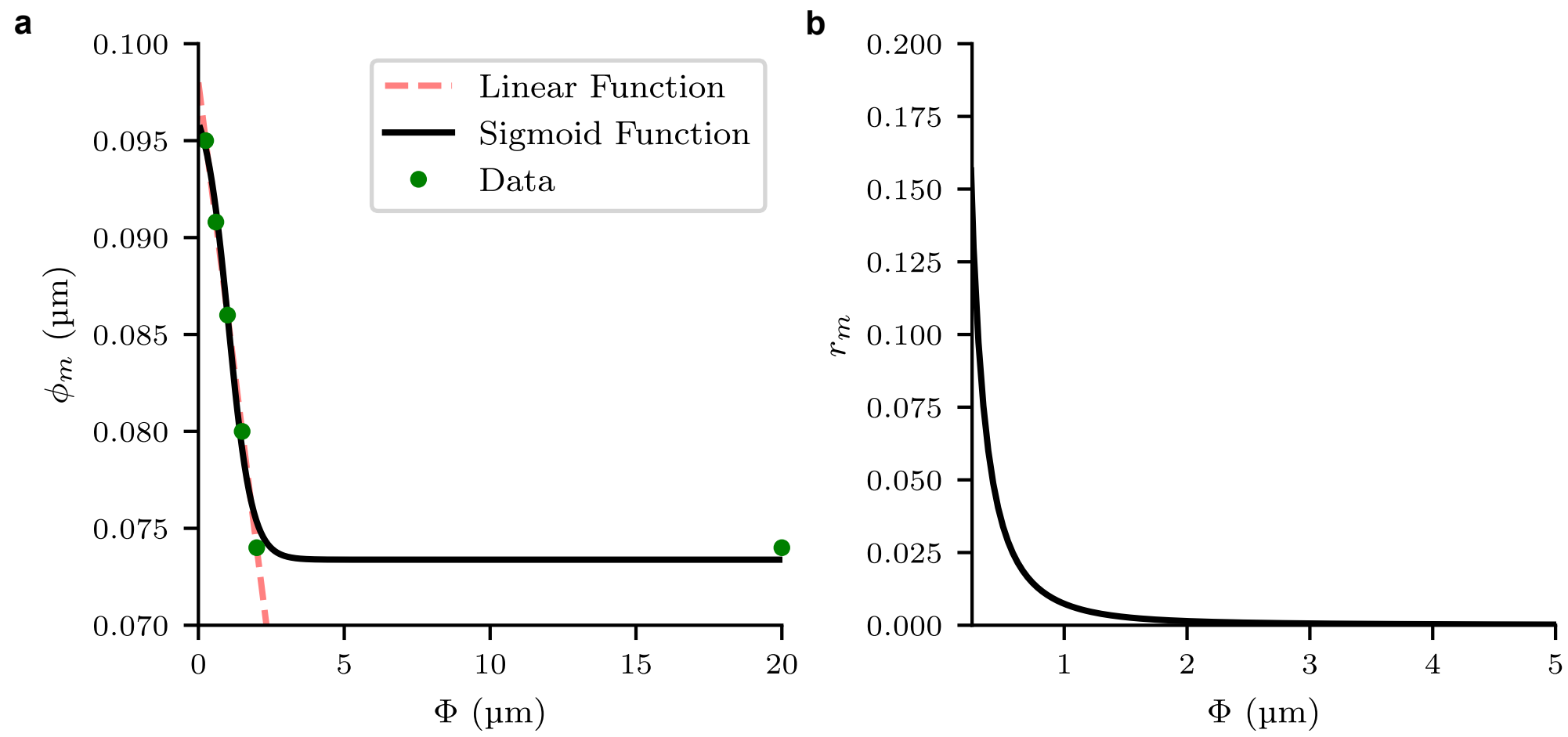

Fig S1: **Mitochondrial radius ϕ_m (a) and mitochondrial-to-compartment volume ratio r_m (b) as a function of the compartment radius Φ.**

compartments (C18–20), whereas dopamine stimulated all compartments (C1–20). Exceptions were made in tests in which the positions of mitochondria and glutamatergic input were varied.

Ca^{2+} signals were counted whenever the intracellular Ca^{2+} concentration crossed the threshold $[Ca^{2+}]_{th} = 0.15$ µM [5, 10]. The oscillation frequency in each compartment, f_{Ca2+}, was calculated as the number of threshold crossings per trial, normalized by the trial duration (100 s). Propagation extent was defined as the number of consecutively activated compartments, counted from the terminal end. An individual threshold-crossing transient is referred to as a Ca^{2+} signal or event, whereas repeated signals during a stimulation period are referred to as Ca^{2+} oscillations. The broader term Ca^{2+} response refers to the overall activity elicited by a stimulation condition, including signal frequency, amplitude, and propagation extent.

2.1 Protocols and outputs for the unipolar morphology

For the unipolar input-interaction analysis, distal compartments C18–20 received glutamatergic input at ν_g = 0-5 Hz, while dopamine was applied globally at ν_d = 0, 0.005, 0.05, or 0.5 Hz. Propagation extent and $f_{Ca^{2+}}$ were quantified as described above (see Figs. 7–8 in the main text).

2.2 Protocols and outputs for the bifurcated-terminal morphology

For the bifurcated-terminal morphology, dopamine was applied to the entire astrocyte (ν_d = 0, 0.005, 0.05, or 0.5 Hz), one branchlet (C18–20) received glutamate at varying frequency (ν_g = 0-10 Hz), and the second branchlet (C22–24) received a fixed weak glutamatergic input (ν_g = 1 Hz). The corresponding propagation and frequency analyses are presented in Figs. 9-10 of the main text.

3 Model Response to Glutamate and Dopamine

To examine the effect of incorporating mitochondrial mechanisms into astrocytic Ca^{2+} regulation, we first analyzed the unipolar morphology under simultaneous glutamatergic stimulation ($\nu_g = 5$ Hz, C18-20) and dopaminergic stimulation ($\nu_d = 1$ Hz, C1-20). We compared $f_{Ca^{2+}}$, Ca^{2+} signal amplitude, intramitochondrial Ca^{2+}, and intracellular IP_3 concentration (see Fig. 1 of the main text). The main text shows the responses of compartments C13–16; here, we report responses from the remaining compartments, model trajectories in the Ca^{2+}–IP_3 space (Fig. **S2**), and responses to glutamatergic or dopaminergic input alone (Fig. **S3**).

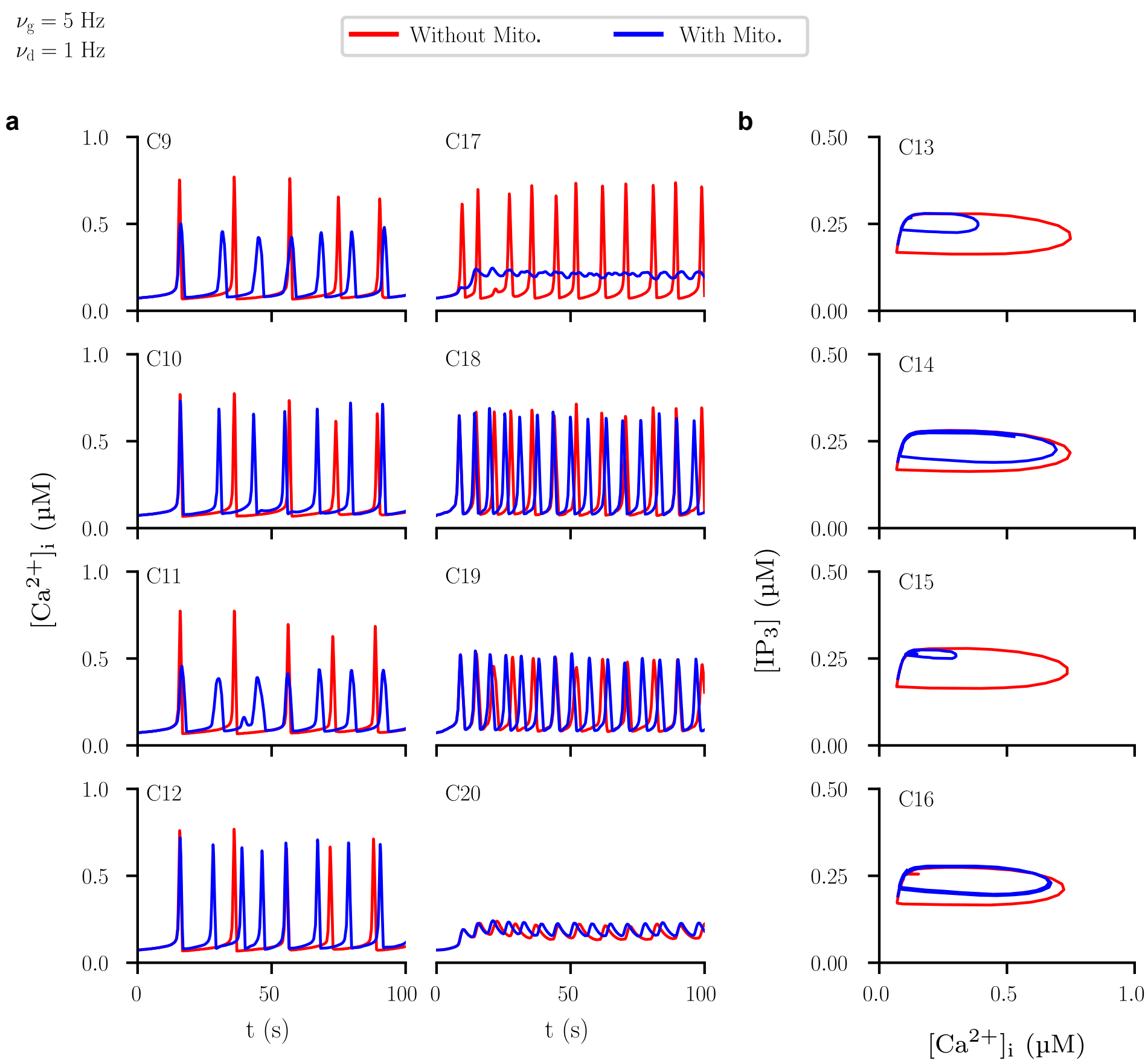

Fig S2: **Model response under combined glutamatergic and dopaminergic stimulation ($\nu_g = 5$ Hz, C18-20; $\nu_d = 1$ Hz, C1-20).** (**a**) Intracellular Ca^{2+} concentration traces across compartments. In mitochondria-containing compartments, Ca^{2+} oscillations have smaller amplitude, yet mitochondria increase $f_{Ca^{2+}}$ globally. (**b**) Trajectories in the Ca^{2+}-IP_3 phase space (C13–C16). Compartments with mitochondria exhibit smaller Ca^{2+} amplitudes but higher overall IP_3 levels.

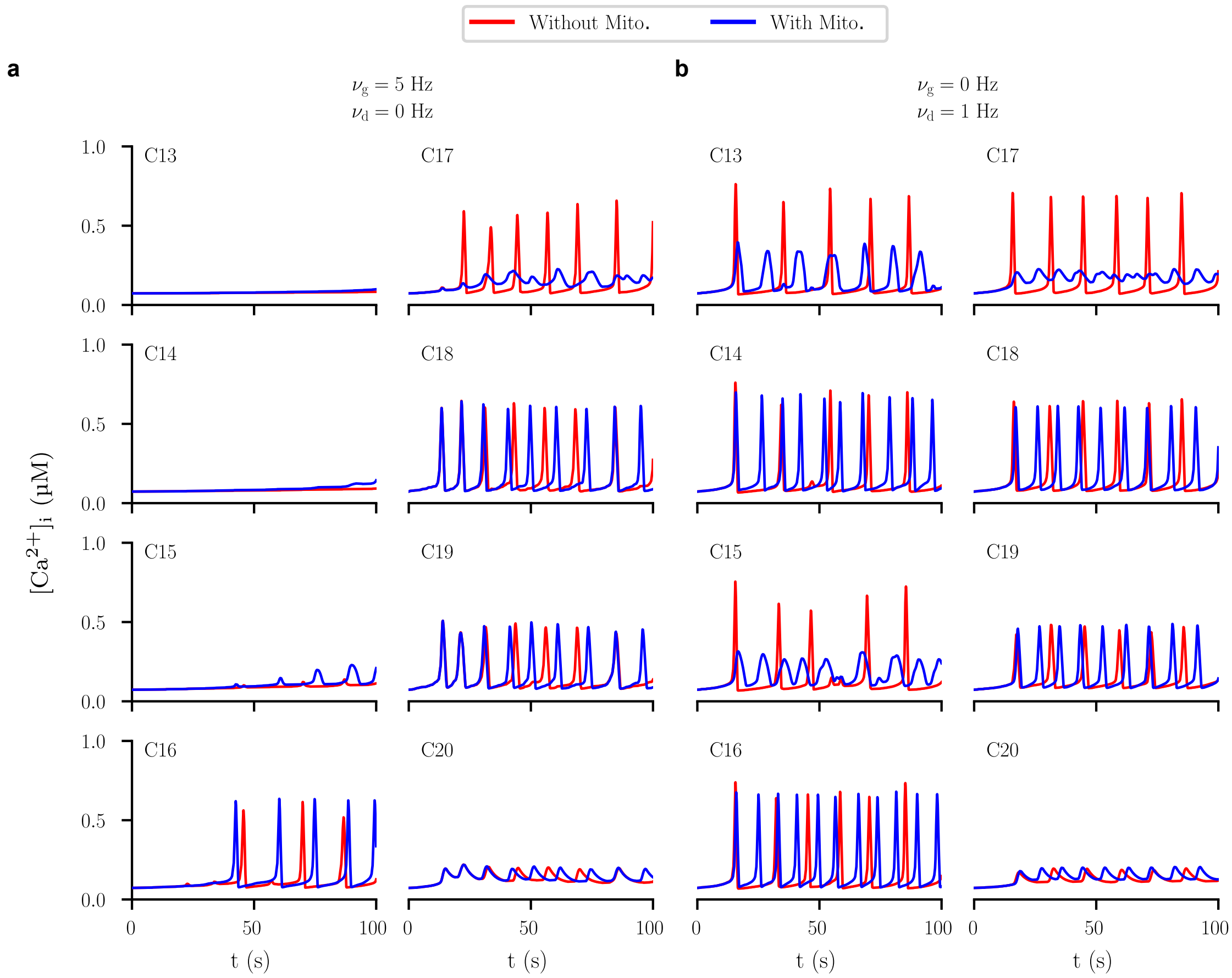

Fig S3: **Model response to single-transmitter stimulation.** (**a**) Glutamate only ($\nu_g = 5$ Hz, C18-20). (**b**) Dopamine only ($\nu_d = 1$ Hz, C1-20). In both cases, mitochondria reduce Ca^{2+} amplitude in compartments that contain them but increase $f_{Ca^{2+}}$ across the cell. With glutamate alone, responses reach up to C15 with mitochondria; dopamine alone activates all compartments.

4 Parameter Sensitivity and Robustness Analyses

To evaluate the robustness of the mitochondrial effects, identify mechanisms underlying them, and determine whether changes in selected parameters could restore model outputs toward the reference response, we systematically varied parameters controlling astrocytic Ca^{2+} signaling, mitochondrial Ca^{2+} handling, IP_3 turnover, and SERCA-mediated Ca^{2+} uptake. Unless otherwise specified, all parameters were maintained at their reference values listed in Online Resource 3. The analyses below complement the parameter variations presented in the main text.

For parameter-sensitivity analyses designed to test whether changing a model parameter can counteract the effect introduced by mitochondrial Ca^{2+} handling and restore the response toward the reference condition, we used the model without mitochondria at the default parameter value

as a fixed reference. For an output variable X and a varied parameter p, we therefore define the deviation from the reference response as

$$\Delta_{\text{ref}} X(p) = X_{\text{mito}}(p) - X_{\text{no mito}}(p_{\text{ref}}), \tag{S31}$$

where p_{ref} is the default value of the parameter, indicated by a vertical yellow dashed line in the appropriate graphs. Thus, $\Delta_{\text{ref}} X(p) = 0$ indicates that the response of the model with mitochondria at parameter value p matches the reference response of the model without mitochondria at p_{ref}. Positive and negative values indicate responses above and below this reference, respectively. The corresponding curves obtained by varying p in the model without mitochondria are also shown to illustrate the direct effect of the parameter on the reference model, but these values are not used point-by-point in the calculation of Δ_{ref}.

For analyses intended instead to compare the mitochondrial and non-mitochondrial models under the same stimulation or parameter condition, differences are calculated pointwise as $X_{\text{mito}} - X_{\text{no mito}}$, as specified in the corresponding figure captions.

4.1 General sensitivity of Ca^{2+} signaling parameters

We first examined the sensitivity of $f_{\text{Ca2+}}$ to parameters controlling IP_3R inhibition, IP_3 degradation, and PLCβ regulation. We varied a_2, r_{5P}, K_p, and K_π around their reference values while comparing models with and without mitochondria (Fig. **S4**). These analyses provide a general assessment of whether the mitochondrial effect depends strongly on parameters inherited from the underlying astrocytic Ca^{2+} model.

4.2 Mitochondrial Ca^{2+} uptake and release

To assess the sensitivity of astrocytic Ca^{2+} responses to mitochondrial Ca^{2+} handling, we systematically varied the maximum MCU-mediated Ca^{2+} uptake rate (V_{MCUmax}) and mPTP-mediated release parameter (l_{mPTP}). The unipolar morphology was stimulated with glutamate (C18-20, ν_g = 5 Hz) and dopamine (C1-20, $\nu_d = 1$ Hz). Results for C15 (with mitochondria) and C16 (without mitochondria) are shown in Fig. 3 of the main text; Fig. **S5** extends this analysis to compartments C13–16 across three values of l_{mPTP}.

4.3 IP_3-Ca^{2+} Interaction

To assess how mitochondria enhance astrocytic responses, we varied parameters controlling the Ca^{2+}–IP_3 interaction:

- K_D (Eq. S23): Ca^{2+} affinity for IP_3-3K (Ca^{2+}-dependent IP_3 degradation). Lower K_D increases Ca^{2+}-dependent degradation.
- K_p: Ca^{2+}-dependent inhibition of PLCβ. Higher values strengthen inhibition;
- K_π: Ca^{2+} affinity for PLCβ. Lower values increase the Ca^{2+} inhibition of PLCβ;
- r_{5P}: Rate of Ca^{2+}-independent IP_3 degradation by IP-5P. Higher values accelerate degradation. This is a control case for IP_3 degradation.

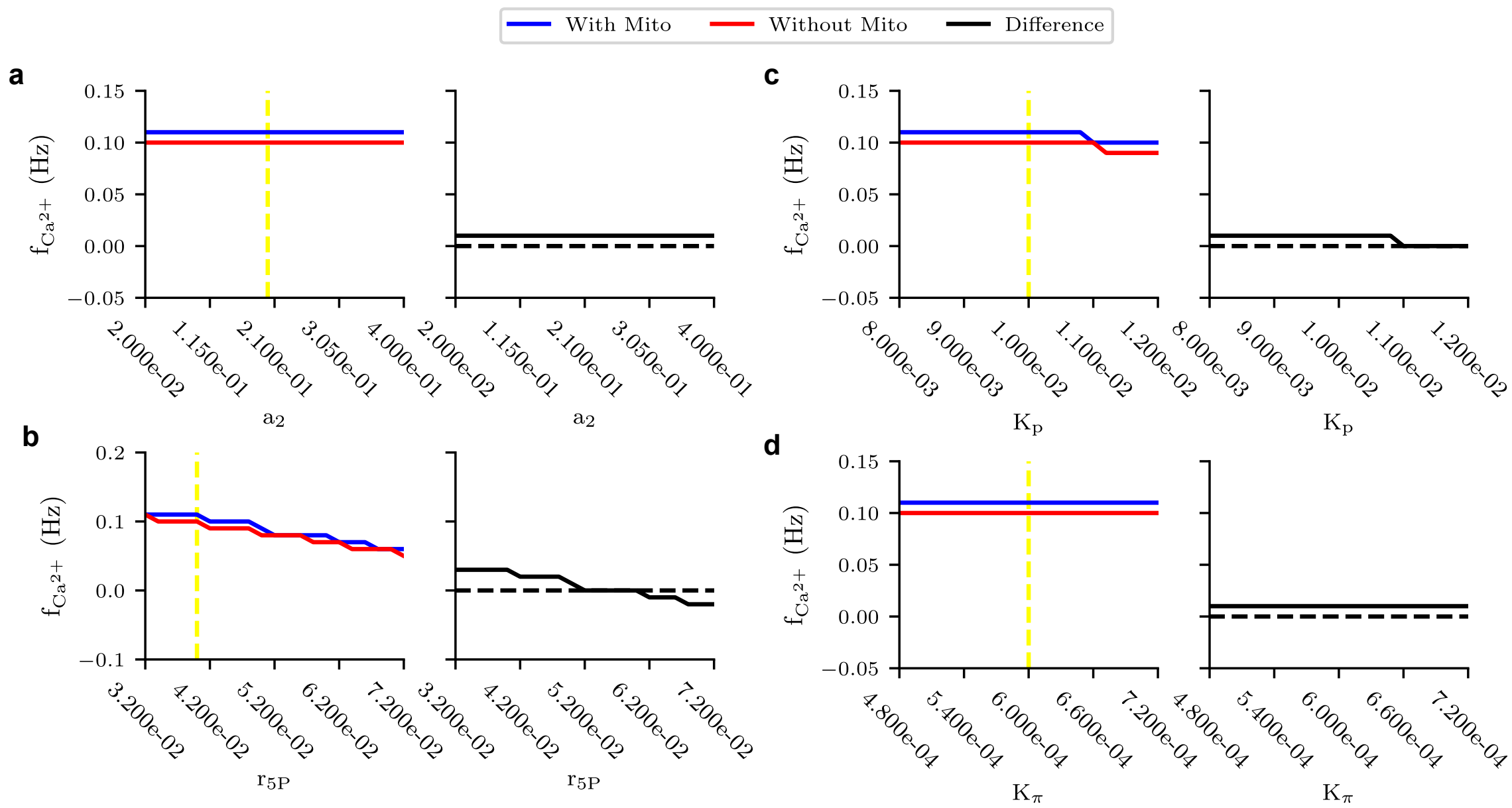

Fig S4: **Sensitivity of Ca^{2+} oscillation frequency to parameters of the astrocytic Ca^{2+} model.** $f_{Ca^{2+}}$ in models with and without mitochondria and their difference from reference as a function of (**a**) a_2, the rate constant for Ca^{2+} inhibition of IP_3Rs; (**b**) r_{5P}, the Ca^{2+}-independent IP_3 degradation rate; (**c**) K_p, the Ca^{2+}-dependent inhibition parameter of PLCβ; and (**d**) K_π, the PLCβ Ca^{2+}affinity constant. For each parameter, the vertical yellow dashed line indicates its default value p_{ref}. The difference-from-reference curve is calculated as $\Delta_{\mathrm{ref}} f_{Ca^{2+}}(p) = f^{\mathrm{mito}}_{Ca^{2+}}(p) - f^{\mathrm{no\ mito}}_{Ca^{2+}}(p_{\mathrm{ref}})$, as defined in (S31). The horizontal dashed line indicates $\Delta_{\mathrm{ref}} f_{Ca^{2+}} = 0$, i.e. recovery of the reference response.

The unipolar model was stimulated with glutamate ($\nu_g = 5$ Hz, C18-20) and dopamine ($\nu_d = 5$ Hz, C1-20). We compared models with and without mitochondria using three metrics:

- $f_{Ca^{2+}}$;
- Time-averaged Ca^{2+} current density through IP_3R (J_{IP_3R}, Eq. S12);
- Maximum Ca^{2+}-dependent IP_3-3K degradation (D_{IP_3-3K}, Eq. S23);

In the main text, we report only the effect of varying K_D (Fig. 5). Here, we present additional results for the other parameters (Fig. **S6**) and complementary metrics (Figs. **S7**–**S8**).

Finally, we tested reduced $V_{\mathrm{MCUmax}} = 41.25 \times 10^3\ \mathrm{s}^{-1}$ combined with variation of K_D. The main text shows the time series for $K_D = 0.84$ µM. Here, we provide the full range of results for $f_{Ca^{2+}}$, J_{IP_3R}, and D_{IP_3-3K} (Fig. **S9**).

4.4 SERCA sensitivity

To determine whether the mitochondrial effect was robust to changes in an ATP-dependent Ca^{2+}-handling mechanism, we varied the maximum SERCA uptake rate v_{ER}. Increasing SERCA-

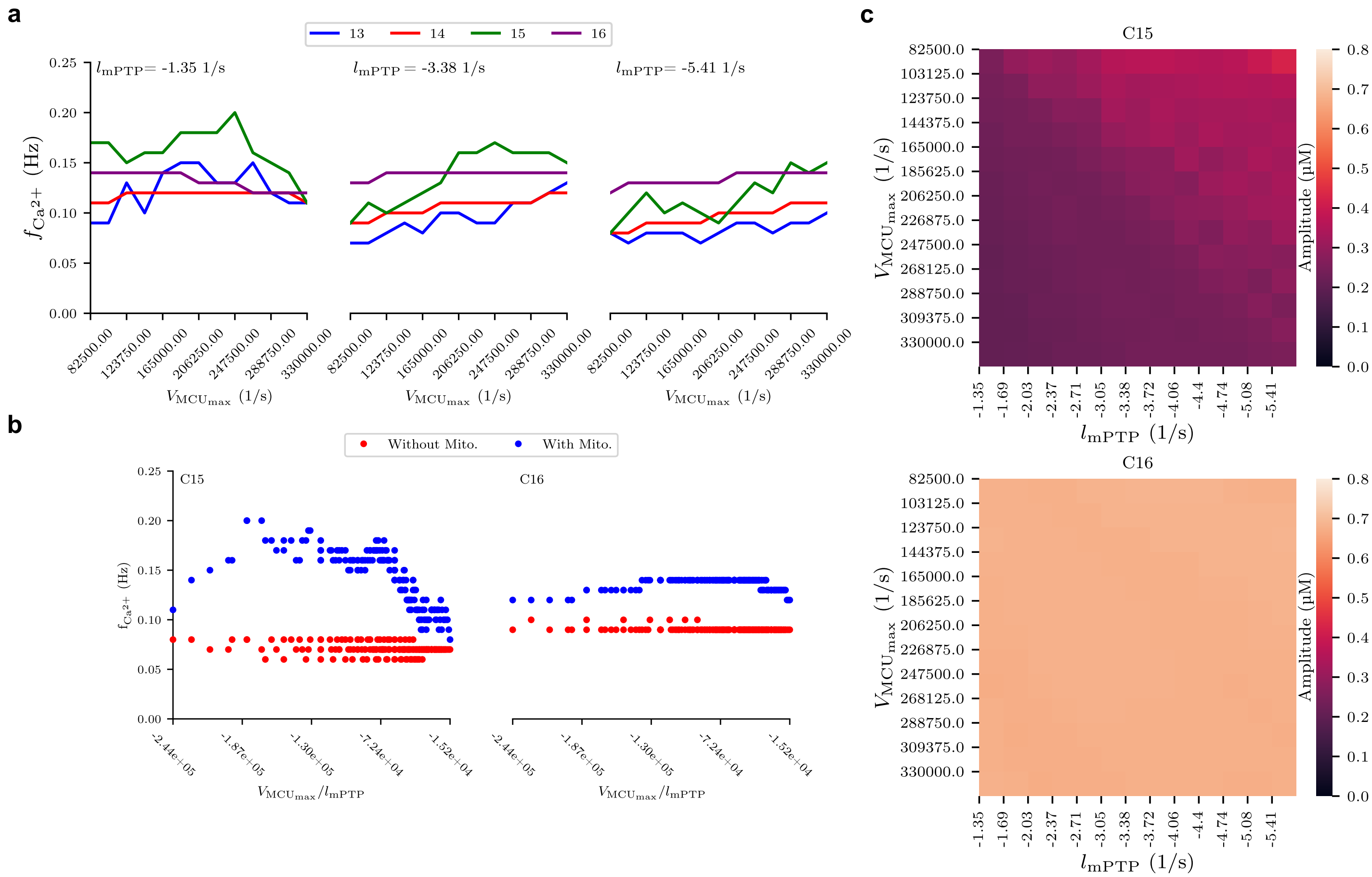

Fig S5: **MCU uptake and mPTP release jointly control Ca^{2+} oscillation frequency.** (**a**) $f_{Ca^{2+}}$ in C13–C16 for varying V_{MCUmax} and three mPTP release rates ($l_{mPTP} = -1.35$, -3.38 or -5.41 s^{-1}). (**b**) $f_{Ca^{2+}}$ in C15 and C16 as a function of the V_{MCUmax}/l_{mPTP} ratio. $f_{Ca^{2+}}$ is reduced for both higher MCU uptake and low mPTP release or low MCU uptake. Higher $f_{Ca^{2+}}$ is achieved at intermediate ratios. (**c**) Average amplitude of Ca^{2+} signals in compartments 15 (with mitochondria) and 16 (without mitochondria).

mediated Ca^{2+} uptake reduced propagation extent similarly in models with and without mitochondria (Fig. **S10**), indicating that the qualitative mitochondrial effect was preserved across the tested range.

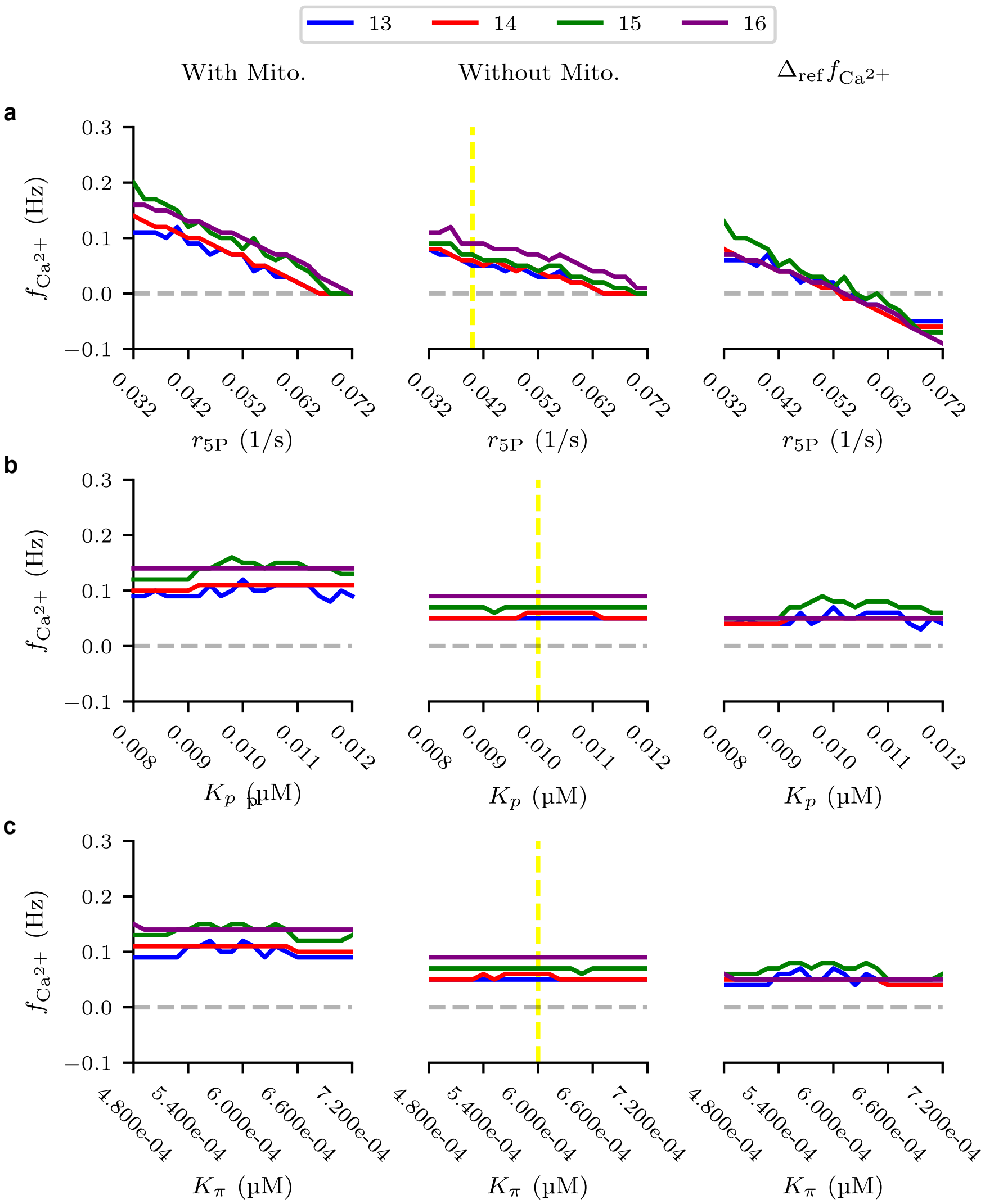

Fig S6: **Sensitivity of $f_{\text{Ca}^{2+}}$ to IP_3 turnover parameters.** Increasing Ca^{2+}-independent IP_3 degradation (r_{5P}) brings $f_{\text{Ca}^{2+}}$ in the mitochondrial model closer to the fixed reference response (**a**), whereas varying the Ca^{2+}-dependent PLCβ inhibition parameter K_p (**b**) or PLCβCa^{2+} affinity K_π (**c**) has little effect. Vertical yellow dashed lines indicate the default parameter values used to define the non-mitochondrial reference response. Differences from reference are calculated according to Eq. (S31). The horizontal gray dashed line indicates $\Delta_{\text{ref}} f_{\text{Ca}^{2+}} = 0$.

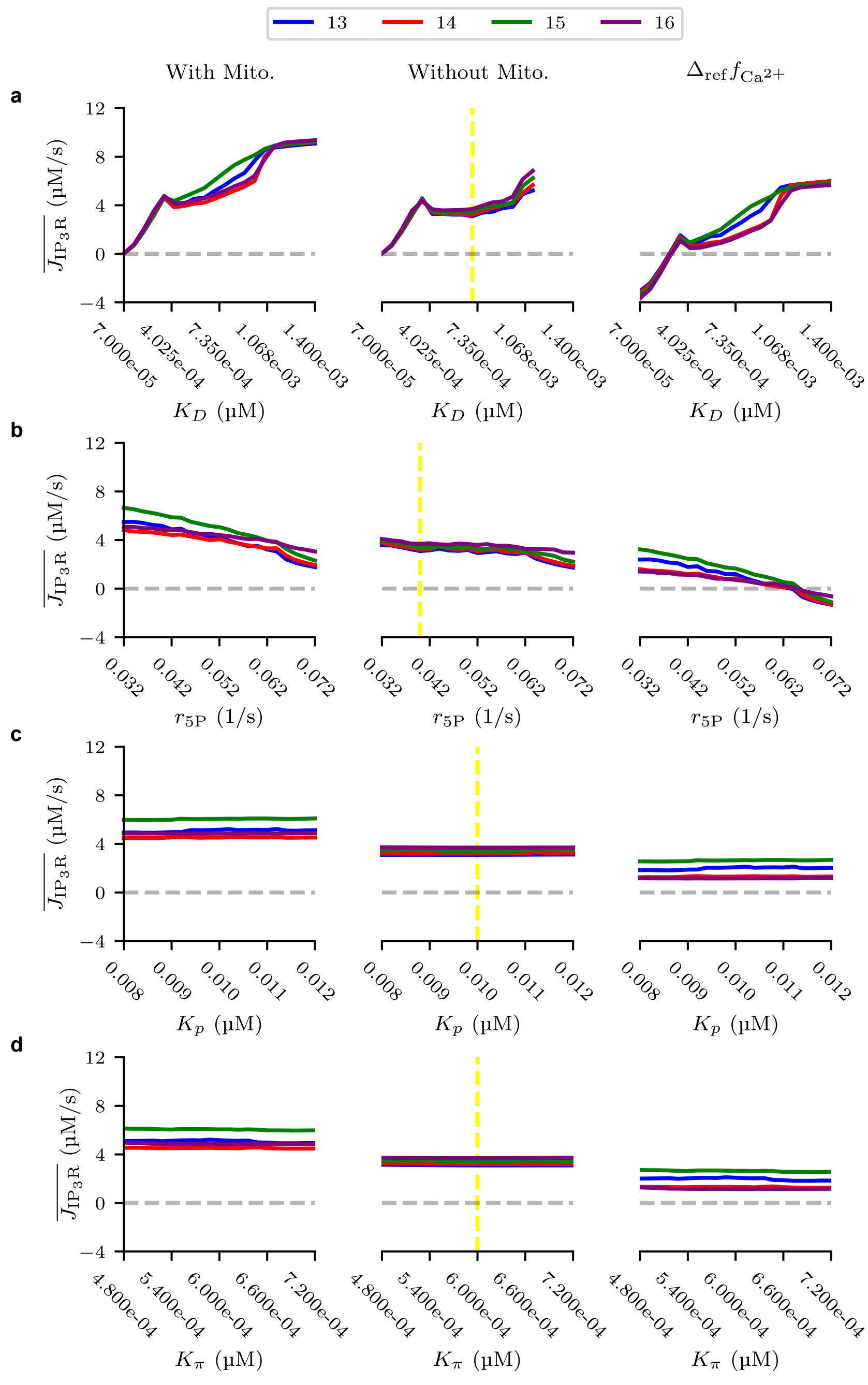

Fig S7: **Mean ER Ca^{2+} efflux via IP_3R ($\overline{J_{IP_3R}}$) as a function of IP_3 turnover parameters.** (**a**) Increasing Ca^{2+} affinity of IP_3-3K (lower K_D) reduces $\overline{J_{IP_3R}}$. (**b**) Increasing r_{5P} also decreases $\overline{J_{IP_3R}}$. (**c**, **d**) K_p and K_π have comparatively small effects. Vertical yellow dashed lines indicate the default parameter values used to define the non-mitochondrial reference response. Differences from reference are calculated according to Eq. (S31); the horizontal gray dashed line indicates zero deviation from the reference.

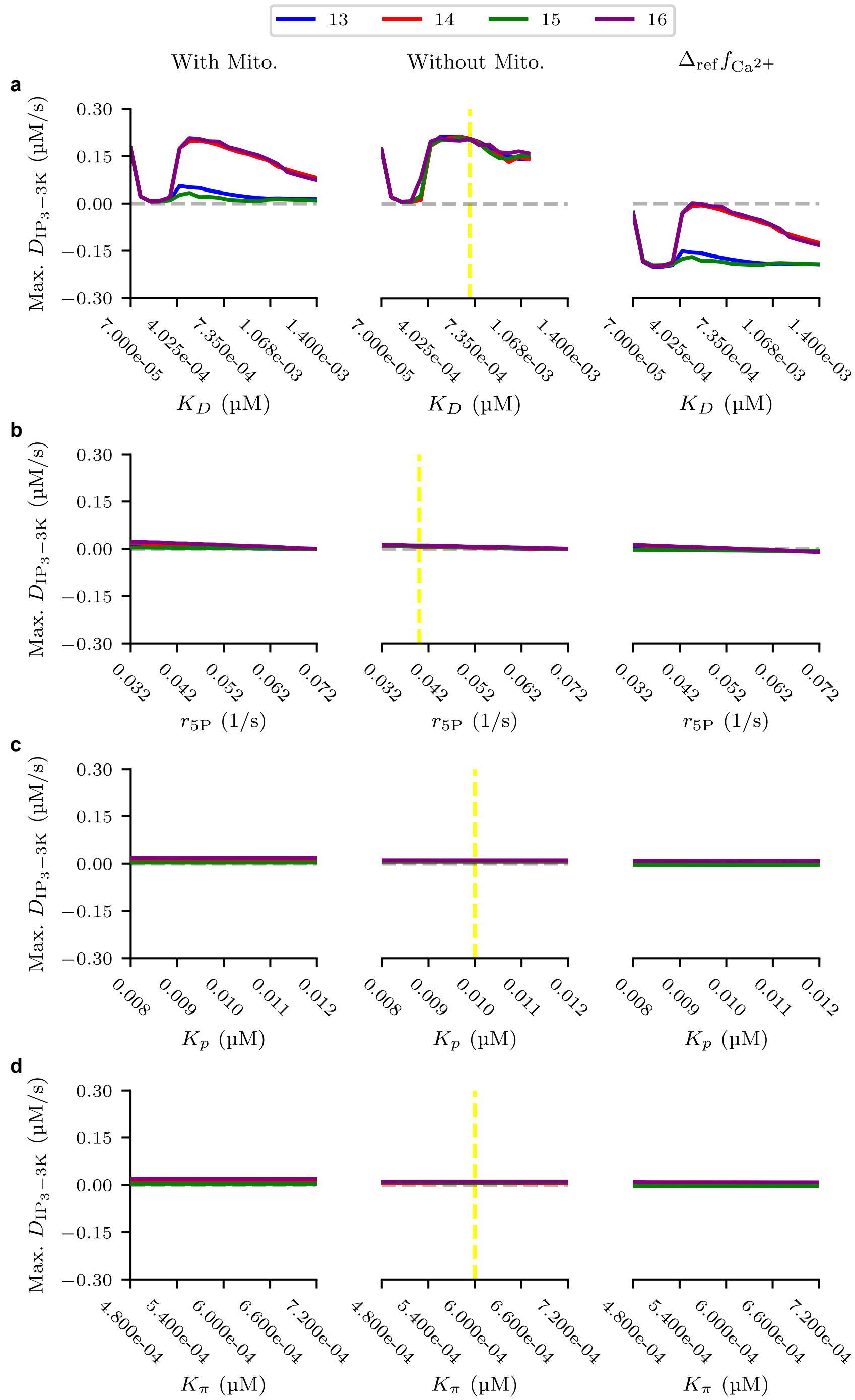

Fig S8: **Maximum Ca^{2+}-dependent degradation (D_{IP_3-3K}) as a function of IP_3 turnover parameters.** (**a**) Increasing the Ca^{2+} affinity of IP_3-3K (lower K_D) initially increases and, at still higher affinity, decreases the maximum degradation rate, depending on the parameter range; (**b-d**) r_{5P}, K_p, and K_π have comparatively small effects. Vertical yellow dashed lines indicate the default parameter values used to define the non-mitochondrial reference response. Differences from reference are calculated according to Eq. (S31); the horizontal gray dashed line indicates zero deviation from the reference.

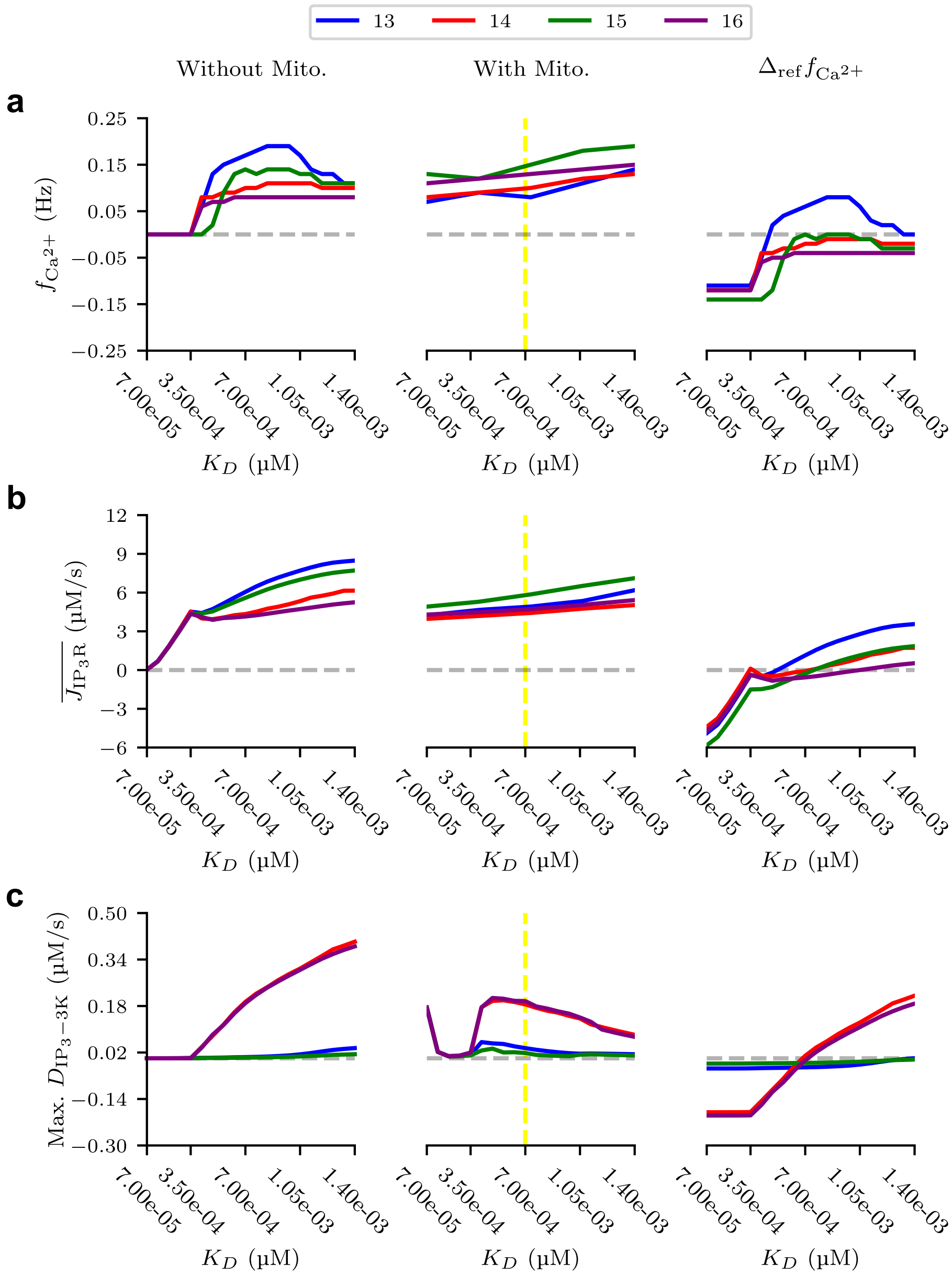

Fig S9: **Inhibition of Ca^{2+}-dependent IP_3 degradation counteracts the reduction in astrocytic response caused by reduced MCU uptake.** (**a**) $f_{\mathrm{Ca}^{2+}}$; (**b**) time-averaged $J_{\mathrm{IP_3R}}$; (**c**) maximum $\mathrm{D}_{\mathrm{IP_3-3K}}$. The vertical yellow dashed line indicates the default K_D value used to define the corresponding reference response of the model without mitochondria. Right-hand panels show deviations from this fixed reference according to Eq. (S31). Values approaching zero indicate restoration toward the reference response.

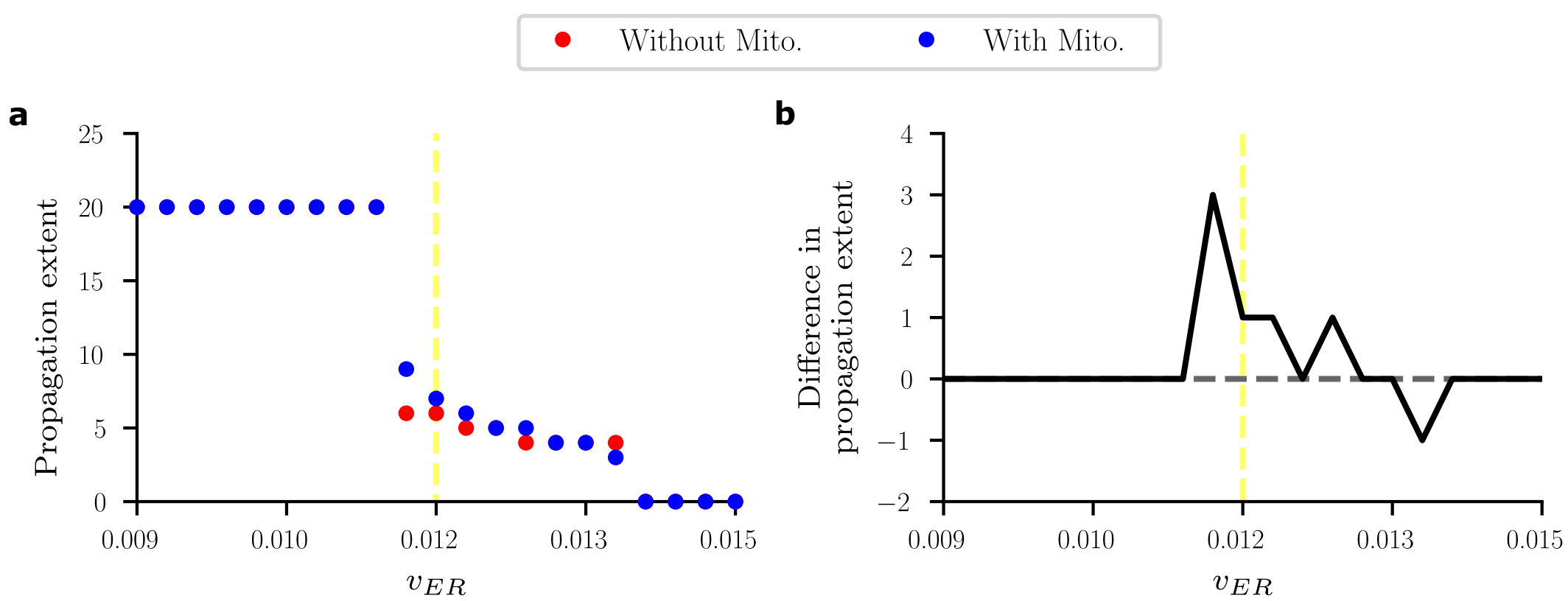

Fig S10: **Effect of the maximum SERCA uptake rate (v_{ER}) on Ca^{2+} propagation. The model was stimulated with glutamate (ν_g = 5 Hz, C18-20).** Increasing SERCA-mediated Ca^{2+} uptake reduces the number of propagated compartments similarly in models with and without mitochondria. The vertical yellow dashed line indicates the default value of v_{ER}. (**a**) Propagation extent, quantified as the number of consecutively activated compartments. (**b**) Pointwise difference in propagation extent, calculated at each v_{ER} as the value in the model with mitochondria minus the value in the model without mitochondria.

5 Comparison with Available Experimental Data

The single-compartment and unipolar models were used to compare selected features of the simulated Ca^{2+} responses with available experimental observations. Because published measurements do not provide direct quantitative counterparts for all variables and spatial measures used in the present model, these comparisons should not be interpreted as a complete validation. Instead, they assess whether selected temporal and amplitude characteristics of the simulated Ca^{2+} signals are consistent with experimentally reported ranges and qualitative effects.

We first compared Ca^{2+} signal frequency ($f_{\mathrm{Ca}^{2+}}$) and duration with values reported experimentally and in previous computational studies [11–19]. Within the glutamatergic and dopaminergic stimulation ranges used in this study, the simulated $f_{\mathrm{Ca}^{2+}}$ generally falls within experimentally reported ranges (Fig**S11a**,**b**,**d**,**e**; Table**S1**). In particular, the frequencies obtained with dopaminergic stimulation are comparable to those measured under pharmacological manipulations of dopaminergic or noradrenergic transmission [18,19]. However, under stronger combined stimulation the model can generate frequencies above 5 signals/min, which are not represented in the experimental data summarized in Table **S1**. The present model also does not generate spontaneous Ca^{2+} signals.

The full width at half maximum (FWHM) and base-width durations of the simulated Ca^{2+} signals are also broadly comparable to experimentally reported values (Fig**S11c**,**f**,**g**; Table**S1**). This comparison should nevertheless be interpreted cautiously because the model directly represents cytosolic Ca^{2+} concentration, whereas experimental signal duration is typically measured with genetically encoded Ca^{2+} indicators (GECIs), which can themselves alter the apparent kinetics of Ca^{2+} transients [20].

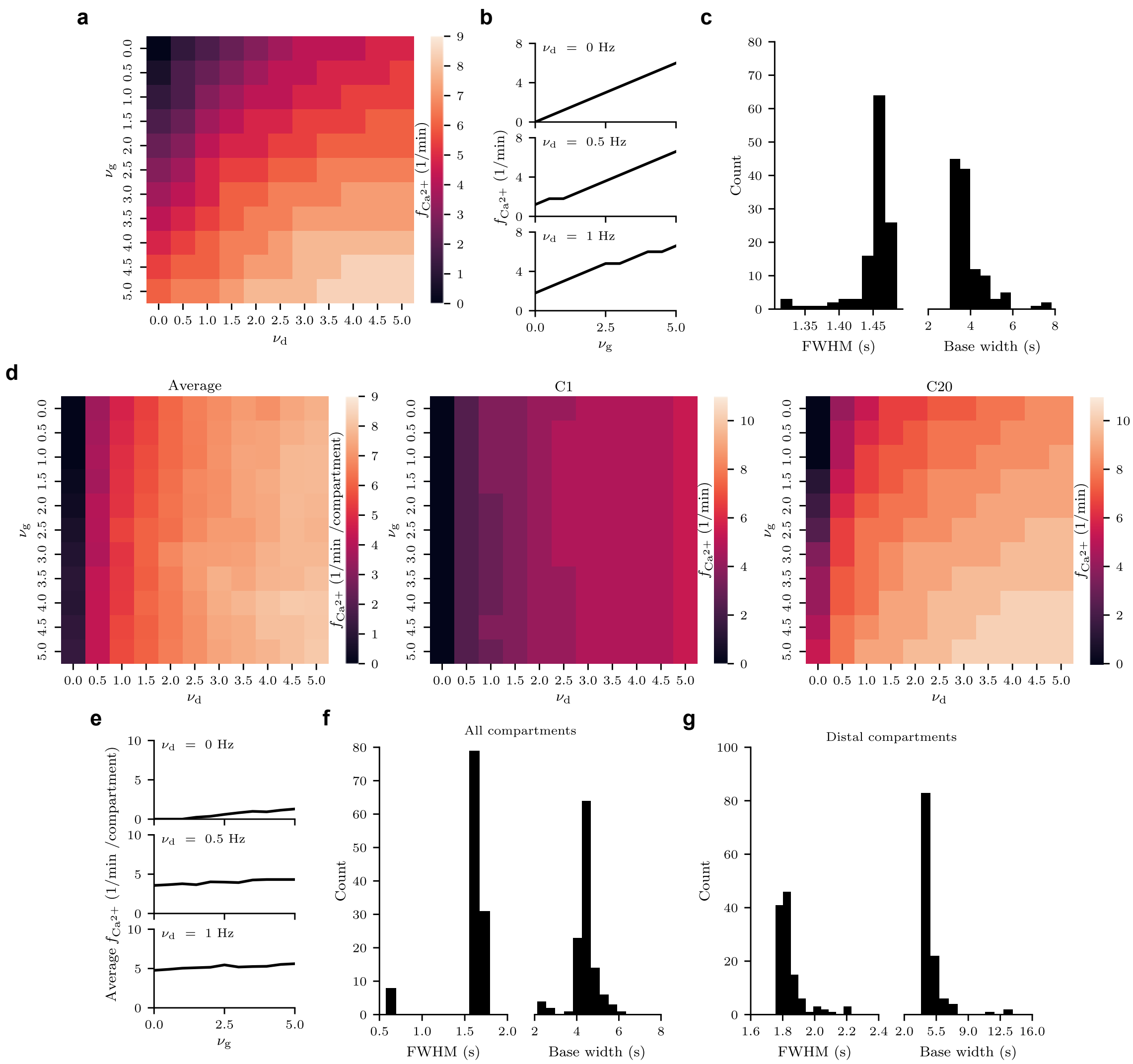

Fig S11: **Frequency and duration of Ca^{2+} signals in the single and unipolar morphological models**. (**a**) Frequency of Ca^{2+} oscillation ($f_{Ca^{2+}}$) in the single compartment model under glutamatergic (input frequency ν_g) and dopaminergic (input frequency ν_d) stimulation. (**b**) ($f_{Ca^{2+}}$) for three frequencies of dopaminergic input ν_d. (**c**) Histogram of full width at half maximum (FWHM) and base width duration of the Ca^{2+} signals in the single compartment model. (**d**) Average, compartment 1 (C1) and compartment 20 (C20) $f_{Ca^{2+}}$ in the unipolar morphology model. **e** Average $f_{Ca^{2+}}$ for three frequencies of dopaminergic input ν_d. Histogram of FWHM and base width durations of Ca^{2+} signals of all compartments (**f**) or distal compartments (**g**) in the unipolar morphology model.

Table S1: **Summary of experimental reports used in the comparison with model results**. Data extracted from published figures using WebPlotDigitizer. *Average calculated from data points. **Average among astrocyte subcellular domains. ***Average calculated from data points. Number of regions of interest (ROIs) not reported.

Study	Species	Brain Region	Preparation	Technique	Stimulus	Duration (s)	$f_{Ca^{2+}}$ (1/min)	Source
Schipke *et al.* (2008)	Mice	Cortex	Slice	Fluo-4	Spontaneous	12.65	—	Fig.3C
Agarwal *et al.* (2017)	Mice	Cortex	Slice	GCaMP3	Spontaneous	9.42	0.64	Fig.3G
Agarwal *et al.* (2017)	Mice	Cortex	Slice	GCaMP3	NE	15.55	0.63	Fig.3H
Agarwal *et al.* (2017)	Mice	Visual cortex	*In vivo*	GCaMP3	Spontaneous	—	0.88	Fig.5I
Bindocci *et al.* (2017)	Mice	Hippocampus	Slice	GCaMP6f	Spontaneous	18.96*	0.552	Fig.3C,4E
Bindocci *et al.* (2017)	Mice	Somatosensory cortex	*In vivo*	GCaMP6f	Spontaneous	12*	1.364	Fig.3C,4E
Stobart *et al.* (2018)	Mice	Somatosensory cortex	*In vivo*	GCaMP6s	Spontaneous	8.97**	0.454*	Fig.1D
Stobart *et al.* (2018)	Mice	Somatosensory cortex	*In vivo*	GCaMP6s	Whisker 90 Hz	6.629	0.987	Fig.3D
Stobart *et al.* (2018)	Mice	Somatosensory cortex	*In vivo*	GCaMP6s	Hindpaw	13.26	0.981	Fig.3D
Göbel *et al.* (2020)	Mice	Cortex	Slice	GCamP6	Spontaneous	37.32	0.440	Fig.5G
Serrat *et al.* (2021)	Mice	Hippocampus	Slice	GCaMP6f	Spontaneous	8.74	8.4***	Fig.5C,E
Serrat *et al.* (2021)	Mice	Hippocampus	Slice	GCaMP6f	Neuronal depolarization	9.04	12.6***	Fig.5C,E
Benoit *et al.* (2025)	Mice	Hippocampus	Slice	GCaMP6f	Spontaneous	2.2	0.492	Fig.4E
Holt *et al.* (2025)	Mice	Nucleus accumbens	*In vivo*	GCaMP6f	Spontaneous	—	0.79	Fig.4C
Holt *et al.* (2025)	Mice	Nucleus accumbens	*In vivo*	GCaMP6f	Cocaine	—	4.44	Fig.4C
Mongrédien *et al.* (2025)	Mice	Nucleus accumbens	Slice	GCaMP6f	Spontaneous	8.29	2.29	Fig.1K,L
Mongrédien *et al.* (2025)	Mice	Nucleus accumbens	Slice	GCaMP6f	Phenylephrine	13.13	2.44	Fig.1K,L

We also compared the qualitative effects of reducing mitochondrial Ca^{2+} uptake and release with experimental perturbations of MCU and mPTP function. In the single-compartment model, reducing MCU uptake increases cytosolic Ca^{2+} amplitude and decreases $f_{Ca^{2+}}$, depending on the degree of reduction (Fig. 10**b**). The increase in amplitude differs from studies reporting little or no change in Ca^{2+} signal amplitude after MCU inhibition [16, 21]. By contrast, reducing mPTP-mediated release decreases Ca^{2+} signal amplitude, qualitatively consistent with the observations reported in [12]. In the unipolar model, the effect depends additionally on whether the analyzed compartment contains mitochondria, illustrating that spatial averaging may obscure local mitochondrial effects.

Direct quantitative validation is currently more limited for mitochondrial Ca^{2+} dynamics and propagation extent. The model predicts specific mitochondrial Ca^{2+} transients associated with cytosolic Ca^{2+} signals, but we have not identified experimental data obtained under sufficiently comparable stimulation conditions to quantitatively constrain their amplitude and time course. Likewise, the model defines the propagation extent as the number of consecutively activated compartments, a quantity that does not have a direct experimental counterpart. Experimental work showing that mitochondria can reduce the velocity of intracellular Ca^{2+} propagation [22] is qualitatively consistent with the suppressive effect predicted here under weak stimulation, but it does not constitute a quantitative validation of the model's propagation measure.

Thus, the comparisons above indicate that several temporal characteristics of the simulated cytosolic Ca^{2+} responses are compatible with experimental observations, while also revealing discrepancies in some perturbation responses. The predicted context-dependent control of Ca^{2+} propagation by mitochondria, particularly at branch points, remains an experimentally testable prediction. Simultaneous imaging of cytosolic and mitochondrial Ca^{2+} during spatially localized synaptic-like stimulation, with and without neuromodulatory input, would provide a direct way to test this mechanism.

6 Additional Morphological and Mechanistic Tests

6.1 Spatial distribution of glutamatergic inputs and mitochondria

Our standard simulations place glutamatergic inputs in distal compartments and mitochondria in alternating compartments. To determine whether the enhancing and gating effects depended on this particular spatial arrangement, we performed additional simulations of the unipolar morphology in which glutamatergic input was restricted to a single compartment and the position of a single mitochondrion was systematically varied. We quantified propagation extent and $f_{Ca^{2+}}$ as functions of the relative positions of the input and mitochondrion (Figs. **S12–S14**).

These simulations showed that similar enhancing and gating effects could occur when the glutamatergic input and mitochondria were positioned away from the distal region, indicating that the qualitative effect does not depend exclusively on the standard distal-input configuration.

6.2 Mitochondrial distribution and morphology

We next tested whether the main effects depended on the standard assumption that mitochondria occupy alternating compartments. Alternative mitochondrial distributions and sizes altered quantitative features of the Ca^{2+} response, including signal frequency and spatial distribution, but

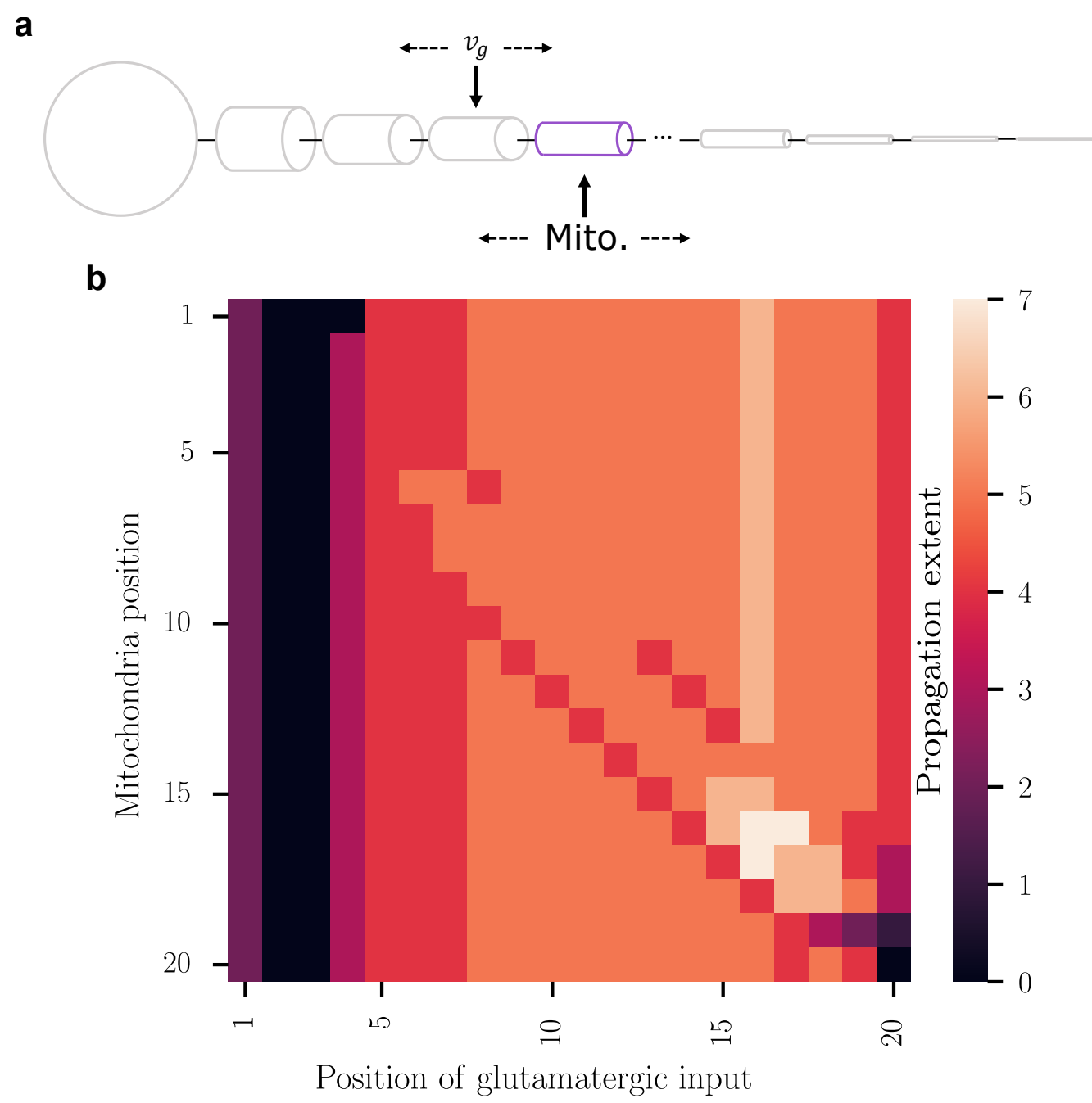

Fig S12: **Propagation extent of Ca^{2+} changing the position of both mitochondria and glutamatergic input (ν_g = 5 Hz).** In this test, only one single compartment has mitochondria. Propagation extent calculated as the number of compartments activated from the point of stimulation. In the distal compartments (C18-20), mitochondria close to the stimulation point reduce the propagation extent. In C17, mitochondria closer to the stimulation point increase the propagation extent. In C8-15, mitochondria closer to the distal regions prevents the Ca^{2+} propagating toward the mitochondria. In the intermediate compartments C5-7, mitochondria closer to the stimulation point increase the propagation extent. In proximal the somatic C1 and proximal compartments C2-4 there is no interaction between mitochondria and the stimulation point.

preserved the main qualitative conclusion that mitochondria can either restrict or facilitate Ca^{2+} signaling depending on stimulation conditions (Figs. **S15**–**S16**).

6.3 Mitochondria–ER contact sites

Mitochondria–ER contact sites (MERCS) can promote direct transfer of Ca^{2+} from the ER to mitochondria [7,16,23]. Although MERCS were not explicitly represented in our standard model, we examined their possible effect by partitioning ER Ca^{2+} release into a fraction α_{MERC} entering the cytosol and a fraction $1 - \alpha_{\mathrm{MERC}}$ transferred directly to mitochondria. This modification preserved the frequency and spatial location of the astrocytic Ca^{2+} response but altered the timing of cytosolic and mitochondrial Ca^{2+} signals (Fig. **S17**). Thus, explicit ER-to-mitochondria transfer did not alter the main qualitative conclusions but may be important for reproducing the temporal relationship between cytosolic and mitochondrial Ca^{2+} dynamics.

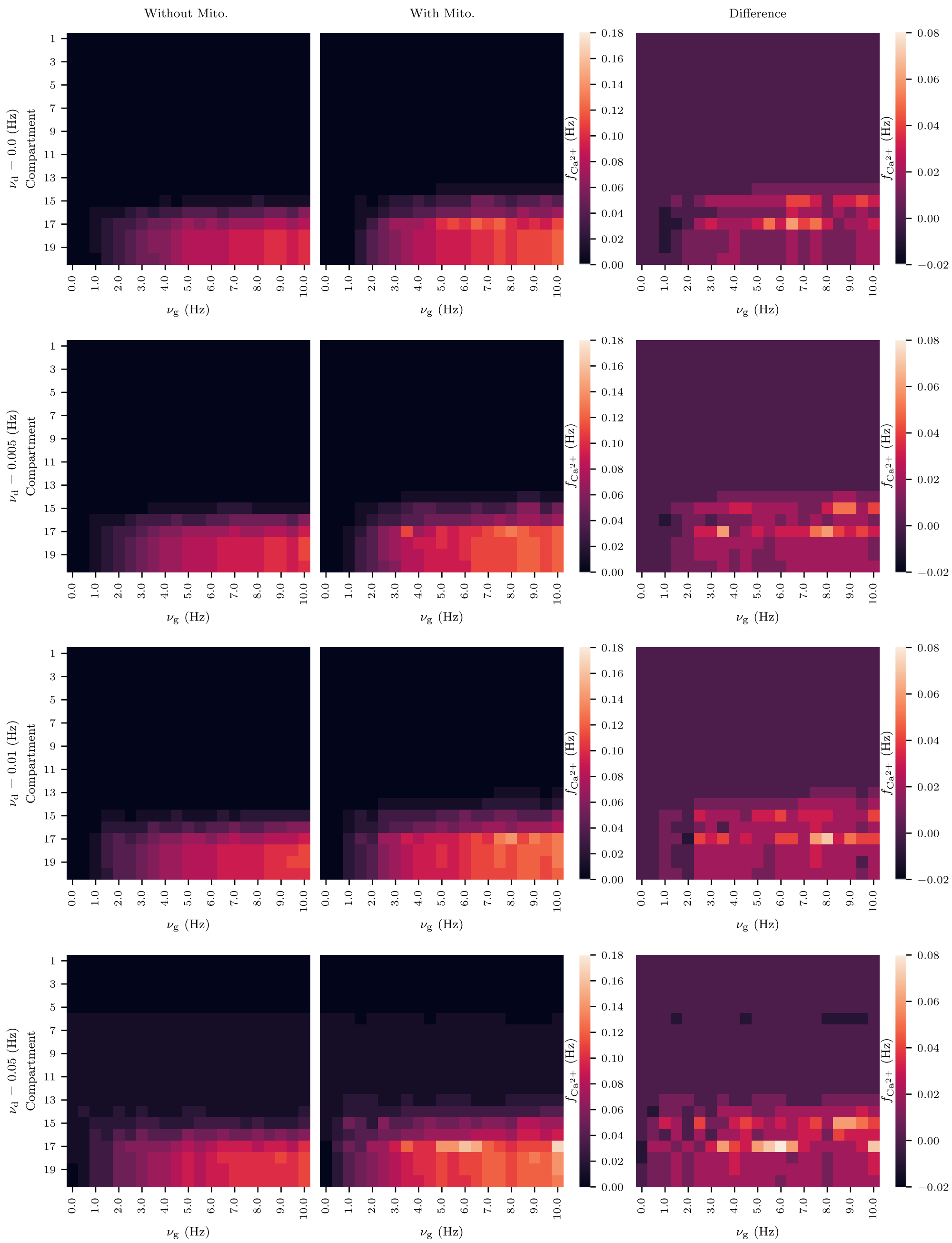

Fig S13: $f_{Ca^{2+}}$ **of all compartments in the unipolar morphology with glutamatergic and/or dopaminergic inputs**. First column: model without mitochondria. Second column: model with mitochondria. Third column: difference between the model with mitochondria and the model without mitochondria. Without dopamine, mitochondria reduce the response for low ν_g ($<$ 2 Hz) compared to the model without mitochondria. Mitochondria enhance the model response With higher glutamatergic input or with dopaminergic stimulation.

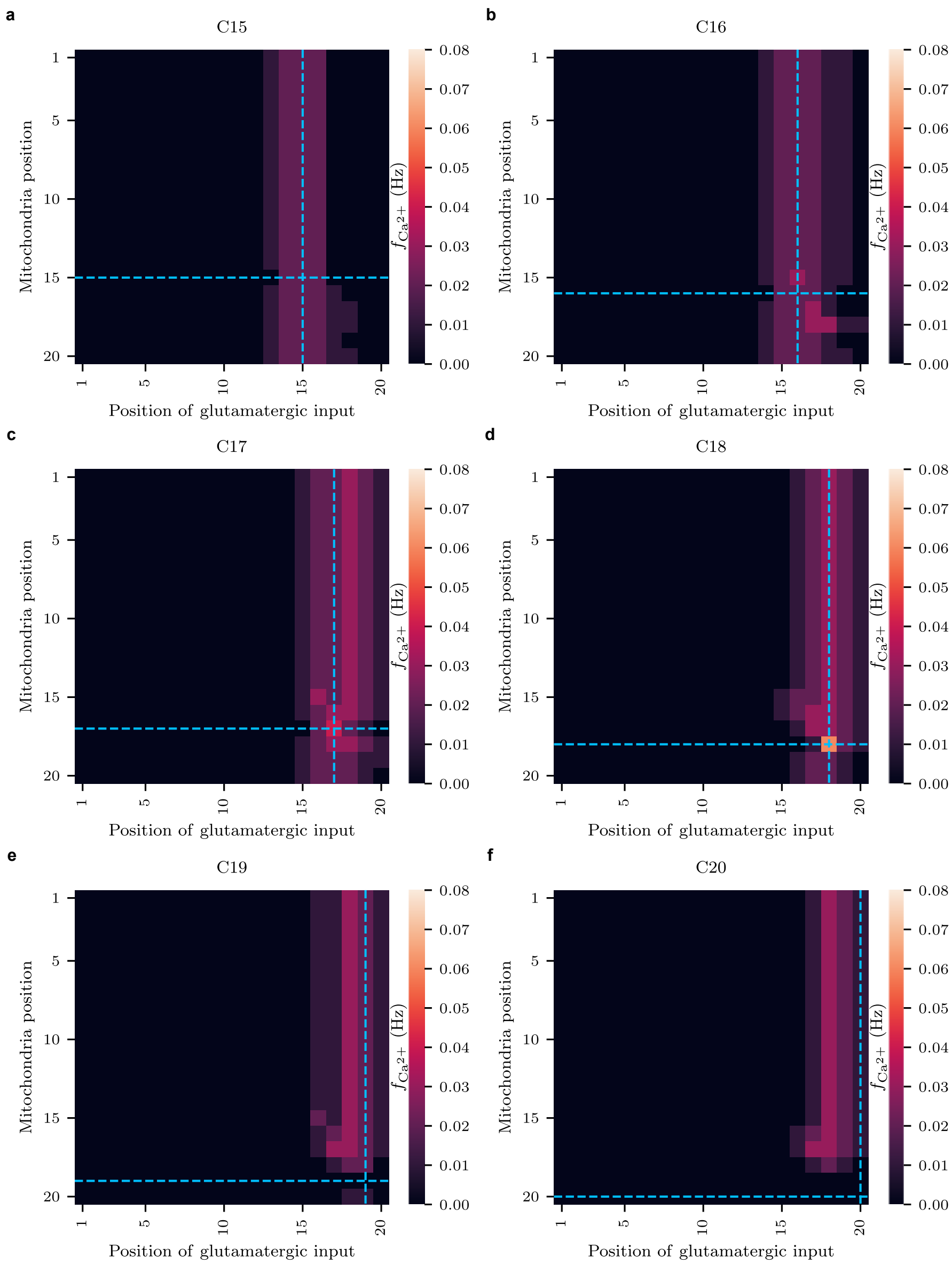

Fig S14: **Co-localization of mitochondria and glutamatergic input modulate astrocyte response in distal compartments**. In this test, only one single compartment has mitochondria (indicated in the vertical axis). Position of glutamatergic input indicated in the horizontal axis (ν_g = 5 Hz.) $f_{\mathrm{Ca}^{2+}}$ of compartments (**a**) C15, (**b**) C16, (**c**) C17, (**d**) C18, (**e**) C19 and (**f**) C20. No difference is observed for the co-localization of mitochondria and glutamatergic input in compartments C15 and C16 (**a** and **b**). Co-localization in compartments C17-19 increase the $f_{\mathrm{Ca}^{2+}}$ of these compartments. In contrast, co-localization in compartments C19 and C20 reduces the $f_{\mathrm{Ca}^{2+}}$ of these compartments. Positioning the mitochondria away from the distal compartments increases the response in compartments C19 and C20.

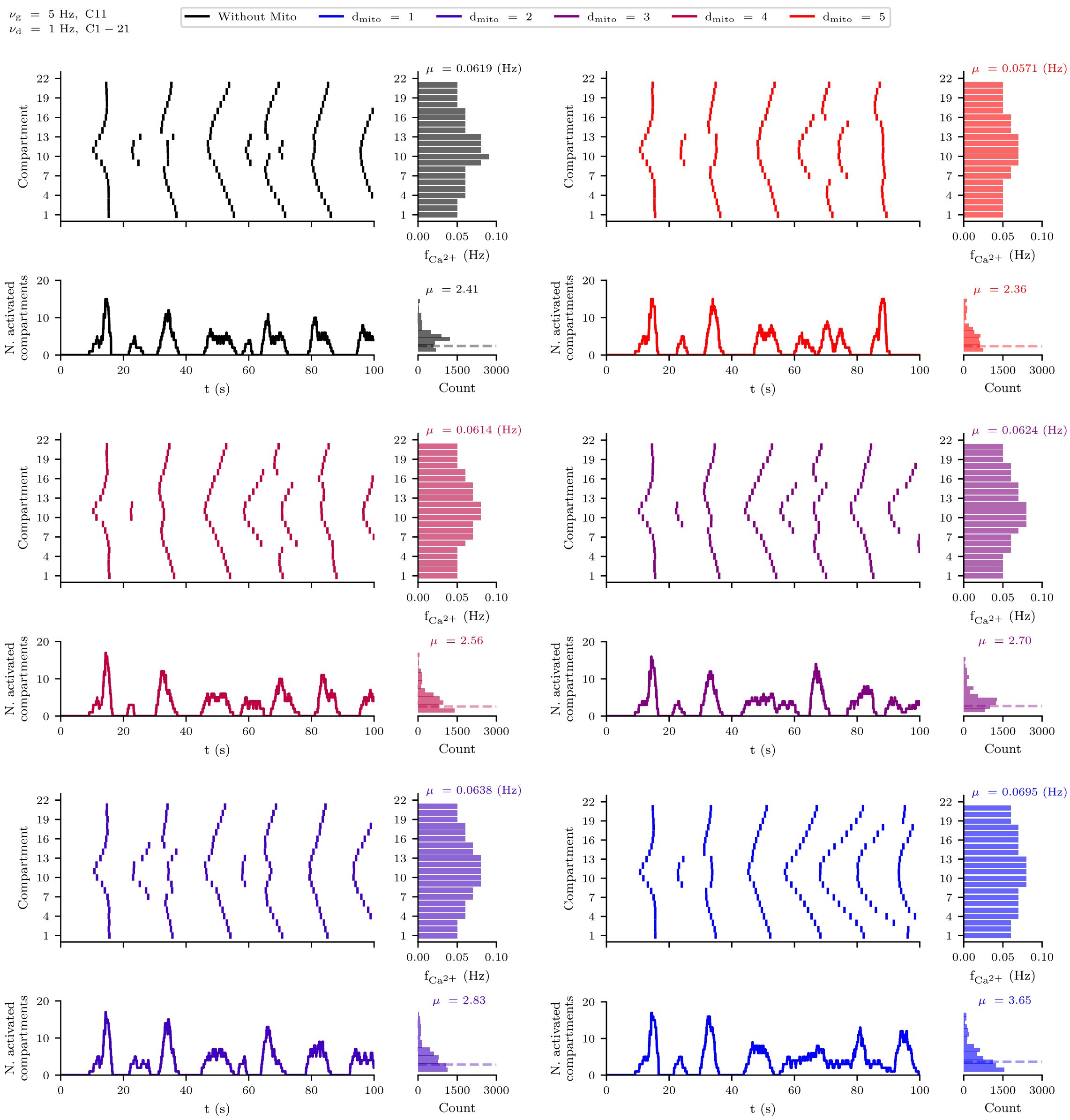

Fig S15: **The distribution of mitochondria regulates the enhancing and gating effect strength**. Same-size morphological model (21 compartments, ϕ = 2 µm; Tables S5 and S6 in S2 Text) with different distributions of mitochondria under glutamatergic (ν_g = 5 Hz, C11, middle compartment) and dopaminergic (ν_d = 1 Hz, C1-21) stimulation. d_{mit} indicates the distance between compartments with mitochondria. Compartment 1 has always mitochondria. In each panel: Raster plot (upper left), $f_{Ca^{2+}}$ and average frequency (upper right), number of activated compartments (bottom left) and histogram with the number of activated compartments and average number of activated compartments per time step (bottom right).

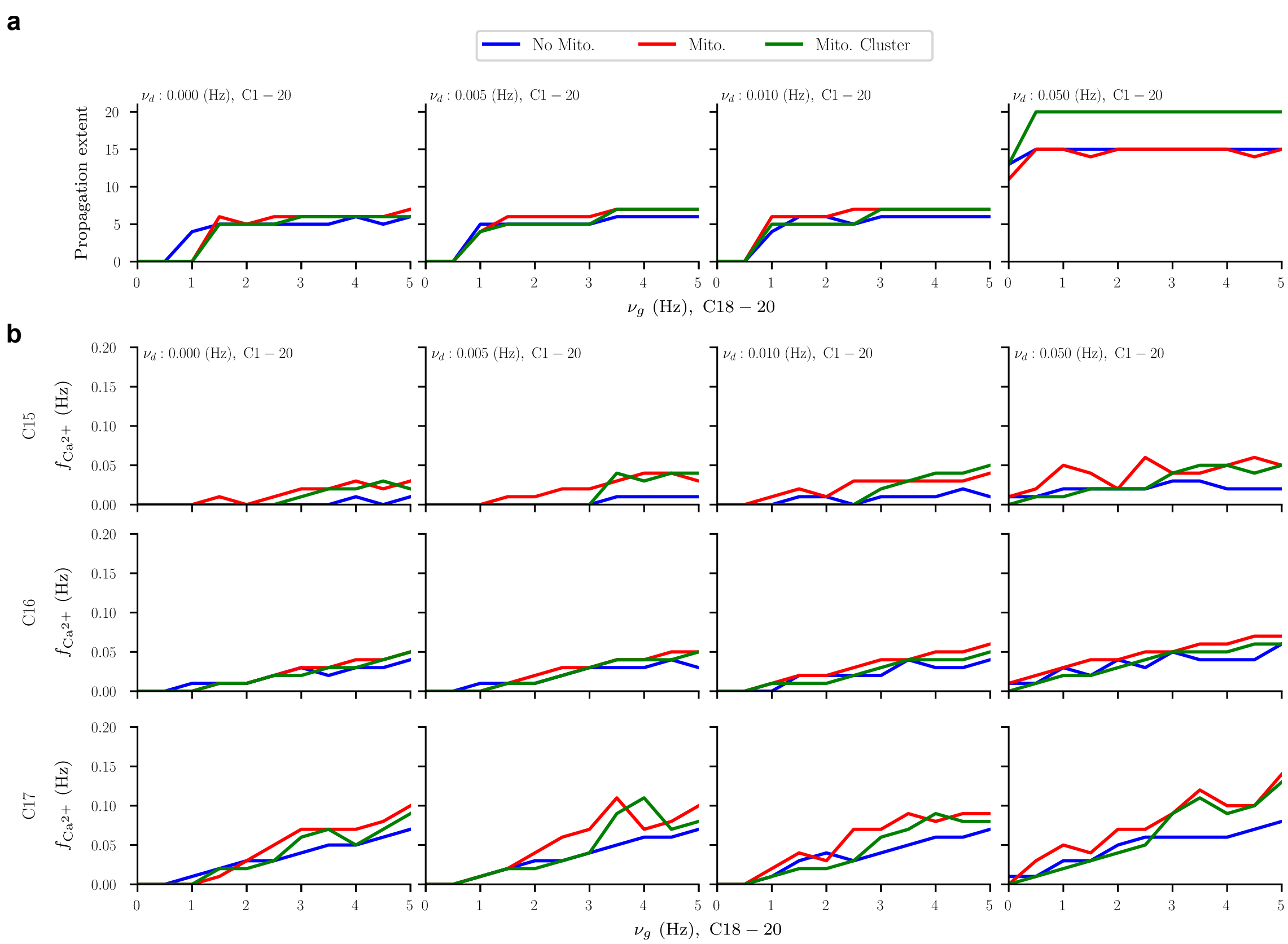

Fig S16: **Clusters of mitochondria strengthen the mitochondrial gating effect on the propagation extent and $f_{Ca^{2+}}$.** The number of mitochondria in the unipolar morphology was increased to match the described in [21] (Table S7 in S2 Text). Astrocyte stimulated with different dopaminergic (ν_d, C1-20) and glutamatergic (ν_g, C18-20) input frequencies. (**a**) Propagation extent. (**b**) $f_{Ca^{2+}}$ of compartments C15, C16 and C17.

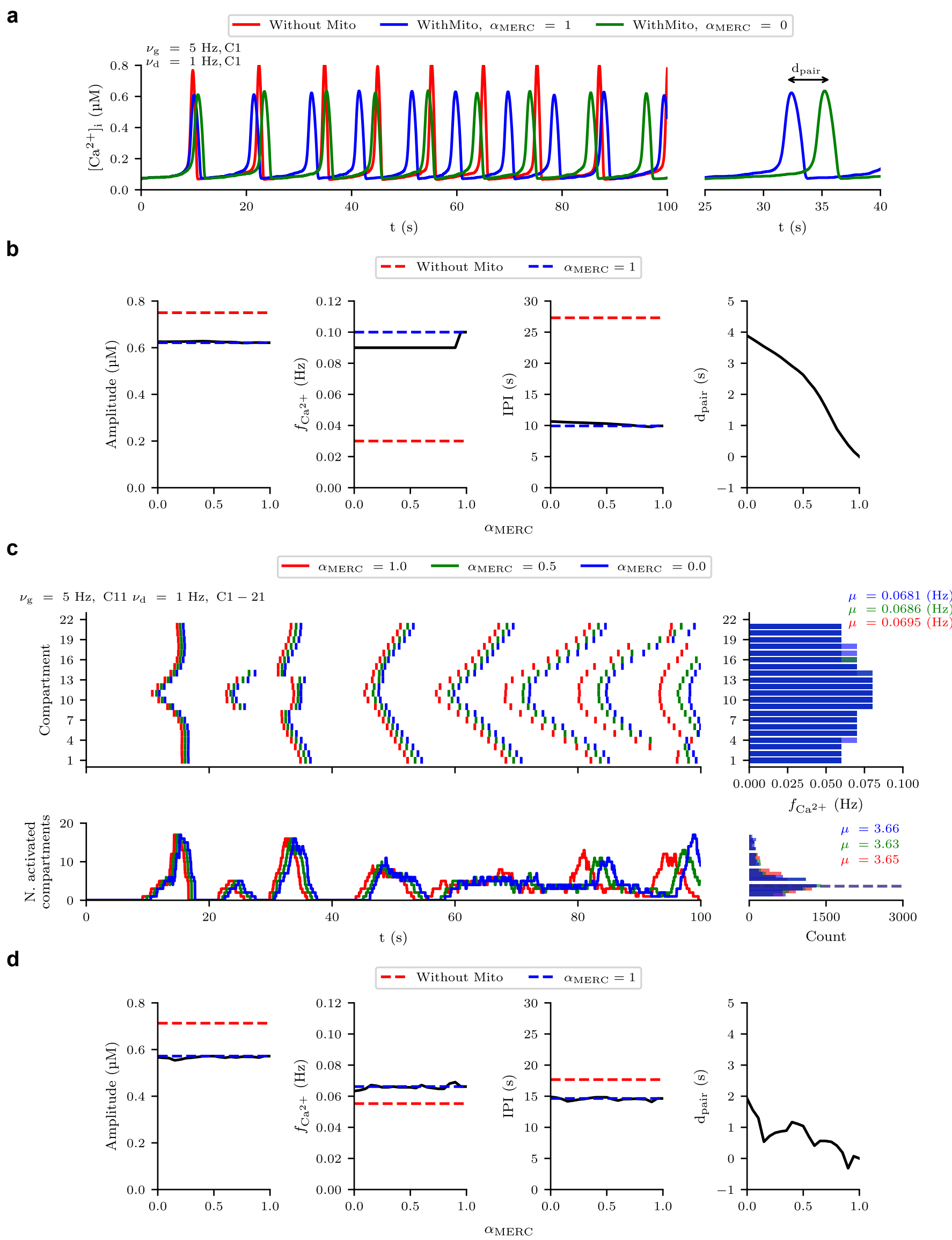

Fig S17: **Mitochondria–ER contact sites (MERCS) alter the timing of Ca^{2+} signals while preserving amplitude, frequency, and inter-peak intervals (IPI).** ER Ca^{2+} release was partitioned between the cytosol and mitochondria according to α_{MERC}, with $\alpha_{\mathrm{MERC}} = 1$ corresponding to complete transfer to the cytosol. (**a**, **b**) Single-compartment model ($\nu_g = 5$ Hz, $\nu_d = 1$ Hz). (**a**) Intracellular Ca^{2+} traces (left) and intervals between pairs of Ca^{2+} signals (right) for complete transfer to the cytosol (blue) or mitochondria (green). (**b**) Average Ca^{2+} signal amplitude, $f_{\mathrm{Ca}^{2+}}$, IPI, and interval between pairs of Ca^{2+} peaks. Direct ER-to-mitochondria transfer delays Ca^{2+} signals. (**c**, **d**) Same-size compartmental model (21 compartments with mitochondria; $\nu_g = 5$ Hz, C11; $\nu_d = 1$ Hz, C1–21). (**c**) Raster plot, $f_{\mathrm{Ca}^{2+}}$, and number of activated compartments. (**d**) Average amplitude, $f_{\mathrm{Ca}^{2+}}$, IPI, and interval between pairs of Ca^{2+} peaks. Averages were calculated across time and compartments.

7 Computational and Numerical Methods

The system of differential equations was solved using the Euler method with timestep $dt = 0.01$ ms. To reduce memory usage, simulation outputs were sampled at a rate of 0.01 Hz. Numerical routines were implemented in C++, and data analysis performed in Python (v3.12.7) using Matplotlib (v3.9.2) and NumPy (v1.26.4). The code of the model is available on GitHub at: `https://github.com/ThiagoTakechi/AstrocyteMitochondriaModel.git`.

References

1. De Pittà M, Goldberg M, Volman V, Berry H, Ben-Jacob E. Glutamate regulation of calcium and IP 3 oscillating and pulsating dynamics in astrocytes. J Biol Phys. 2009;35:383-411.

2. De Pittà M, Ben-Jacob E, Berry H. G protein-coupled receptor-mediated calcium signaling in astrocytes. Computational glioscience. 2019:115-50.

3. Liu J, Wang F, Huang C, Long LH, Wu WN, Cai F, et al. Activation of phosphatidylinositol-linked novel D 1 dopamine receptor contributes to the calcium mobilization in cultured rat prefrontal cortical astrocytes. Cell Mol Neurobiol. 2009;29:317-28.

4. Pittolo S, Yokoyama S, Willoughby DD, Taylor CR, Reitman ME, Tse V, et al. Dopamine activates astrocytes in prefrontal cortex via α1-adrenergic receptors. Cell Rep. 2022;40(13).

5. Bezerra TO, Roque AC. Dopamine facilitates the response to glutamatergic inputs in astrocyte cell models. PLOS Comput Biol. 2024;20(12):e1012688.

6. Goldberg M, De Pittà M, Volman V, Berry H, Ben-Jacob E. Nonlinear gap junctions enable long-distance propagation of pulsating calcium waves in astrocyte networks. PLoS Comput Biol. 2010;6(8):e1000909.

7. Salmon CK, Syed TA, Kacerovsky JB, Alivodej N, Schober AL, Sloan TF, et al. Organizing principles of astrocytic nanoarchitecture in the mouse cerebral cortex. Curr Biol. 2023;33(5):957-72.

8. Friel D, Tsien R. An FCCP-sensitive Ca2+ store in bullfrog sympathetic neurons and its participation in stimulus-evoked changes in [Ca2+] i. J Neurosci. 1994;14(7):4007-24.

9. Pivovarova NB, Hongpaisan J, Andrews SB, Friel DD. Depolarization-induced mitochondrial Ca accumulation in sympathetic neurons: spatial and temporal characteristics. J Neurosci. 1999;19(15):6372-84.

10. Gordleeva SY, Ermolaeva AV, Kastalskiy IA, Kazantsev VB. Astrocyte as spatiotemporal integrating detector of neuronal activity. Front Physiol. 2019;10:294.

11. Schipke CG, Heidemann A, Skupin A, Peters O, Falcke M, Kettenmann H. Temperature and nitric oxide control spontaneous calcium transients in astrocytes. Cell Calcium. 2008;43(3):285-95.

12. Agarwal A, Wu PH, Hughes EG, Fukaya M, Tischfield MA, Langseth AJ, et al. Transient opening of the mitochondrial permeability transition pore induces microdomain calcium transients in astrocyte processes. Neuron. 2017;93(3):587-605.

13. Bindocci E, Savtchouk I, Liaudet N, Becker D, Carriero G, Volterra A. Three-dimensional Ca2+ imaging advances understanding of astrocyte biology. Science. 2017;356(6339):eaai8185.

14. Stobart JL, Ferrari KD, Barrett MJ, Stobart MJ, Looser ZJ, Saab AS, et al. Long-term in vivo calcium imaging of astrocytes reveals distinct cellular compartment responses to sensory stimulation. Cerebral Cortex. 2018;28(1):184-98.

15. Göbel J, Engelhardt E, Pelzer P, Sakthivelu V, Jahn HM, Jevtic M, et al. Mitochondria-endoplasmic reticulum contacts in reactive astrocytes promote vascular remodeling. Cell Metabolism. 2020;31(4):791-808.

16. Serrat R, Covelo A, Kouskoff V, Delcasso S, Ruiz-Calvo A, Chenouard N, et al. Astroglial ER-mitochondria calcium transfer mediates endocannabinoid-dependent synaptic integration. Cell Rep. 2021;37(12).

17. Benoit L, Hristovska I, Liaudet N, Jouneau PH, Fertin A, de Ceglia R, et al. Astrocytes functionally integrate multiple synapses via specialized leaflet domains. Cell. 2025;188(23):6453-72.

18. Holt LM, Minier-Toribio A, Futamura R, Browne CJ, Martínez-Rivera FJ, Markovic T, et al. Astrocytic CREB regulates transcriptomic, neuronal, and behavioral responses to cocaine. Science Advances. 2025;11(51):eaea2419.

19. Mongrédien R, Anesio A, Fernandes GJ, Eagle AL, Maldera S, Pham C, et al. Astrocytes control cocaine-induced synaptic plasticity and reward through the matricellular protein hevin. Biological psychiatry. 2025.

20. Denizot A, Arizono M, Nägerl UV, Soula H, Berry H. Simulation of calcium signaling in fine astrocytic processes: Effect of spatial properties on spontaneous activity. PLoS computational biology. 2019;15(8):e1006795.

21. Huntington TE, Srinivasan R. Astrocytic mitochondria in adult mouse brain slices show spontaneous calcium influx events with unique properties. Cell Calcium. 2021;96:102383.

22. Boitier E, Rea R, Duchen MR. Mitochondria exert a negative feedback on the propagation of intracellular Ca2+ waves in rat cortical astrocytes. The Journal of cell biology. 1999;145(4):795-808.

23. Cabral-Costa JV, Kowaltowski AJ. Mitochondrial Ca2+ handling as a cell signaling hub: lessons from astrocyte function. Essays Biochem. 2023;67(1):63-75.
