# Supplementary material for: Context-Dependent modulation of astrocytic Ca2+ signals by mitochondria: a computational study
Source: J Comput Neurosci. 2026 Sep 25;54(4):743–62. doi: 10.1007/s10827-026-00956-3 (PMC13710611; doi:10.1007/s10827-026-00956-3)
Supplement: Supplementary file 2 [file 10827_2026_956_MOESM2_ESM.pdf]

Context-Dependent Modulation of Astrocytic Ca^{2+} Signals by Mitochondria - A Computational Study
Online Resource 2: Morphologies

Thiago Ohno Bezerra[1], Antonio C. Roque[1,*]

[1] Department of Physics, School of Philosophy, Sciences and Letters of Ribeirão Preto, University of São Paulo, Bandeirantes avenue, Ribeirão Preto, 14040-900, São Paulo, Brazil
* Corresponding author. E-mail: antonior@usp.br;

Table S2: **Summary of the compartmental parameters used for the single compartment model.** Data specifying surface area, volume, cytosol–ER and cytosol–mitochondria volume fractions, and intercompartmental connectivity.

**C**	**S (μm^2)**	**Vol (μm^3)**	**r_{ER}**	**r_m**	**Connections**
1	1.26E+01	1.26E+01	1.50E-01	1.42E-03	—

Table S3: **Summary of the compartmental parameters used for the unipolar morphology.** Data specifying surface area, volume, cytosol–ER and cytosol–mitochondria volume fractions, mitochondrial distribution (1 = present, 0 = absent), and intercompartmental connectivity.

**C**	**S (μm^2)**	**Vol (μm^3)**	$\mathbf{r_{ER}}$	$\mathbf{r_m}$	**Mito.**	**Connections**
1	5.03E+03	3.35E+04	1.50E-01	5.05E-07	1	2
2	1.26E+01	1.26E+01	1.50E-01	—	0	1&3
3	1.19E+01	1.13E+01	1.50E-01	1.61E-03	1	2&4
4	1.12E+01	1.00E+01	1.50E-01	—	0	3&5
5	1.05E+01	8.84E+00	1.50E-01	2.13E-03	1	4&6
6	9.86E+00	7.74E+00	1.49E-01	—	0	5&7
7	9.18E+00	6.71E+00	1.49E-01	2.96E-03	1	6&8
8	8.51E+00	5.76E+00	1.49E-01	—	0	7&9
9	7.83E+00	4.88E+00	1.49E-01	4.36E-03	1	8&10
10	7.16E+00	4.07E+00	1.49E-01	—	0	9&11
11	6.48E+00	3.34E+00	1.48E-01	6.87E-03	1	10&12
12	5.80E+00	2.68E+00	1.48E-01	—	0	11&13
13	5.13E+00	2.09E+00	1.47E-01	1.18E-02	1	12&14
14	4.45E+00	1.58E+00	1.46E-01	—	0	13&15
15	3.77E+00	1.13E+00	1.45E-01	2.32E-02	1	14&16
16	3.10E+00	7.64E-01	1.42E-01	—	0	15&17
17	2.42E+00	4.67E-01	1.35E-01	5.90E-02	1	16&18
18	1.75E+00	2.42E-01	1.20E-01	—	0	17&19
19	1.07E+00	9.09E-02	7.46E-02	—	0	18&20
20	3.93E-01	1.23E-02	1.03E-04	—	0	19

Table S4: **Summary of the compartmental parameters used for the bifurcated-terminal morphology with mitochondria at the bifurcation point.** Data specifying surface area, volume, cytosol–ER and cytosol–mitochondria volume fractions, mitochondrial distribution (1 = present, 0 = absent), and intercompartmental connectivity.

C	S (μm²)	Vol (μm³)	r_{ER}	r_m	Mito.	Connections
1	5026.5482	33510.3216	1.50E-01	5.05E-07	1	2
2	12.5664	12.5664	1.50E-01	1.42E-03	0	1&3
3	11.8901	11.2501	1.50E-01	1.61E-03	1	2&4
4	11.2137	10.0067	1.50E-01	1.84E-03	0	3&5
5	10.5374	8.8361	1.50E-01	2.13E-03	1	4&6
6	9.8611	7.7382	1.49E-01	2.49E-03	0	5&7
7	9.1848	6.7132	1.49E-01	2.96E-03	1	6&8
8	8.5085	5.7610	1.49E-01	3.56E-03	0	7&9
9	7.8322	4.8815	1.49E-01	4.36E-03	1	8&10
10	7.1558	4.0749	1.49E-01	5.42E-03	0	9&11
11	6.4795	3.3410	1.48E-01	6.87E-03	1	10&12
12	5.8032	2.6800	1.48E-01	8.89E-03	0	11&13
13	5.1269	2.0917	1.47E-01	1.18E-02	1	12&14
14	4.4506	1.5763	1.46E-01	1.62E-02	0	13&15
15	3.7743	1.1336	1.45E-01	2.32E-02	1	14&16
16	3.0980	0.7637	1.42E-01	3.53E-02	0	15&17
17	2.4216	0.4667	1.35E-01	5.90E-02	1	16&18&21
18	1.7453	0.2424	1.20E-01	1.16E-01	0	17&19
19	1.0690	0.0909	7.46E-02	3.12E-01	0	18&20
20	0.3927	0.0123	1.03E-04	2.34E+00	0	19
21	1.7453	0.2424	1.20E-01	1.16E-01	0	17&22
22	1.0690	0.0909	7.46E-02	3.12E-01	0	21&23
23	0.3927	0.0123	1.03E-04	2.34E+00	0	22

Table S5: **Summary of the compartmental parameters used for the bifurcated-terminal morphology without mitochondria at the bifurcation point.** Data specifying surface area, volume, cytosol–ER and cytosol–mitochondria volume fractions, mitochondrial distribution (1 = present, 0 = absent), and intercompartmental connectivity.

C	S (μm^2)	Vol (μm^3)	r_{ER}	r_m	Mito.	Connections
1	5.03E+03	3.35E+04	1.50E-01	5.05E-07	1	2
2	1.26E+01	1.26E+01	1.50E-01	1.42E-03	0	1&3
3	1.19E+01	1.13E+01	1.50E-01	1.61E-03	1	2&4
4	1.12E+01	1.00E+01	1.50E-01	1.84E-03	0	3&5
5	1.05E+01	8.84E+00	1.50E-01	2.13E-03	1	4&6
6	9.86E+00	7.74E+00	1.49E-01	2.49E-03	0	5&7
7	9.18E+00	6.71E+00	1.49E-01	2.96E-03	1	6&8
8	8.51E+00	5.76E+00	1.49E-01	3.56E-03	0	7&9
9	7.83E+00	4.88E+00	1.49E-01	4.36E-03	1	8&10
10	7.16E+00	4.07E+00	1.49E-01	5.42E-03	0	9&11
11	6.48E+00	3.34E+00	1.48E-01	6.87E-03	1	10&12
12	5.80E+00	2.68E+00	1.48E-01	8.89E-03	0	11&13
13	5.13E+00	2.09E+00	1.47E-01	1.18E-02	1	12&14
14	4.45E+00	1.58E+00	1.46E-01	1.62E-02	0	13&15
15	3.77E+00	1.13E+00	1.45E-01	2.32E-02	1	14&16
16	3.10E+00	7.64E-01	1.42E-01	3.53E-02	0	15&17&21
17	2.42E+00	4.67E-01	1.35E-01	5.90E-02	1	16&18
18	1.75E+00	2.42E-01	1.20E-01	1.16E-01	0	17&19
19	1.07E+00	9.09E-02	7.46E-02	3.12E-01	0	18&20
20	3.93E-01	1.23E-02	1.03E-04	2.34E+00	0	19
21	2.42E+00	4.67E-01	1.35E-01	5.90E-02	1	16&22
22	1.75E+00	2.42E-01	1.20E-01	1.16E-01	0	21&23
23	1.07E+00	9.09E-02	7.46E-02	3.12E-01	0	22&24
24	3.93E-01	1.23E-02	1.03E-04	2.34E+00	0	23

Table S6: **Summary of the compartmental parameters used for the same-size compartment model.** Data specifying surface area, volume, cytosol–ER and cytosol–mitochondria volume fractions, and intercompartmental connectivity.

**C**	**S (μm^2)**	**Vol (μm^3)**	$\mathbf{r_{ER}}$	$\mathbf{r_m}$	**Connections**
1	1.26E+01	1.26E+01	1.50E-01	1.42E-03	2
2	1.26E+01	1.26E+01	1.50E-01	1.42E-03	1&2
3	1.26E+01	1.26E+01	1.50E-01	1.42E-03	2&4
4	1.26E+01	1.26E+01	1.50E-01	1.42E-03	3&5
5	1.26E+01	1.26E+01	1.50E-01	1.42E-03	4&6
6	1.26E+01	1.26E+01	1.50E-01	1.42E-03	5&7
7	1.26E+01	1.26E+01	1.50E-01	1.42E-03	6&8
8	1.26E+01	1.26E+01	1.50E-01	1.42E-03	7&9
9	1.26E+01	1.26E+01	1.50E-01	1.42E-03	8&10
10	1.26E+01	1.26E+01	1.50E-01	1.42E-03	9&11
11	1.26E+01	1.26E+01	1.50E-01	1.42E-03	10&12
12	1.26E+01	1.26E+01	1.50E-01	1.42E-03	11&13
13	1.26E+01	1.26E+01	1.50E-01	1.42E-03	12&14
14	1.26E+01	1.26E+01	1.50E-01	1.42E-03	13&15
15	1.26E+01	1.26E+01	1.50E-01	1.42E-03	14&16
16	1.26E+01	1.26E+01	1.50E-01	1.42E-03	15&17
17	1.26E+01	1.26E+01	1.50E-01	1.42E-03	16&18
18	1.26E+01	1.26E+01	1.50E-01	1.42E-03	17&19
19	1.26E+01	1.26E+01	1.50E-01	1.42E-03	17&20
20	1.26E+01	1.26E+01	1.50E-01	1.42E-03	19&21
21	1.26E+01	1.26E+01	1.50E-01	1.42E-03	20

Table S7: **Mitochondrial distribution (1 = present, 0 = absent) in the same-size compartment model.** d_{mito} indicates the distance between compartments with mitochondria.

**C**	$d_{\mathrm{mito}} = 1$	$d_{\mathrm{mito}} = 2$	$d_{\mathrm{mito}} = 3$	$d_{\mathrm{mito}} = 4$	$d_{\mathrm{mito}} = 5$
1	1	1	1	1	1
2	1	0	0	0	0
3	1	1	0	0	0
4	1	0	1	0	0
5	1	1	0	1	0
6	1	0	0	0	1
7	1	1	1	0	0
8	1	0	0	0	0
9	1	1	0	1	0
10	1	0	1	0	0
11	1	1	0	0	1
12	1	0	0	0	0
13	1	1	1	1	0
14	1	0	0	0	0
15	1	1	0	0	0
16	1	0	1	0	1
17	1	1	0	1	0
18	1	0	0	0	0
19	1	1	1	0	0
20	1	0	0	0	0
21	1	1	0	1	1

Table S8: **Summary of the compartmental parameters used for the unipolar morphology changing the size of the mitochondria.** Data specifying surface area, volume, cytosol–ER and cytosol–mitochondria volume fractions, size of mitochondrial cluster (0 = absent), and inter-compartmental connectivity.

**C**	**S (µm^2)**	**Vol (µm^3)**	$\mathbf{r_{ER}}$	$\mathbf{r_m}$	**Mito.**	**Connections**
1	5.03E+03	3.35E+04	1.50E-01	5.05E-07	39	2
2	1.26E+01	1.26E+01	1.50E-01	—	0	1&3
3	1.19E+01	1.13E+01	1.50E-01	1.61E-03	3	2&4
4	1.12E+01	1.00E+01	1.50E-01	—	0	3&5
5	1.05E+01	8.84E+00	1.50E-01	2.13E-03	3	4&6
6	9.86E+00	7.74E+00	1.49E-01	—	0	5&7
7	9.18E+00	6.71E+00	1.49E-01	2.96E-03	3	6&8
8	8.51E+00	5.76E+00	1.49E-01	—	0	7&9
9	7.83E+00	4.88E+00	1.49E-01	4.36E-03	3	8&10
10	7.16E+00	4.07E+00	1.49E-01	—	0	9&11
11	6.48E+00	3.34E+00	1.48E-01	6.87E-03	3	10&12
12	5.80E+00	2.68E+00	1.48E-01	—	0	11&13
13	5.13E+00	2.09E+00	1.47E-01	1.18E-02	3	12&14
14	4.45E+00	1.58E+00	1.46E-01	—	0	13&15
15	3.77E+00	1.13E+00	1.45E-01	2.32E-02	3	14&16
16	3.10E+00	7.64E-01	1.42E-01	—	0	15&17
17	2.42E+00	4.67E-01	1.35E-01	5.90E-02	3	16&18
18	1.75E+00	2.42E-01	1.20E-01	—	0	17&19
19	1.07E+00	9.09E-02	7.46E-02	—	0	18&20
20	3.93E-01	1.23E-02	1.03E-04	—	0	19
