# Supplementary material for: Context-Dependent modulation of astrocytic Ca2+ signals by mitochondria: a computational study
Source: J Comput Neurosci. 2026 Sep 25;54(4):743–62. doi: 10.1007/s10827-026-00956-3 (PMC13710611; doi:10.1007/s10827-026-00956-3)
Supplement: Supplementary file 3 [file 10827_2026_956_MOESM3_ESM.pdf]

Context-Dependent Modulation of Astrocytic Ca^{2+} Signals by Mitochondria - A Computational Study
Online Resource 3: Parameters

Thiago Ohno Bezerra[1], Antonio C. Roque[1,*]

[1] Department of Physics, School of Philosophy, Sciences and Letters of Ribeirão Preto, University of São Paulo, Bandeirantes avenue, Ribeirão Preto, 14040-900, São Paulo, Brazil
* Corresponding author. E-mail: antonior@usp.br;

Table S9: **Detailed Model Parameters.**

Parameter	Value	Unit	Reference
Resting State			
$[Ca^{2+}]_i$	0.073	µM	[1]
$[Ca^{2+}]_{ER}$	23.3480	µM	[2]
$[Ca^{2+}]_m$	0.3	µM	[3, 4]
$[Ca^{2+}]_e$	1800	µM	[1]
$[Na^+]_i$	15000	µM	[1]
$[Na^+]_e$	145000	µM	[1]
$[K^+]_i$	100000	µM	[1]
$[K^+]_e$	3000	µM	[1]
$[IP_3]$	0.1917	µM	[2]
h	0.8028	—	[2]
v	-85	mV	[1]
[Glu]	0	µM	—
[DA]	0	µM	—
IP_3 Dynamics			
PLCβ Synthesis			
K_p	10	µM	[5]
K_π	0.6	µM	[5]
PLCδ Synthesis			
v_δ	0.025	µM/s	[6]
κ_δ	1.5	µM	[5]
$K_{PLC\delta}$	0.1	µM	[5]
IP_3-3K Degradation			
v_{3K}	2	µM/s	[5]

K_D	0.7	µM	[5]
K_3	1	µM	[5]
IP-5P Degradation			
r_{5P}	0.04	1/s	[5]
Glutamate Transmission			
ρ_{Glu}	0.5	µM	—
G_{Glu}	100	1/s	[7]
K_R	1.3	µM	[5]
v_β	0.674	µM/s	—
α	0.7	—	[5]
Dopamine Transmission			
ρ_{DA}	3	µM	[8]
G_{DA}	4.201	1/s	[9]
v_{DA}	0.025	µM/s	[10]
K_{DA}	5	µM	[10]
β	0.5	—	[10]
ER Leak Current			
r_L	0.11	1/s	[5]
SERCA Current			
v_{ER}	11.93	µM/s	[6]
K_{ER}	0.1	µM	[6]
h Dynamics			
d_1	0.13	µM	[5]
d_5	0.08234	µM	[5]
d_2	1.049	µM	[5]
d_3	0.9434	µM	[5]
a_2	0.2	1/(µM s)	[5]
r_C	6	1/s	[5]
GluT			
J_{GluTmax}	0.68	pA/µm^2	[1]
K_{GluTmN}	15000	µM	[1]
K_{GluTmK}	5000	µM	[1]
K_{GluTmg}	34	µM	[1]
NKA			
J_{NKAmax}	1.52	pA/µm^2	[1]
K_{NKAmN}	10000	µM	[1]
K_{NKAmK}	1500	µM	[1]
NCX			
J_{NCXmax}	0.0001	pA/µm^2	[1]
K_{NCXmN}	87,500	µM	[1]
K_{NCXmC}	1,380	µM	[1]
k_{sat}	0.1	—	[1]

η	0.35	—	[1]
Mitochondria			
MCU			
V_{MCUmax}	165×10^{6}	1/s	[3, 4]
K_{MCU}	10	µM	[3, 4]
mPTP			
V_{mPTPmax}	300×10^{-6}	µM / s	[3, 4]
K_{mPTP}	3	µM	[3, 4]
l_{mPTP}	-2.707	1/s	—
Voltage Parameter			
C_m	0.01	F/m^2	[1]
Leak Currents			
g_{NaL}	13.482808	S/m^2	[1]
E_{Na}	61	mV	[1]
g_{KL}	145.814171	S/m^2	[1]
E_{K}	-94	mV	[1]
Diffusion Coefficients			
D_{Ca}	0.2	$\mu\text{m}^2/\text{s}$	[11]
D_{CaER}	0.001	$\mu\text{m}^2/\text{s}$	[6]
D_{IP_3}	0.2	$\mu\text{m}^2/\text{s}$	[11]
D_{Na}	0.316	$\mu\text{m}^2/\text{s}$	[12]
D_{K}	0.938	$\mu\text{m}^2/\text{s}$	[12]
D_{Cao}	4.52	$\mu\text{m}^2/\text{s}$	[13]
D_{Nao}	26.6	$\mu\text{m}^2/\text{s}$	[12]
D_{Ko}	1.732	$\mu\text{m}^2/\text{s}$	[12]
D_{glu}	4×10^{-4}	$\mu\text{m}^2/\text{s}$	[11]
D_{DA}	13.8	$\mu\text{m}^2/\text{s}$	[14]
Physical Constants			
T	303.16	K	—
F	96500	C/mol	—
R	8.314	J/mol.K	—
Ca^{2+} Threshold			
$[\text{Ca}^{2+}]_{\text{th}}$	0.15	µM	[6]

References

1. Oschmann F, Mergenthaler K, Jungnickel E, and Obermayer K. Spatial separation of two different pathways accounting for the generation of calcium signals in astrocytes. PLoS Comput Biol 2017; 13:e1005377

2. Bezerra TO and Roque AC. Dopamine facilitates the response to glutamatergic inputs in astrocyte cell models. PLOS Comput Biol 2024; 20:e1012688
3. Friel D and Tsien R. An FCCP-sensitive Ca2+ store in bullfrog sympathetic neurons and its participation in stimulus-evoked changes in [Ca2+] i. J Neurosci 1994; 14:4007–24
4. Pivovarova NB, Hongpaisan J, Andrews SB, and Friel DD. Depolarization-induced mitochondrial Ca accumulation in sympathetic neurons: spatial and temporal characteristics. J Neurosci 1999; 19:6372–84
5. De Pittà M, Goldberg M, Volman V, Berry H, and Ben-Jacob E. Glutamate regulation of calcium and IP 3 oscillating and pulsating dynamics in astrocytes. J Biol Phys 2009; 35:383–411
6. Gordleeva SY, Ermolaeva AV, Kastalskiy IA, and Kazantsev VB. Astrocyte as spatiotemporal integrating detector of neuronal activity. Front Physiol 2019; 10:294
7. Nevalainen N, Lundblad M, Gerhardt GA, and Strömberg I. Striatal glutamate release in L-DOPA-induced dyskinetic animals. PLoS One 2013; 8:e55706
8. Garris PA, Collins LB, Jones SR, and Wightman RM. Evoked extracellular dopamine in vivo in the medial prefrontal cortex. J Neurochem 1993; 61:637–47
9. Holloway ZR, Freels TG, Comstock JF, Nolen HG, Sable HJ, and Lester DB. Comparing phasic dopamine dynamics in the striatum, nucleus accumbens, amygdala, and medial prefrontal cortex. Synapse 2019; 73:e22074
10. Liu C, Goel P, and Kaeser PS. Spatial and temporal scales of dopamine transmission. Nat Rev Neurosci 2021; 22:345–58
11. Verisokin AY, Verveyko DV, Postnov DE, and Brazhe AR. Modeling of astrocyte networks: toward realistic topology and dynamics. Front Cell Neurosci 2021; 15:645068
12. McLennan H. The diffusion of potassium, sodium, sucrose and inulin in the extracellular spaces of mammalian tissues. Biochim Biophys Acta 1957; 24:1–8
13. Hrabětová S, Masri D, Tao L, Xiao F, and Nicholson C. Calcium diffusion enhanced after cleavage of negatively charged components of brain extracellular matrix by chondroitinase ABC. J Physiol 2009; 587:4029–49
14. Nicholson C. Interaction between diffusion and Michaelis-Menten uptake of dopamine after iontophoresis in striatum. Biophys J 1995; 68:1699–715
